# Lack of association between classical HLA genes and asymptomatic SARS-CoV-2 infection

**DOI:** 10.1016/j.xhgg.2024.100300

**Published:** 2024-04-26

**Authors:** Astrid Marchal, Elizabeth T. Cirulli, Iva Neveux, Evangelos Bellos, Ryan S. Thwaites, Kelly M. Schiabor Barrett, Yu Zhang, Ivana Nemes-Bokun, Mariya Kalinova, Andrew Catchpole, Stuart G. Tangye, András N. Spaan, Justin B. Lack, Jade Ghosn, Charles Burdet, Guy Gorochov, Florence Tubach, Pierre Hausfater, COVID Human Genetic Effort, Laurent Abel, Alessandro Aiuti, Saleh Al-Muhsen, Fahd Al-Mulla, Ali Amara, Mark S. Anderson, Evangelos Andreakos, Andrés A. Arias, Lisa M. Arkin, Hagit Baris Feldman, Paul Bastard, Alexandre Belot, Catherine M. Biggs, Dusan Bogunovic, Alexandre Bolze, Anastasiia Bondarenko, Alessandro Borghesi, Ahmed A. Bousfiha, Petter Brodin, Yenan Bryceson, Manish J. Butte, Jean-Laurent Casanova, Giorgio Casari, John Christodoulou, Aurélie Cobat, Roger Colobran, Antonio Condino-Neto, Stefan N. Constantinescu, Megan A. Cooper, Clifton L. Dalgard, Murkesh Desai, Beth A. Drolet, Xavier Duval, Jamila El Baghdadi, Philippine Eloy, Sara Espinosa-Padilla, Jacques Fellay, Carlos Flores, José Luis Franco, Antoine Froidure, Guy Gorochov, Peter K. Gregersen, Bodo Grimbacher, Filomeen Haerynck, David Hagin, Rabih Halwani, Lennart Hammarström, James R. Heath, Elena W.Y. Hsieh, Eystein Husebye, Kohsuke Imai, Yuval Itan, Emmanuelle Jouanguy, Elżbieta Kaja, Timokratis Karamitros, Kai Kisand, Cheng-Lung Ku, Yu-Lung Lau, Yun Ling, Carrie L. Lucas, Tom Maniatis, Davood Mansouri, László Maródi, France Mentré, Isabelle Meyts, Joshua D. Milner, Kristina Mironska, Trine H. Mogensen, Tomohiro Morio, Lisa F.P. Ng, Luigi D. Notarangelo, Antonio Novelli, Giuseppe Novelli, Cliona O'Farrelly, Satoshi Okada, Keisuke Okamoto, Tayfun Ozcelik, Qiang Pan-Hammarström, Jean W. Pape, Rebeca Perez de Diego, Jordi Perez-Tur, David S. Perlin, Graziano Pesole, Anna M. Planas, Carolina Prando, Aurora Pujol, Anne Puel, Lluis Quintana-Murci, Sathishkumar Ramaswamy, Laurent Renia, Igor Resnick, Carlos Rodríguez-Gallego, Vanessa Sancho-Shimizu, Anna Sediva, Mikko R.J. Seppänen, Mohammad Shahrooei, Anna Shcherbina, Ondrej Slaby, Andrew L. Snow, Pere Soler-Palacín, Vassili Soumelis, András N. Spaan, Ivan Tancevski, Stuart G. Tangye, Ahmad Abou Tayoun, Şehime Gülsün Temel, Christian Thorball, Pierre Tiberghien, Sophie Trouillet-Assant, Stuart E. Turvey, K. M. Furkan Uddin, Mohammed J. Uddin, Diederik van de Beek, Donald C. Vinh, Horst von Bernuth, Joost Wauters, Mayana Zatz, Pawel Zawadzki, Qian Zhang, Shen-Ying Zhang, COVIDeF Study Group, Serge Bureau, Yannick Vacher, Anne Gysembergh-Houal, Lauren Demerville, Abla Benleulmi-Chaachoua, Sebastien Abad, Radhiya Abassi, Abdelrafie Abdellaoui, Abdelkrim Abdelmalek, Hendy Abdoul, Helene Abergel, Fariza Abeud, Sophie Abgrall, Noemie Abisror, Marylise Adechian, Nordine Aderdour, Hakeem Farid Admane, Frederic Adnet, Sara Afritt, Helene Agostini, Claire Aguilar, Sophie Agut, Tommaso Francesco Aiello, Marc Ait Kaci, Hafid Ait Oufella, Gokula Ajeenthiravasan, Virginie Alauzy, Fanny Alby-Laurent, Lucie Allard, Marie-Alexandra Alyanakian, Blanca Amador Borrero, Sabrina Amam, Lucile Amrouche, Marc Andronikof, Dany Anglicheau, Nadia Anguel, Djillali Annane, Mohammed Aounzou, Caroline Aparicio, Gladys Aratus, Jean-Benoit Arlet, Jeremy Arzoine, Elisabeth Aslangul, Mona Assefi, Adeline Aubry, Laetitia Audiffred, Etienne Audureau, Christelle Nathalie Auger, Jean-Charles Auregan, Celine Awotar, Sonia Ayllon Milla, Delphine Azan, Laurene Azemar, Billal Azzouguen, Marwa Bachir Elrufaai, Aïda Badsi, Prissile Bakouboula, Coline Balcerowiak, Fanta Balde, Elodie Baldivia, Eliane-Flore Bangamingo, Amandine Baptiste, Fanny Baran-Marszak, Caroline Barau, Nathalie Barget, Flore Baronnet, Romain Barthelemy, Jean-Luc Baudel, Camille Baudry, Elodie Baudry, Laurent Beaugerie, Adel Belamri, Nicolas Belaube, Rhida Belilita, Pierre Bellassen, Rawan Belmokhtar, Isabel Beltran, Ruben Benainous, Mourad Benallaoua, Robert Benamouzig, Amélie Benbara, Jaouad Benhida, Anis Benkhelouf, Jihene Benlagha, Chahinez Benmostafa, Skander Benothmane, Miassa Bentifraouine, Laurence Berard, Quentin Bernier, Enora Berti, Astrid Bertier, Laure Berton, Simon Bessis, Alexandra Beurton, Celine Bianco, Clara Bianquis, Frank Bidar, Philippe Blanche, Clarisse Blayau, Alexandre Bleibtreu, Emmanuelle Blin, Coralie Bloch-Queyrat, Marie-Christophe Boissier, Diane Bollens, Marion Bolzoni, Rudy pierre Bompard, Nicolas Bonnet, Justine Bonnouvrier, Shirmonecrystal Botha, Wissam Boucenna, Fatiha Bouchama, Olivier Bouchaud, Hanane Bouchghoul, Taoueslylia Boudjebla, Noel Boudjema, Catherine Bouffard, Adrien Bougle, Meriem Bouguerra, Leila Bouras, Agnes Bourcier, Anne Bourgarit Durand, Anne Bourrier, Fabrice Bouscarat, Diane Bouvry, Nesrine Bouziri, Ons Bouzrara, Sarah Bribier, Delphine Brugier, Melanie Brunel, Eida Bui, Anne Buisson, Iryna Bukreyeva, Côme Bureau, Jacques Cadranel, Johann Cailhol, Ruxandra Calin, Clara Campos Vega, Pauline Canavaggio, Marta Cancella, Delphine Cantin, Albert Cao, Lionel Carbillon, Nicolas Carlier, Clementine Cassard, Guylaine Castor, Marion Cauchy, Olivier Cha, Benjamin Chaigne, Salima Challal, Karine Champion, Patrick Chariot, Julie Chas, Simon Chauveau, Anthony Chauvin, Clement Chauvin, Nathalie Chavarot, Kamélia Chebbout, Mustapha Cherai, Ilaria Cherubini, Amelie Chevalier, Thibault Chiarabini, Thierry Chinet, Richard Chocron, Pascaline Choinier, Juliette Chommeloux, Christophe Choquet, Laure Choupeaux, Benjamin Chousterman, Dragosmarius Ciocan, Ada Clarke, Gaëlle Clavere, Florian Clavier, Karine Clement, Sebastien Clerc, Yves Cohen, Fleur Cohen, Adrien Cohen, Audrey Coilly, Hester Colboc, Pauline Colin, Magalie Collet, Chloé Comarmond, Emeline Combacon, Alain Combes, Celine Comparon, Jean-Michel Constantin, Hugues Cordel, Anne-Gael Cordier, Adrien Costantini, Nathalie Costedoat Chalumeau, Camille Couffignal, Doriane Coupeau, Alain Creange, Yannie Cuvillier Lamarre, Charlène Da Silveira, Sandrine Dautheville Guibal El Kayani, Nathalie De Castro, Yann De Rycke, Lucie Del Pozo, Quentin Delannoy, Mathieu Delay, Robin Deleris, Juliette Delforge, Laëtitia Delphine, Noemie Demare, Sophie Demeret, Alexandre Demoule, Aurore Deniau, François Depret, Sophie Derolez, Ouda Derradji, Nawal Derridj, Vincent Descamps, Lydia Deschamps, Celine Desconclois, Cyrielle Desnos, Karine Desongins, Robin Dhote, Benjamin Diallo, Morgane Didier, Myriam Diemer, Stephane Diez, Juliette Djadi-Prat, Fatima-Zohra Djamouri Monnory, Siham Djebara, Naoual Djebra, Minette Djietcheu, Hadjer Djillali, Nouara Djouadi, Severine Donneger, Catarina Dos Santos, Nathalie Dournon, Martin Dres, Laura Droctove, Marie Drogrey, Margot Dropy, Elodie Drouet, Valérie Dubosq, Evelyne Dubreucq, Estelle Dubus, Boris Duchemann, Thibault Duchenoy, Emmanuel Dudoignon, Romain Dufau, Florence Dumas, Clara Duran, Emmanuelle Duron, Antoine Durrbach, Claudine Duvivier, Nathan Ebstein, Jihane El Khalifa, Alexandre Elabbadi, Caroline Elie, Gabriel Ernotte, Anne Esling, Martin Etienne, Xavier Eyer, Muriel Sarah Fartoukh, Takoua Fayali, Marion Fermaut, Arianna Fiorentino, Souha Fliss, Marie-Céline Fournier, Benjamin Fournier, Hélène Francois, Olivia Freynet, Yvann Frigout, Isaure Fromont, Axelle Fuentes, Thomas Furet, Joris Galand, Marc Garnier, Agnes Gaubert, Stéphane Gaudry, Samuel Gaugain, Damien Gauthier, Maxime Gautier, Sophie Georgin-Lavialle, Daniela Geromin, Mohamed Ghalayini, Bijan Ghaleh, Myriam Ghezal, Aude Gibelin, Linda Gimeno, Benoit Girard, Bénédicte Giroux Leprieur, Doryan Gomes, Elisabete Gomes-Pires, Guy Gorochov, Anne Gouge, Amel Gouja, Helene Goulet, Sylvain Goupil, Jeanne Goupil De Bouille, Julien Gras, Segolene Greffe, Lamiae Grimaldi, Paul Guedeney, Bertrand Guidet, Matthias Guillo, Mariechristelle Gulczynski, Tassadit Hadjam, Didier Haguenauer, Soumeya Hammal, Nadjib Hammoudi, Olivier Hanon, Anarole Harrois, Pierre Hausfater, Coraline Hautem, Guillaume Hekimian, Nicholas Heming, Olivier Hermine, Sylvie Ho, Marie Houllier, Benjamin Huot, Tessa Huscenot, Wafa Ibn Saied, Ghilas Ikherbane, Meriem Imarazene, Patrick Ingiliz, Lina Iratni, Stephane Jaureguiberry, Jean-Francois Jean-Marc, Deleena Jeyarajasingham, Pauline Jouany, Veronique Jouis, Clement Jourdaine, Ouifiya Kafif, Rim Kallala, Sandrine Katsahian, Lilit Kelesyan, Vixra Keo, Flora Ketz, Warda Khamis, Enfel Khelili, Mehdi Khellaf, Christy Gaëlla Kotokpo Youkou, Ilias Kounis, Gaelle Kpalma, Jessica Krause, Vincent Labbe, Karine Lacombe, Jean-Marc Lacorte, Anne Gaelle Lafont, Emmanuel Lafont, Lynda Lagha, Lionel Lamhaut, Aymeric Lancelot, Cecilia Landman, Fanny Lanternier, Cecile Larcheveque, Caroline Lascoux Combe, Ludovic Lassel, Benjamin Laverdant, Christophe Lavergne, Jean-Rémi Lavillegrand, Pompilia Lazureanu, Loïc Le Guennec, Lamia Leberre, Claire Leblanc, Marion Leboyer, Francois Lecomte, Marine Lecorre, Romain Leenhardt, Marylou Lefebvre, Bénédicte Lefebvre, Paul Legendre, Anne Leger, Laurence Legros, Justyna Legrosse, Sébastien Lehuunghia, Julien Lemarec, Jeremie Leporrier-Ext, Manon Lesein, Hubert Lesur, Vincent Levy, Albert Levy, Edwige Lopes, Amanda Lopes, Vanessa Lopez, Julien Lopinto, Olivier Lortholary, Badr Louadah, Bénédicte Loze, Marie-Laure Lucas, Axelle Lucasamichi, Liem Binh Luong, Arouna Magazimama-Ext, David Maingret, Lakhdar Mameri, Philippe Manivet, Cylia Mansouri, Estelle Marcault, Jonathan Marey, Nathalie Marin, Clémence Marois, Olivier Martin, Lou Martineau, Cannelle Martinez-Lopez, Pierre Martyniuck, Pauline Mary De Farcy, Nessrine Marzouk, Rafik Masmoudi, Alexandre Mebazaa, Frédéric Mechai, Fabio Mecozzi, Chamseddine Mediouni, Bruno Megarbane, Mohamed Meghadecha, Élodie Mejean, Arsene Mekinian, Nour Mekki Abdelhadi, Rania Mekni, Thinhinan Sabrina Meliti, Breno Melo Lima, Paris Meng, Soraya Merbah, Fadhila Messani, Yasmine Messaoudi, Baboo-Irwinsingh Mewasing, Lydia Meziane, Carole Michelot-Burger, Françoise Mignot, Fadi Hillary Minka, Makoto Miyara, Pierre Moine, Jean-Michel Molina, Anaïs Montegnies-Boulet, Alexandra Monti, Claire Montlahuc, Anne-Lise Montout, Alexandre Moores, Caroline Morbieu, Helene Mortelette, Stéphane Mouly, Rosita Muzaffar, Cherifa Iness Nacerddine, Marine Nadal, Hajer Nadif, Kladoum Nassarmadji, Pierre Natella, Sandrine Ndingamondze, Stefan Neraal, Caroline Nguyen, Bao N'Guyen, Isabelle Nion Larmurier, Luc Nlomenyengue, Nicolas Noel, Hilario Nunes, Edris Omar, Zineb Ouazene, Elise Ouedraogo, Wassila Ouelaa, Anissa Oukhedouma, Yasmina Ould Amara, Herve Oya, Johanna Oziel, Thomas Padilla, Elena Paillaud, Solenne Paiva, Beatrice Parfait, Perrine Parize, Christophe Parizot, Antoine Parrot, Arthur Pavot, Laetitia Peaudecerf, Frédéric Pene, Marion Pepin, Julie Pernet, Claire Pernin, Mylène Petit, Olivier Peyrony, Marie-Pierre Pietri, Olivia Pietri, Marc Pineton De Chambrun, Michelle Pinson, Claire Pintado, Valentine Piquard, Christine Pires, Benjamin Planquette, Sandrine Poirier, Anne-Laure Pomel, Stéphanie Pons, Diane Ponscarme, Annegaelle Pourcelot, Valérie Pourcher, Anne Pouvaret, Florian Prever, Miresta Previlon, Margot Prevost, Marie-Julie Provoost, Cyril Quemeneur, Cédric Rafat, Agathe Rami, Brigitte Ranque, Maurice Raphael, Jean Herle Raphalen, Anna Rastoin, Mathieu Raux, Amani Rebai, Michael Reby, Alexis Regent, Asma Regrag, Matthieu Resche-Rigon, Quentin Ressaire, Christian Richard, Mariecaroline Richard, Maxence Robert, Benjamin Rohaut, Camille Rolland-Debord, Jacques Ropers, Anne-Marie Roque-Afonso, Charlotte Rosso, Mélanie Rousseaux, Nabila Rousseaux, Swasti Roux, Lorène Roux, Claire Rouzaud, Antoine Rozes, Emma Rubenstein, Jean-Marc Sabate, Sheila Sabet, Sophie-Caroline Sacleux, Nathalie Saidenberg Kermanach, Faouzi Saliba, Dominique Salmon, Laurent Savale, Guillaume Savary, Rebecca Sberro, Anne Scemla, Frederic Schlemmer, Mathieu Schwartz, Saïd Sedfi, Samia Sefir-Kribel, Philippe Seksik, Pierre Sellier, Agathe Selves, Nicole Sembach, Luca Semerano, Marie-Victoire Senat, Damien Sene, Alexandra Serris, Lucile Sese, Naima Sghiouar, Johanna Sigaux, Martin Siguier, Johanne Silvain, Noémie Simon, Tabassome Simon, Lina Innes Skandri, Miassa Slimani, Aurélie Snauwaert, Harry Sokol, Heithem Soliman, Nisrine Soltani, Benjamin Soyer, Gabriel Steg, Lydia Suarez, Tali-Anne Szwebel, Kossi Taffame, Yacine Tandjaoui-Lambiotte, Claire Tantet, Mariagrazia Tateo, Igor Theodose, Pierre clement Thiebaud, Caroline Thomas, Kelly Tiercelet, Julie Tisserand, Carole Tomczak, Krystel Torelino, Fatima Touam-Ext, Lilia Toumi, Gustave Toury, Mireille Toy-Miou, Olivia Tran Dinh Thanh Lien, Alexy Trandinh, Jean-Marc Treluyer, Baptiste Trinque, Jennifer Truchot, Florence Tubach, Sarah Tubiana, Simone Tunesi, Matthieu Turpin, Agathe Turpin, Tomas Urbina, Rafael Usubillaga Narvaez, Yurdagul Uzunhan, Prabakar Vaittinadaayar, Arnaud Valent, Maelle Valentian, Nadia Valin, Hélène Vallet, Marina Vaz, Miguel-Alejandro Vazquezibarra, Benoit Vedie, Laetitia Velly, Celine Verstuyft, Cedric Viallette, Eric Vicaut, Dorothee Vignes, Damien Vimpere, Myriam Virlouvet, Guillaume Voiriot, Lena Voisot, Emmanuel Weiss, Nicolas Weiss, Anaïs Winchenne, Youri Yordanov, Lara Zafrani, Mohamad Zaidan, Wissem Zaidi, Cathia Zak, Aida Zarhrate-Ghoul, Ouassila Zatout, Suzanne Zeino, Michel Zeitouni, Naïma Zemirli, Lorene Zerah, Ounsa Zia, Marianne Ziol, Oceane Zolario, Julien Zuber, French COVID Cohort Study Group, Laurent Abel, Claire Andrejak, François Angoulvant, Delphine Bachelet, Marie Bartoli, Romain Basmaci, Sylvie Behillil, Marine Beluze, Dehbia Benkerrou, Krishna Bhavsar, Lila Bouadma, Sabelline Bouchez, Maude Bouscambert, Minerva Cervantes-Gonzalez, Anissa Chair, Catherine Chirouze, Alexandra Coelho, Camille Couffignal, Sandrine Couffin-Cadiergues, Eric d’Ortenzio, Marie-Pierre Debray, Laurene Deconinck, Dominique Deplanque, Diane Descamps, Mathilde Desvallée, Alpha Diallo, Alphonsine Diouf, Céline Dorival, François Dubos, Xavier Duval, Brigitte Elharrar, Philippine Eloy, Vincent Enouf, Hélène Esperou, Marina Esposito-Farese, Manuel Etienne, Eglantine Ferrand Devouge, Nathalie Gault, Alexandre Gaymard, Jade Ghosn, Tristan Gigante, Morgane Gilg, Jérémie Guedj, Alexandre Hoctin, Isabelle Hoffmann, Ikram Houas, Jean-Sébastien Hulot, Salma Jaafoura, Ouifiya Kafif, Florentia Kaguelidou, Sabrina Kali, Antoine Khalil, Coralie Khan, Cédric Laouénan, Samira Laribi, Minh Le, Quentin Le Hingrat, Soizic Le Mestre, Hervé Le Nagard, François-Xavier Lescure, Sophie Letrou, Yves Levy, Bruno Lina, Guillaume Lingas, Jean-Christophe Lucet, Denis Malvy, Marina Mambert, France Mentré, Amina Meziane, Hugo Mouquet, Jimmy Mullaert, Nadège Neant, Duc Nguyen, Marion Noret, Saad Nseir, Aurélie Papadopoulos, Christelle Paul, Nathan Peiffer-Smadja, Thomas Perpoint, Ventzislava Petrov-Sanchez, Gilles Peytavin, Huong Pham, Olivier Picone, Valentine Piquard, Oriane Puéchal, Christian Rabaud, Manuel Rosa-Calatrava, Bénédicte Rossignol, Patrick Rossignol, Carine Roy, Marion Schneider, Richa Su, Coralie Tardivon, Marie-Capucine Tellier, François Téoulé, Olivier Terrier, Jean-François Timsit, Christelle Tual, Sarah Tubiana, Sylvie Van Der Werf, Noémie Vanel, Aurélie Veislinger, Benoit Visseaux, Aurélie Wiedemann, Yazdan Yazdanpanah, CoV-Contact Cohort, Loubna Alavoine, Sylvie Behillil, Charles Burdet, Charlotte Charpentier, Aline Dechanet, Diane Descamps, Xavier Duval, Jean-Luc Ecobichon, Vincent Enouf, Wahiba Frezouls, Nadhira Houhou, Ouifiya Kafif, Jonathan Lehacaut, Sophie Letrou, Bruno Lina, Jean-Christophe Lucet, Pauline Manchon, Mariama Nouroudine, Valentine Piquard, Caroline Quintin, Michael Thy, Sarah Tubiana, Sylvie van der Werf, Valérie Vignali, Benoit Visseaux, Yazdan Yazdanpanah, Abir Chahine, Nawal Waucquier, Maria-Claire Migaud, Dominique Deplanque, Félix Djossou, Mayka Mergeay-Fabre, Aude Lucarelli, Magalie Demar, Léa Bruneau, Patrick Gérardin, Adrien Maillot, Christine Payet, Bruno Laviolle, Fabrice Laine, Christophe Paris, Mireille Desille-Dugast, Julie Fouchard, Denis Malvy, Duc Nguyen, Thierry Pistone, Pauline Perreau, Valérie Gissot, Carole L.E. Goas, Samatha Montagne, Lucie Richard, Catherine Chirouze, Kévin Bouiller, Maxime Desmarets, Alexandre Meunier, Marilou Bourgeon, Benjamin Lefévre, Hélène Jeulin, Karine Legrand, Sandra Lomazzi, Bernard Tardy, Amandine Gagneux-Brunon, Frédérique Bertholon, Elisabeth Botelho-Nevers, Christelle Kouakam, Leturque Nicolas, Layidé Roufai, Karine Amat, Sandrine Couffin-Cadiergues, Hélène Espérou, Samia Hendou, COVID-STORM Clinicians, Giuseppe Foti, Giuseppe Citerio, Ernesto Contro, Alberto Pesci, Maria Grazia Valsecchi, Marina Cazzaniga, Giacomo Bellani, COVID Clinicians, Jorge Abad, Giulia Accordino, Micol Angelini, Sergio Aguilera-Albesa, Aina Aguiló-Cucurull, Alessandro Aiuti, Esra Akyüz Özkan, Ilad Alavi Darazam, Jonathan Antonio Roblero Albisures, Juan C. Aldave, Miquel Alfonso Ramos, Taj Ali Khan, Anna Aliberti, Seyed Alireza Nadji, Gulsum Alkan, Suzan A. AlKhater, Jerome Allardet-Servent, Luis M. Allende, Rebeca Alonso-Arias, Mohammed S. Alshahrani, Laia Alsina, Marie-Alexandra Alyanakian, Blanca Amador Borrero, Zahir Amoura, Arnau Antolí, Romain Arrestier, Mélodie Aubart, Teresa Auguet, Iryna Avramenko, Gökhan Aytekin, Axelle Azot, Seiamak Bahram, Fanny Bajolle, Fausto Baldanti, Aurélie Baldolli, Maite Ballester, Hagit Baris Feldman, Benoit Barrou, Federica Barzaghi, Sabrina Basso, Gulsum Iclal Bayhan, Alexandre Belot, Liliana Bezrodnik, Agurtzane Bilbao, Geraldine Blanchard-Rohner, Ignacio Blanco, Adeline Blandinières, Daniel Blázquez-Gamero, Alexandre Bleibtreu, Marketa Bloomfield, Mireia Bolivar-Prados, Anastasiia Bondarenko, Alessandro Borghesi, Raphael Borie, Elisabeth Botdhlo-Nevers, Ahmed A. Bousfiha, Aurore Bousquet, David Boutolleau, Claire Bouvattier, Oksana Boyarchuk, Juliette Bravais, M. Luisa Briones, Marie-Eve Brunner, Raffaele Bruno, Maria Rita P. Bueno, Huda Bukhari, Jacinta Bustamante, Juan José Cáceres Agra, Ruggero Capra, Raphael Carapito, Maria Carrabba, Giorgio Casari, Carlos Casasnovas, Marion Caseris, Irene Cassaniti, Martin Castelle, Francesco Castelli, Martín Castillo de Vera, Mateus V. Castro, Emilie Catherinot, Jale Bengi Celik, Alessandro Ceschi, Martin Chalumeau, Bruno Charbit, Cécile Boulanger, Père Clavé, Bonaventura Clotet, Anna Codina, Yves Cohen, Roger Colobran, Cloé Comarmond, Alain Combes, Patrizia Comoli, Angelo G. Corsico, Taner Coşkuner, Aleksandar Cvetkovski, Cyril Cyrus, David Dalmau, François Danion, David Ross Darley, Vincent Das, Nicolas Dauby, Stéphane Dauger, Paul De Munte, Loic de Pontual, Amin Dehban, Geoffroy Delplancq, Alexandre Demoule, Isabelle Desguerre, Antonio Di Sabatino, Jean-Luc Diehl, Stephanie Dobbelaere, Elena Domínguez-Garrido, Clément Dubost, Olov Ekwall, Şefika Elmas Bozdemir, Marwa H. Elnagdy, Melike Emiroglu, Akifumi Endo, Emine Hafize Erdeniz, Selma Erol Aytekin, Maria Pilar Etxart Lasa, Romain Euvrard, Giovanna Fabio, Laurence Faivre, Antonin Falck, Muriel Fartoukh, Morgane Faure, Miguel Fernandez Arquero, Ricard Ferrer, Jose Ferreres, Carlos Flores, Bruno Francois, Victoria Fumadó, Kitty S.C. Fung, Francesca Fusco, Alenka Gagro, Blanca Garcia Solis, Pierre Garçon, Pascale Gaussem, Zeynep Gayretli, Juana Gil-Herrera, Laurent Gilardin, Audrey Giraud Gatineau, Mònica Girona-Alarcón, Karen Alejandra Cifuentes Godínez, Jean-Christophe Goffard, Nacho Gonzales, Luis I. Gonzalez-Granado, Rafaela González-Montelongo, Antoine Guerder, Belgin Gülhan, Victor Daniel Gumucio, Leif Gunnar Hanitsch, Jan Gunst, Marta Gut, Jérôme Hadjadj, Filomeen Haerynck, Rabih Halwani, Lennart Hammarström, Selda Hancerli, Tetyana Hariyan, Nevin Hatipoglu, Deniz Heppekcan, Elisa Hernandez-Brito, Po-ki Ho, María Soledad Holanda-Peña, Juan P. Horcajada, Sami Hraiech, Linda Humbert, Ivan F.N. Hung, Alejandro D. Iglesias, Antonio Íñigo-Campos, Matthieu Jamme, María Jesús Arranz, Marie-Thérèse Jimeno, Iolanda Jordan, Saliha Kanık-Yüksek, Yalcin Kara, Aydın Karahan, Adem Karbuz, Kadriye Kart Yasar, Ozgur Kasapcopur, Kenichi Kashimada, Sevgi Keles, Yasemin Kendir Demirkol, Yasutoshi Kido, Can Kizil, Ahmet Osman Kılıç, Adam Klocperk, Antonia Koutsoukou, Zbigniew J. Król, Hatem Ksouri, Paul Kuentz, Arthur M.C. Kwan, Yat Wah M. Kwan, Janette S.Y. Kwok, Jean-Christophe Lagier, David S.Y. Lam, Vicky Lampropoulou, Fanny Lanternier, Yu-Lung Lau, Fleur Le Bourgeois, Yee-Sin Leo, Rafael Leon Lopez, Daniel Leung, Michael Levin, Michael Levy, Romain Lévy, Zhi Li, Daniele Lilleri, Edson Jose Adrian Bolanos Lima, Agnes Linglart, Eduardo López-Collazo, José M. Lorenzo-Salazar, Céline Louapre, Catherine Lubetzki, Kwok-Cheung Lung, Charles-Edouard Luyt, David C. Lye, Cinthia Magnone, Davood Mansouri, Enrico Marchioni, Carola Marioli, Majid Marjani, Laura Marques, Jesus Marquez Pereira, Andrea Martín-Nalda, David Martínez Pueyo, Javier Martinez-Picado, Iciar Marzana, Carmen Mata-Martínez, Alexis Mathian, Larissa R.B. Matos, Gail V. Matthews, Julien Mayaux, Raquel McLaughlin-Garcia, Philippe Meersseman, Jean-Louis Mège, Armand Mekontso-Dessap, Isabelle Melki, Federica Meloni, Jean-François Meritet, Paolo Merlani, Özge Metin Akcan, Isabelle Meyts, Mehdi Mezidi, Isabelle Migeotte, Maude Millereux, Matthieu Million, Tristan Mirault, Clotilde Mircher, Mehdi Mirsaeidi, Yoko Mizoguchi, Bhavi P. Modi, Francesco Mojoli, Elsa Moncomble, Abián Montesdeoca Melián, Antonio Morales Martinez, Francisco Morandeira, Pierre-Emmanuel Morange, Clémence Mordacq, Guillaume Morelle, Stéphane J. Mouly, Adrián Muñoz-Barrera, Cyril Nafati, Shintaro Nagashima, Yu Nakagama, Bénédicte Neven, João Farela Neves, Lisa F.P. Ng, Yuk-Yung Ng, Hubert Nielly, Yeray Novoa Medina, Esmeralda Nuñez Cuadros, Semsi Nur Karabela, J. Gonzalo Ocejo-Vinyals, Keisuke Okamoto, Mehdi Oualha, Amani Ouedrani, Tayfun Özçelik, Aslinur Ozkaya-Parlakay, Michele Pagani, Qiang Pan-Hammarström, Maria Papadaki, Christophe Parizot, Philippe Parola, Tiffany Pascreau, Stéphane Paul, Estela Paz-Artal, Sigifredo Pedraza, Nancy Carolina González Pellecer, Silvia Pellegrini, Rebeca Pérez de Diego, Xosé Luis Pérez-Fernández, Aurélien Philippe, Quentin Philippot, Adrien Picod, Marc Pineton de Chambrun, Antonio Piralla, Laura Planas-Serra, Dominique Ploin, Julien Poissy, Géraldine Poncelet, Garyphallia Poulakou, Marie S. Pouletty, Persia Pourshahnazari, Jia Li Qiu-Chen, Paul Quentric, Thomas Rambaud, Didier Raoult, Violette Raoult, Anne-Sophie Rebillat, Claire Redin, Léa Resmini, Pilar Ricart, Jean-Christophe Richard, Raúl Rigo-Bonnin, Nadia Rivet, Jacques G. Rivière, Gemma Rocamora-Blanch, Mathieu P. Rodero, Carlos Rodrigo, Luis Antonio Rodriguez, Carlos Rodriguez-Gallego, Agustí Rodriguez-Palmero, Carolina Soledad Romero, Anya Rothenbuhler, Damien Roux, Nikoletta Rovina, Flore Rozenberg, Yvon Ruch, Montse Ruiz, Maria Yolanda Ruiz del Prado, Juan Carlos Ruiz-Rodriguez, Joan Sabater-Riera, Kai Saks, Maria Salagianni, Oliver Sanchez, Adrián Sánchez-Montalvá, Silvia Sánchez-Ramón, Laire Schidlowski, Agatha Schluter, Julien Schmidt, Matthieu Schmidt, Catharina Schuetz, Cyril E. Schweitzer, Francesco Scolari, Anna Sediva, Luis Seijo, Analia Gisela Seminario, Damien Sene, Piseth Seng, Sevtap Senoglu, Mikko Seppänen, Alex Serra Llovich, Mohammad Shahrooei, Anna Shcherbina, Virginie Siguret, Eleni Siouti, David M. Smadja, Nikaia Smith, Ali Sobh, Xavier Solanich, Jordi Solé-Violán, Catherine Soler, Pere Soler-Palacín, Betül Sözeri, Giulia Maria Stella, Yuriy Stepanovskiy, Annabelle Stoclin, Fabio Taccone, Yacine Tandjaoui-Lambiotte, Jean-Luc Taupin, Simon J. Tavernier, Loreto Vidaur Tello, Benjamin Terrier, Guillaume Thiery, Christian Thorball, Karolina Thorn, Caroline Thumerelle, Imran Tipu, Martin Tolstrup, Gabriele Tomasoni, Julie Toubiana, Josep Trenado Alvarez, Vasiliki Triantafyllia, Sophie Trouillet-Assant, Jesús Troya, Owen T.Y. Tsang, Liina Tserel, Eugene Y.K. Tso, Alessandra Tucci, Şadiye Kübra Tüter Öz, Matilde Valeria Ursini, Takanori Utsumi, Yurdagul Uzunhan, Pierre Vabres, Juan Valencia-Ramos, Ana Maria Van Den Rym, Isabelle Vandernoot, Valentina Velez-Santamaria, Silvia Patricia Zuniga Veliz, Mateus C. Vidigal, Sébastien Viel, Cédric Villain, Marie E. Vilaire-Meunier, Judit Villar-García, Audrey Vincent, Dimitri Van der Linden, Guillaume Voiriot, Alla Volokha, Fanny Vuotto, Els Wauters, Joost Wauters, Alan K.L. Wu, Tak-Chiu Wu, Aysun Yahşi, Osman Yesilbas, Mehmet Yildiz, Barnaby E. Young, Ufuk Yükselmiş, Mayana Zatz, Marco Zecca, Valentina Zuccaro, Jens Van Praet, Bart N. Lambrecht, Eva Van Braeckel, Cédric Bosteels, Levi Hoste, Eric Hoste, Fré Bauters, Jozefien De Clercq, Catherine Heijmans, Hans Slabbynck, Leslie Naesens, Benoit Florkin, Mary-Anne Young, Amanda Willis, Paloma Lapuente-Suanzes, Ana de Andrés-Martín, Orchestra Working Group, Laurent Abel, Matilda Berkell, Valerio Carelli, Alessia Fiorentino, Surbhi Malhotra, Alessandro Mattiaccio, Tommaso Pippucci, Marco Seri, Evelina Tacconelli, Amsterdam UMC COVID-19 Biobank, Michiel van Agtmael, Anne Geke Algera, Brent Appelman, Frank van Baarle, Diane Bax, Martijn Beudel, Harm Jan Bogaard, Marije Bomers, Peter Bonta, Lieuwe Bos, Michela Botta, Justin de Brabander, Godelieve de Bree, Sanne de Bruin, David T.P. Buis, Marianna Bugiani, Esther Bulle, Osoul Chouchane, Alex Cloherty, Mirjam Dijkstra, Dave A. Dongelmans, Romein W.G. Dujardin, Paul Elbers, Lucas Fleuren, Suzanne Geerlings, Theo Geijtenbeek, Armand Girbes, Bram Goorhuis, Martin P. Grobusch, Florianne Hafkamp, Laura Hagens, Jorg Hamann, Vanessa Harris, Robert Hemke, Sabine M. Hermans, Leo Heunks, Markus Hollmann, Janneke Horn, Joppe W. Hovius, Menno D. de Jong, Rutger Koning, Endry H.T. Lim, Niels van Mourik, Jeaninne Nellen, Esther J. Nossent, Frederique Paulus, Edgar Peters, Dan A.I. Pina-Fuentes, Tom van der Poll, Bennedikt Preckel, Jan M. Prins, Jorinde Raasveld, Tom Reijnders, Maurits C.F. J. de Rotte, Michiel Schinkel, Marcus J. Schultz, Femke A.P. Schrauwen, Alex Schuurmans, Jaap Schuurmans, Kim Sigaloff, Marleen A. Slim, Patrick Smeele, Marry Smit, Cornelis S. Stijnis, Willemke Stilma, Charlotte Teunissen, Patrick Thoral, Anissa M. Tsonas, Pieter R. Tuinman, Marc van der Valk, Denise P. Veelo, Carolien Volleman, Heder de Vries, Lonneke A. Vught, Michèle van Vugt, Dorien Wouters, A.H. Zwinderman, Matthijs C. Brouwer, W. Joost Wiersinga, Alexander P.J. Vlaar, Diederik van de Beek, NIAID-USUHS COVID Study Group, Miranda F. Tompkins, Camille Alba, Andrew L. Snow, Daniel N. Hupalo, John Rosenberger, Gauthaman Sukumar, Matthew D. Wilkerson, Xijun Zhang, Justin Lack, Andrew J. Oler, Kerry Dobbs, Ottavia M. Delmonte, Jeffrey J. Danielson, Andrea Biondi, Laura Rachele Bettini, Mariella D’Angiò, Ilaria Beretta, Luisa Imberti, Alessandra Sottini, Virginia Quaresima, Eugenia Quiros-Roldan, Camillo Rossi, Riccardo Castagnoli, Daniela Montagna, Amelia Licari, Gian Luigi Marseglia, Luigi D. Notarangelo, Clifton L. Dalgard, Shen-Ying Zhang, Qian Zhang, Christopher Chiu, Jacques Fellay, Joseph J. Grzymski, Vanessa Sancho-Shimizu, Laurent Abel, Jean-Laurent Casanova, Aurélie Cobat, Alexandre Bolze

**Affiliations:** 1 Laboratory of Human Genetics of Infectious Diseases, Necker Branch, INSERM U1163, Paris, France; 2 University Paris Cité, Imagine Institute, Paris, France; 3 Helix, San Mateo, CA, USA; 4 Department of Internal Medicine, University of Nevada School of Medicine, Reno, NV, USA; 5 Department of Infectious Disease, Imperial College London, London, UK; 6 National Heart and Lung Institute, Imperial College London, London, UK; 7 Laboratory of Clinical Immunology and Microbiology, Division of Intramural Research, NIAID, Bethesda, MD, USA; 8 hVIVO Services Ltd, London, UK; 9 Garvan Institute of Medical Research, Darlinghurst, NSW, Australia; 10 School of Clinical Medicine, Faculty of Medicine and Health, UNSW Sydney, New South Wales, Australia; 11 St. Giles Laboratory of Human Genetics of Infectious Diseases, Rockefeller Branch, The Rockefeller University, New York, NY, USA; 12 Department of Medical Microbiology, University Medical Center Utrecht, Utrecht University, Utrecht, the Netherlands; 13 NIAID Collaborative Bioinformatics Resource, Frederick National Laboratory for Cancer Research, Leidos Biomedical Research Inc, Frederick, MD, USA; 14 Infection, Antimicrobials, Modelling, Evolution (IAME), INSERM, UMR1137, University Paris Cité, Paris, France; 15 AP-HP, Bichat-Claude Bernard Hospital, Infectious and Tropical Diseases Department, Paris, France; 16 AP-HP, Hôpital Bichat, Centre d'Investigation Clinique, INSERM CIC 1425, Paris, France; 17 Département Epidémiologie, Biostatistiques et Recherche Clinique, Hôpital Bichat, Assistance Publique-Hôpitaux de Paris, 75018 Paris, France; 18 Sorbonne Université, INSERM Centre d’Immunologie et des Maladies Infectieuses, CIMI-Paris, Département d’immunologie Hôpital Pitié-Salpêtrière, Assistance Publique-Hôpitaux de Paris, Paris, France; 19 Sorbonne Université, INSERM, Institut Pierre Louis d’Epidémiologie et de Santé Publique, AP-HP, Hôpital Pitié-Salpêtrière, Département de Santé Publique, Unitéde Recherche Clinique PSL-CFX, CIC-1901, Paris, France; 20 Emergency Department, Hôpital Pitié-Salpêtrière, APHP-Sorbonne Université, Paris, France; 21 GRC-14 BIOSFAST Sorbonne Université, UMR INSERM 1135, CIMI, Sorbonne Université, Paris, France; 22 Department of Anatomy, Physiology & Genetics, Uniformed Services University of the Health Sciences, Bethesda, MD, USA; 23 School of Life Sciences, École Polytechnique Fédérale de Lausanne, Lausanne, Switzerland; 24 Swiss Institute of Bioinformatics, Lausanne, Switzerland; 25 Precision Medicine Unit, Lausanne University Hospital and University of Lausanne, Lausanne, Switzerland; 26 Renown Health, Reno, NV, USA; 27 Centre for Paediatrics and Child Health, Faculty of Medicine, Imperial College London, London, UK; 28 Department of Pediatrics, Necker Hospital for Sick Children, Paris, France; 29 Howard Hughes Medical Institute, New York, NY, USA

**Keywords:** HLA, association, asymptomatic infection, COVID-19, population stratification

## Abstract

Human genetic studies of critical COVID-19 pneumonia have revealed the essential role of type I interferon-dependent innate immunity to SARS-CoV-2 infection. Conversely, an association between the *HLA-B∗15:01* allele and asymptomatic SARS-CoV-2 infection in unvaccinated individuals was recently reported, suggesting a contribution of pre-existing T cell-dependent adaptive immunity. We report a lack of association of classical HLA alleles, including *HLA-B∗15:01*, with pre-omicron asymptomatic SARS-CoV-2 infection in unvaccinated participants in a prospective population-based study in the United States (191 asymptomatic vs. 945 symptomatic COVID-19 cases). Moreover, we found no such association in the international COVID Human Genetic Effort cohort (206 asymptomatic vs. 574 mild or moderate COVID-19 cases and 1,625 severe or critical COVID-19 cases). Finally, in the Human Challenge Characterisation study, the three *HLA-B∗15:01* individuals infected with SARS-CoV-2 developed symptoms. As with other acute primary infections studied, no classical HLA alleles favoring an asymptomatic course of SARS-CoV-2 infection were identified.

## Teaser

This study shows that HLA alleles, including *HLA-B*∗15:01, are not associated with asymptomatic SARS-CoV-2 infection in two independent cohorts. These results refute previous reports. HLA alleles do not strongly influence the phenotypic outcome during the acute phase of SARS-CoV-2 infection.

## Introduction

Primary infection with SARS-CoV-2 underlies a broad spectrum of clinical manifestations in unvaccinated individuals, ranging from silent infection to lethal COVID-19 pneumonia. Rare and common human genetic variants have been associated with hypoxemic COVID-19 pneumonia.^1^^,^^2^^,^^3^^,^^4^^,^^5^^,^^6^ Inborn errors of TLR3- and/or TLR7-dependent type I interferon (IFN) immunity underlie critical COVID-19 pneumonia in 1%–5% of cases.^1^^,^^7^^,^^8^^,^^9^ Moreover, autoantibodies neutralizing type I IFN underlie at least another 15% of cases,^10^^,^^11^^,^^12^ further highlighting the key role of type I IFNs in protective immunity to SARS-CoV-2 infection in the respiratory tract. While inborn errors are preferentially found in young patients with critical COVID-19 cases, the autoantibodies are more common in the elderly.^3^ Common variants in or near genes involved in viral entry into respiratory cells or airway defense have also been associated with severe COVID-19.^5^ In contrast, only a few associations between human leukocyte antigen (HLA) alleles and COVID-19 severity were consistently reported in the many studies that tested the hypothesis that HLA genes would be associated with COVID-19 because of their well-established role in T cell responses to viruses.^13^^,^^14^ The COVID-19 Host Genetics Initiative analyzed more than 20,000 cases and 2,000,000 controls (data freeze 6) to identify their first association between an HLA allele and COVID-19 severity: *HLA-DRB1∗04:01* conferred a small decrease in the risk of critical COVID-19 (odds ratio [OR] = 0.8).^2^^,^^5^

While human genetics proved fruitful to decipher some causes of severe COVID-19, very few studies investigated why a small percentage of unvaccinated adults presented with asymptomatic SARS-CoV-2 infection.^6^ In this context, in July 2023, an association was reported between the *HLA-B∗15:01* allele and asymptomatic SARS-CoV-2 infection in unvaccinated individuals.^15^ The OR was 2.40 (95% confidence interval [CI]: 1.54–3.64) for heterozygotes, reaching 8.58 (95% CI: 1.74–34.43) in homozygotes. The authors replicated this association in a smaller independent cohort. This study further showed that T cells from HLA-B∗15:01 individuals who had not been infected with SARS-CoV-2 recognized a SARS-CoV-2 T cell epitope by cross-reactivity due to prior exposure to one of two common cold coronaviruses: OC43-CoV or HKU1-CoV.^15^ Moreover, more than 100 immunogenic SARS-CoV-2 peptides are highly similar to peptides from at least one human coronavirus (hCoV) presented by a wide range of classical HLA molecules.^16^ We, therefore, tested the hypothesis of an association between HLA alleles and asymptomatic SARS-CoV-2 infection in two independent cohorts. We aimed (1) to test the association with *HLA-B∗15:01* and (2) to identify additional HLA alleles potentially associated with asymptomatic COVID-19.

## Material and methods

### Cohorts and phenotype information

#### US prospective cohort

Participants in the US prospective cohort came from two studies: the Helix DNA Discovery Project and the Healthy Nevada Project. All enrolled participants provided written informed consent for participation and were recruited through protocols conforming to local ethics requirements. Participants were recruited before the start of the COVID-19 pandemic. We performed an online survey that we sent a few times in 2021. The survey takes about 15 min to complete and has been published in the past.^17^ We received responses from 8,125 unique Helix DNA Discovery Project participants and 9,315 unique Healthy Nevada Project participants. The participants in this cohort were 18–89+ years old, 65% were female, and 85% were of European genetic similarity. The respondents indicated whether they had been infected and whether they had been vaccinated, as well as information on exposure, reasons for testing, and comorbidities. They rated the severity and duration of their symptoms and disease. They answered questions about 24 specific symptoms known to occur after SARS-CoV-2 infection.

This cohort was previously included in the large meta-analysis of the COVID-Host Genetic initiative genome-wide association study (GWAS) for susceptibility to infection. In the data freeze 7, 1,000 cases and 10,000 controls came from this cohort representing fewer than 1% of cases and 5% of total controls.^5^ More recently, this cohort was used to identify the association between *HLA-A∗03:01* and COVID-19 mRNA vaccine side effects.^17^

#### CHGE cohort

Since the beginning of the pandemic, the COVID Human Genetic Effort (CHGE) has enrolled more than 10,000 individuals with SARS-CoV-2 infection and broad clinical manifestations from all over the world. All the enrolled participants provided written informed consent for participation and were recruited through various protocols and cohorts, including the COVIDeF cohort, the French COVID cohort, the CoV-Contact Cohort, the Orchestra Working Group, the Amsterdam UMC COVID-19 Biobank, the NIAID-USUHS COVID study, and the COVID-STORM study (see supplemental methods), aiming at recruiting both hospitalized COVID-19 cases and infected controls. The physicians classified the patients according to World Health Organization (WHO) criteria,^18^ as follows: (1) Critical cases were defined as hospitalized patients with pneumonia requiring high-flow oxygen (>6 L/min) and/or requiring admission to the intensive care unit (WHO score ≥6); (2) severe cases were defined as hospitalized patients with pneumonia requiring low-flow oxygen (WHO score = 5); (3) moderate cases were defined as hospitalized patients with pneumonia not requiring oxygen (WHO score = 4); (4) mild cases were defined as pauci-symptomatic ambulatory patients, with the presence of mild, self-healing symptoms such as cough, fever, body aches, or anosmia (WHO score = 2 or 3); and (5) asymptomatic cases were defined as infected individuals with no symptoms, having a score of 1 in the WHO clinical progression scale.^18^ The presence of infection was assessed on the basis of a positive PCR test and/or serological test and/or the presence of typical symptoms such as anosmia or ageusia after exposure to a confirmed COVID-19 case.

Some of the samples of this cohort were previously included in studies focusing on the genetic causes of life-threatening COVID-19 pneumonia.^1^^,^^7^^,^^9^ The largest overlap is with the genome-wide burden screen of rare coding variants associated with critical COVID-19 pneumonia published in 2023 in *Genome Medicine*,^7^ in which most of the critical patients of the present study were included as cases, and most of the asymptomatic and mild individuals were included as controls. By contrast, most of the moderate and severe patients have not been analyzed so far. HLA association has never been previously investigated in any of the samples of the CHGE dataset.

#### SARS-CoV-2 Human Challenge Characterisation Study

Thirty-six participants were recruited, two became seropositive to spike protein prior to study start. The 34 seronegative participants were challenged with D614G-containing pre-Alpha SARS-CoV-2, of whom 33 consented for genetic analysis and were analyzed in the present study. Additional details on the study design and participants were previously published.^19^^,^^20^ Ethics approval was obtained from the UK Health Research Authority Ad Hoc Specialist Ethics Committee (reference 20/UK/0002). Written informed consent was obtained from participants before screening and enrollment.

### Sequencing

#### US prospective cohort

DNA samples were sequenced and analyzed at Helix with the Exome+ assay, which targets the exome and a few hundred thousand non-exonic common SNPs, providing a backbone for imputation of the most common SNPs in the genome as previously described.^21^ Genotype processing was performed in Hail.^22^

For each individual, we ran a supervised ADMIXTURE^23^ algorithm with k = 5 populations using the 1000 Genomes dataset. From these admixture coefficients, we then labeled each individual with one genetic similarity using the following decision tree:

- If (ADMIX_EUR>0.85) & (ADMIX_AFR<0.1) & (ADMIX_AMR<0.1) & (ADMIX_EAS<0.1) & (ADMIX_SAS<0.1) then “Europe”
- Else If ADMIX_EAS>0.6 then “East Asia”
- Else If ADMIX_SAS>0.6 then “South Asia”
- Else If (ADMIX_AFR >0.3) & (ADMIX_EAS<0.1) & (ADMIX_SAS<0.1) & (ADMIX_AFR > ADMIX_AMR) then “Africa”
- Else If (ADMIX_AMR>0.1) & (ADMIX_EAS<0.1) & (ADMIX_SAS<0.1) then “Americas”
- Else is “Other”

Principal-component analysis (PCA) was done using a set of 184,445 coding and noncoding LD-pruned, high-quality common variants. Eigenvalues and scores were calculated using the hwe_normalized_pca function in Hail.

#### CHGE cohort

Whole-exome (WES) or whole-genome sequencing (WGS) was performed at several sequencing centers, including the Genomics Core Facility of the Imagine Institute (Paris, France), the Yale Center for Genome Analysis (USA), Macrogen (USA), Psomagen (USA), the New York Genome Center (NY, USA), the American Genome Center (USUHS, Bethesda, MD, USA), MNM Bioscience (Poland), Invitae (San Francisco, CA, USA), the Genomic Sequencing Platform Seqoia (France), the Centre National de Recherche en Génomique Humaine (CNRGH, Evry, France), the Genomics Division-ITER of the Canarian Health System sequencing hub (Canary Islands, Spain), and the AlJalila Genomics Center (Dubai). Libraries for WES were generated with the Twist and Twist Plus Human Core Exome Kit, the xGen Exome Research Panel from Integrated DNA Technologies (IDT; xGen V1 and V2), Agilent SureSelect (Human All Exon V6 and V7) panels, the SeqCap EZ MedExome Kit from Roche, the Nextera Flex for Enrichment-Exome kit, the Illumina TruSeq Exome panel, and WES custom target enrichment probes. Massively parallel sequencing was performed on HiSeq 4000, HiSeq 2500, NextSeq 550, or NovaSeq 6000 systems (Illumina).

For PCA, common variants from the gnomAD v2.1 Exome dataset were jointly genotyped with GATK GenotypeGVCFs. PCA was performed with PLINK v1.9 software on a pruned set of ∼14,600 SNPs not in linkage disequilibrium (maximum r^2^ value for linkage disequilibrium of 0.4 between pairs of SNPs), with a minor allele frequency (MAF) >1%, a call rate >99%, and *p* value for departure from Hardy-Weinberg equilibrium >10^−5^, as previously described.^24^ The ancestral origin of the patients was further inferred from the PCA, as previously described.^24^

#### SARS-CoV-2 Human Challenge Characterisation Study

WGS was performed on Illumina NovaSeq (Novogene Ltd., UK), yielding 150 bp paired-end reads. The average depth of coverage was >50x with a minimum of 31x. PCA and global ancestry inference were performed using Hail according to the protocol described by the gnomAD project.^25^

### HLA calls/imputation

#### US prospective cohort

HLA alleles were imputed for seven genes: *HLA-A, -B, -C, -DPB1, -DQA1, -DQB1*, and *-DRB1* with HIBAG using the default recommendations.^26^ Individual genotypes were imputed with the model that was the most appropriate based on the genetic similarity for each individual. Specifically, we used the African model for individuals in the Africa genetic similarity group, the Asian model for those in the East Asia or the South Asia genetic similarity groups, the European model for those in the Europe or Other genetic similarity groups, and the Hispanic model for those in the Americas genetic similarity group. Models were downloaded from https://hibag.s3.amazonaws.com/hlares_index.html. As an example, the European model was the HLA4-hg19.RData model (available: https://hibag.s3.amazonaws.com/download/hlares_param/European-HLA4.html). Probabilities greater than 0.5 were used for genotype calling. All alleles had *p* value for departure from Hardy-Weinberg equilibrium >10^−4^.

#### CHGE cohort

Classical class I and class II HLA alleles were typed from the raw WES or WGS reads with HLA∗LA software,^27^ which uses a linear projection method to align reads to a population reference graph and enables high HLA typing accuracy from WES or WGS data. Alleles with *p* value for departure from Hardy-Weinberg equilibrium <10^−4^ were excluded.

#### SARS-CoV-2 Human Challenge Characterisation Study

HLA alleles were typed from raw WGS reads with HLA∗LA software at G group resolution. Only HLA calls with a posterior probability of 100% and a minimum coverage of 20x were retained in the analysis. At the B locus, all individual calls fulfilled these filtering criteria at 2-field resolution.

These tools have been validated for their accuracy to call HLA alleles at 2-field resolution, particularly in populations of European ancestry.^26^^,^^28^^,^^29^^,^^30^ For example, the HIBAG HLA calls made at Helix for seven genes in seven European ancestry Coriell samples showed 99% concordance with the known HLA calls for these individuals. Inferred HLA alleles between HIBAG and HLA∗LA were more than 96% identical at four-digit resolution in the COVID-HGI study.^2^ Differences caused by HLA allele calling should mostly be limited to rare HLA types and populations with poor imputation references.

### HLA-WAS

We used Regenie^31^ for the genetic analysis. In brief, this method builds a whole-genome regression model based on common variants to account for the effects of relatedness and population stratification; it also accounts for situations in which there is an extreme case-control imbalance likely to lead to test statistic inflation with other analysis methods. We used the approximate Firth *p* value when the logistic regression *p* value was below 0.01. The covariates included were age group, sex, and the first five principal components, as recommended to accurately correct for population structure.^31^ We used a Bonferroni correction for multiple testing.

For the US prospective cohort, a representative set of 184,445 coding and noncoding LD-pruned, high-quality common variants were identified for the construction of PCs and the whole-genome regression model, as previously described.^21^ PCs were calculated within the European group. For CHGE, the set of ∼14,600 SNPs used for PCA within the European group was used for the whole-genome regression model.

### Meta-analysis

Results were combined by inverse variance-weighted fixed-effects meta-analysis with METAL.^32^ Effect was provided as the BETA value and the STDERR was provided as the SE.

### Power calculation

We estimated the power required to detect an effect similar to that reported by Augusto et al. with the Genetic Association Study (GAS) Power Calculator, which uses a method derived from the CaTS power calculator for two-stage association studies.^33^ Power was first estimated as a function of the OR for the specific replication of the *HLA-B∗15:01* association using the following parameters: allele frequency of 0.05; prevalence of asymptomatic infection: 0.1; Dominant inheritance model; type I error of 0.05; numbers of cases and controls according to the third definition in both cohorts. In a second step we estimated the power to detect an association in the context of an HLA-wide screen (i.e., using a type I error of 5 × 10^−4^) as a function of the OR and for various allele frequencies using a sample size either corresponding to our combined samples (400 cases and 3,000 controls) or a sample size which would be 10 times larger (4,000 cases and 30,000 controls).

### Serology

Plasma immunoglobulin (Ig)G titers for the SARS-CoV-2 Human Challenge Characterisation Study participants were determined using MesoScale Discovery Coronavirus panel 2 plates on an SQ120 instrument. Binding titers given as arbitrary units per milliliter (AU/mL) based on a kit-provided human plasma standard curve.

## Results

### HLA-wide association in the US prospective cohort

**Figure 1:**
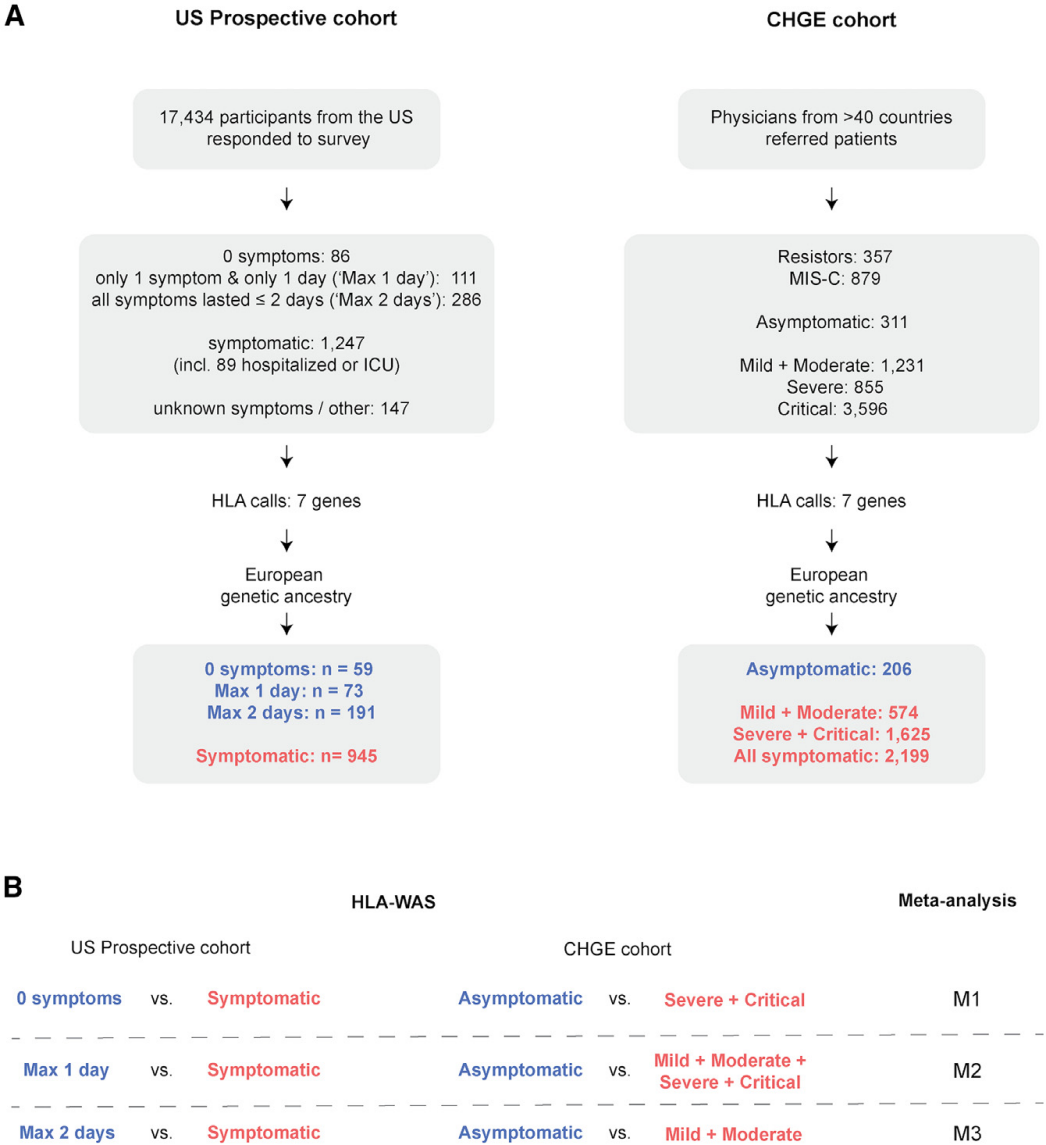
Study design (A) Description of the two cohorts and definitions of asymptomatic and symptomatic cases. ICU, intensive care unit. (B) List of HLA-wide association studies and meta-analyses performed.

We first conducted an HLA-wide association study (HLA-WAS) in a prospective population-based US cohort (Figure 1). The 17,434 adults who responded to at least one of the COVID-19 infection and vaccination surveys sent in 2021 included 1,680 participants reporting SARS-CoV-2 infection while unvaccinated. A continuous spectrum of symptoms, duration of illness was reported following SARS-CoV-2 infection (Figure S1). The most common symptoms were muscle and body aches, and a cough (Figure S1A). No symptoms at all were reported by 5.1% of individuals (*n* = 86), whereas 5.3% of the infected participants required hospitalization with or without oxygen therapy (*n* = 58) or were admitted to the intensive care unit (*n* = 31) (Figure S1B). We tested the hypothesis that HLA alleles play an important role in the early response to SARS-CoV-2 by considering three case definitions for the asymptomatic cases (Figure 1A): (1) “0 symptoms” was a stringent definition of asymptomatic as a total absence of symptoms (*n* = 86); (2) “Max 1 day” was a definition of asymptomatic in which the presence of one symptom for no more than 1 day was tolerated (*n* = 111). This definition was used to increase the power for detection of an association by enlarging the “asymptomatic” group while still identifying individuals who cleared the virus quickly and efficiently; (3) “Max 2 days” was a definition as close as possible to that used by Augusto et al., considering participants to be asymptomatic if none of their symptoms lasted 3 days or more, and if the reason for testing was unrelated to symptoms (*n* = 286).^15^ We used only one definition for controls (individuals with symptoms lasting at least 3 days). The control group included all individuals admitted to the intensive care unit or the hospital and anyone reporting symptoms of at least 3 days’ duration with some impact on their daily routine (*n* = 1,247). For the HLA-WAS, we restricted the analysis to individuals who were genetically similar to a reference group from Europe often denoted as individuals of “European ancestry” (Figures S1C and S1D), leading to a total of 59 to 191 asymptomatic cases and of 945 symptomatic controls (Figure 1A). Age and sex distribution are shown in Table S1. The association test was performed with Regenie^31^ under a dominant inheritance model, with age, sex, and the first five principal components as covariates (see supplemental methods). The risk of detecting false-positive associations was decreased by limiting the analysis to the 105 HLA alleles with an allele frequency of at least 1% in this cohort. No statistically significant associations (at a corrected threshold of *p* < 0.00048) were found with any of the three phenotype definitions (Tables 1 and S2–S4). The top-ranked HLA allele was *DRB1∗16:01*, which was depleted in asymptomatic individuals, with the strongest effect being observed in the “Max 2 days” group of asymptomatic patients (OR [95% CI] = 0.06 [0–1.5], *p* = 0.004, *p*_corrected_ = 0.42, Table 1).

**Table 1:** Top-ranked alleles in the HLA-WAS on the US prospective cohort

Allele	OR [95% CI]^a^	Raw *p* value^b^	AF^c^	Asymptomatic definition^d^
DRB1∗16:01	0.06 [0.002–1.5]	0.004	0.012	Max 2 days
A∗32:01	2.19 [1.3–3.8]	0.008	0.035	Max 2 days
B∗07:02	1.58 [1.1–2.3]	0.016	0.13	Max 2 days
C∗07:02	1.53 [1.1–2.2]	0.020	0.14	Max 2 days
DQB1∗06:02	1.52 [1.1–2.2]	0.025	0.14	Max 2 days
DQB1∗05:02	0.29 [0.1–1.1]	0.037	0.015	Max 2 days

a Odds ratio (OR) of being asymptomatic, i.e., OR >1 indicates that the allele is more frequent in asymptomatic individuals. CI = confidence interval.

b Significance threshold after Bonferroni correction for multiple testing is 0.00048. Alleles shown are those with a raw *p* value <0.05.

c Allele frequency (AF) is based on the frequency of the allele in the US prospective cohort “Max 2 days” analysis because this analysis included the largest numbers of cases and controls.

d For each allele, the top-ranked result across the three asymptomatic definitions in the US prospective cohort is given.

### HLA-wide association in the CHGE cohort

We next studied patients recruited by the physicians of the international CHGE consortium. These physicians classified participants with SARS-CoV-2 infections according to acute disease severity: asymptomatic, mild, moderate, severe, or critical (Figure S2A). Whole-exome or WGS data were available for 7,229 participants and HLA alleles were typed with HLA∗LA.^27^ In this HLA-WAS, we compared the patients classified as “asymptomatic” by the clinicians (*n* = 311) with those in three sets of symptomatic controls: (1) the patients with the most extreme symptoms requiring hospitalization and oxygen supplementation (i.e., those with a severe or critical form of the disease, *n* = 4,451); (2) all symptomatic patients, whatever their acute disease severity (i.e., mild, moderate, severe or critical, *n* = 5,682); and (3) symptomatic patients not requiring oxygen supplementation (i.e., mild and moderate patients only, *n* = 1,231); this last group of symptomatic patients is the most similar to the symptomatic patients groups of the US prospective cohort and the study by Augusto et al. (Figure 1). We restricted the analysis to individuals of European genetic ancestry and the final study population comprised 206 asymptomatic cases, 1,625 patients with severe or critical disease, and 574 patients with mild or moderate disease (Figures S2B and S2C). Age and sex distribution are shown in Table S1. Analyses were also performed under a dominant inheritance model with age group, sex, and the first five principal components as covariates. This analysis was performed with Regenie and was limited to the 114 HLA alleles with an allele frequency of at least 1% in this cohort. No statistically significant association (at a corrected threshold of *p* < 0.00044) was identified in the HLA-WAS, regardless of the definition of symptomatic patients used (Tables S5–S7). The top-ranked HLA allele found to be enriched in asymptomatic individuals was *HLA-B∗40:02*, for which the strongest effect was observed in comparison with the group of symptomatic patients with severe or critical disease (OR [95% CI] = 3.4 [1.5–7.7], *p* = 0.005; *p*_corrected_ = 0.57, Table 2).

**Table 2:** Top-ranked alleles in the HLA-WAS on the CHGE European cohort

Allele	OR [95% CI]^a^	Raw *p* value^b^	AF^c^	Controls used^d^
B∗40:02	3.42 [1.5–7.7]	0.005	0.016	Severe + Critical
DPB1∗01:01	0.28 [0.1–0.8]	0.007	0.042	Severe + Critical
A∗23:01	2.5 [1.2–5.0]	0.010	0.023	Mild + Moderate
B∗49:01	2.26 [1.2–4.3]	0.014	0.031	Mild + Moderate
A∗03:01	1.66 [1.1–2.5]	0.019	0.12	Severe + Critical
DQA1∗01:02	1.54 [1.1–2.2]	0.022	0.18	Mild + Moderate
A∗30:02	2.46 [1.1–5.6]	0.031	0.019	Mild + Moderate
B∗57:01	0.47 [0.2–1.0]	0.047	0.027	Mild + Moderate
A∗68:02	3.53 [1.0–12.2]	0.047	0.01	Mild + Moderate
DPB1∗03:01	0.65 [0.4–1.0]	0.049	0.093	Mild + Moderate

a Odds ratio (OR) of being asymptomatic, i.e., OR >1 indicates that the allele is more frequent in asymptomatic individuals. CI = confidence interval.

b Significance threshold after Bonferroni correction for multiple testing is 0.00044. Alleles shown are those with a raw *p* value <0.05.

c Allele frequency (AF) is based on the frequency of the allele in the CHGE European cohort “All” analysis, which included the largest numbers of cases and controls.

d For each allele, the top-ranked result across three sets of symptomatic patients in the CHGE European sample is given.

### HLA-wide meta-analysis

We then performed three meta-analyses, denoted M1, M2, and M3 (Figure 1B), combining the results from our two independent cohorts with METAL.^32^ The first used the strictest definitions for the groups: the HLA-WAS with the “0 symptoms” group of asymptomatic patients in the US prospective cohort and the HLA-WAS limited to patients with severe and critical disease only in the CHGE cohort (Table S8). The second meta-analysis combined the HLA-WAS with the “Max 1 day” definition of asymptomatic patients for the US prospective cohort (0 symptoms or one symptom for 1 day) with the HLA-WAS with all symptomatic cases from the CHGE (Table S9). The final meta-analysis used the results for the asymptomatic and symptomatic groups most closely resembling those of the study by Augusto et al. (Table S10). The meta-analyses detected no statistically significant associations (at a corrected threshold of *p* < 0.00053, 95 alleles tested) between HLA alleles and asymptomatic SARS-CoV-2 infection (Tables 3 and S8–S10). The top-ranked HLA allele was *HLA-B∗40:02* (*p* value = 0.0008, *p*_corrected_ = 0.095), for which enrichment was observed in asymptomatic individuals relative to symptomatic individuals in both cohorts and in the meta-analysis based on the strictest definitions. Of note, this allele has exactly the same frequency in cases and controls in the study of Augusto et al.^15^

**Table 3:** Top-ranked alleles in the meta-analyses and corresponding results in the US prospective and CHGE cohorts

Allele	Meta-analysis				US prospective cohort^c^		CHGE cohort^c^	
	Meta-analysis number	OR [95% CI]^a^	Raw *p* value^b^	Corrected *p* value	OR [95% CI]^a^	Raw *p* value	OR [95% CI]^a^	Raw *p* value
B∗40:02	M1	3.51 [1.7–7.3]	0.0008	0.095	4.05 [0.7–24.6]	0.128	3.42 [1.5–7.7]	0.005
DPB1∗01:01	M2	0.43 [0.3–0.7]	0.0015	0.17	0.43 [0.2–1.0]	0.058	0.43 [0.2–0.8]	0.010
DQA1∗01:02	M3	1.41 [1.1–1.8]	0.007	0.80	1.31 [0.9–1.8]	0.119	1.54 [1.1–2.2]	0.022
A∗23:01	M2	2.22 [1.2–4.0]	0.007	0.82	2.14 [0.7–6.6]	0.186	2.25 [1.1–4.4]	0.019
DQB1∗06:02	M3	1.46 [1.1–2.0]	0.013	1	1.52 [1.1–2.2]	0.025	1.33 [0.8–2.2]	0.276
C∗03:03	M1	0.52 [0.3–0.9]	0.015	1	0.48 [0.2–1.2]	0.132	0.54 [0.3–1.0]	0.055
B∗49:01	M3	1.96 [1.1–3.4]	0.019	1	1.29 [0.4–3.9]	0.657	2.26 [1.2–4.3]	0.014
B∗07:02	M3	1.42 [1.1–1.9]	0.021	1	1.58 [1.1–2.3]	0.016	1.16 [0.7–1.9]	0.554
DRB1∗15:01	M3	1.38 [1.0–1.9]	0.037	1	1.42 [1–2.1]	0.075	1.32 [0.8–2.2]	0.264

a Odds ratio (OR) of being asymptomatic, i.e., OR>1 indicates that the allele is more frequent in asymptomatic individuals. CI = confidence interval.

b Significance threshold after Bonferroni correction for multiple testing is 0.00053. Alleles shown are those with a raw *p* value <0.05.

c For each allele, ORs and *p* values obtained in the US prospective and CHGE cohorts with asymptomatic or controls definitions used in the corresponding meta-analysis are given.

### Lack of replication for HLA-B∗15:01

**Figure 2:**
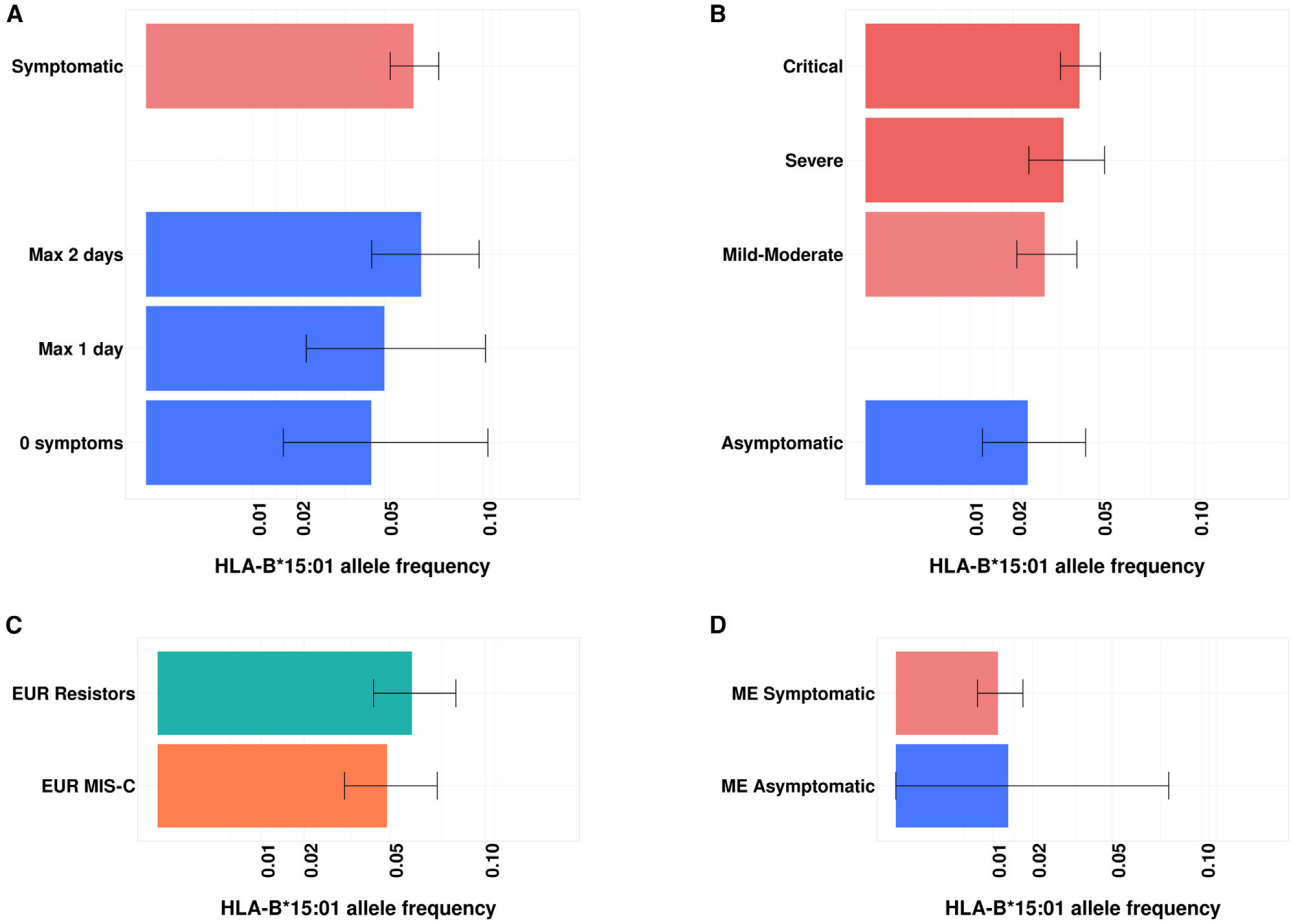
*HLA-B∗15:01* is not enriched in asymptomatic cases (A) Allele frequency and 95% confidence intervals in the US prospective cohort European subgroups. (B) Allele frequency and 95% CIs in the CHGE European sample. (C) Allele frequency and 95% CIs in individuals highly exposed to SARS-CoV-2 who never tested positive (“Resistors,” *n* = 291) and in children with SARS-CoV-2 infection complicated by multisystem inflammatory syndrome (“MIS-C,” *n* = 235) from the European CHGE sample. (D) Allele frequency and 95% CIs in Middle Eastern (ME) individuals from the CHGE cohort (Symptomatic, *n* = 895; Asymptomatic, *n* = 37).

An analysis focusing on *HLA-B∗15:01* did not replicate the association between *HLA-B∗15:01* and asymptomatic SARS-CoV-2 infection (Figures 2A and 2B; Table 4) despite being well powered (>95%) to detect an effect similar to that reported by Augusto et al. (OR of 2.40 for enrichment in asymptomatic vs. symptomatic patients, *p* = 5.67 × 10^−5^) (Figure S3). We further estimated the frequency of *HLA-B∗15:01* in various groups of patients of the CHGE consortium, including children with SARS-CoV-2 infection complicated by multisystem inflammatory syndrome (classified as MIS-C) and individuals with high levels of exposure who never tested positive (classified as “resistors”).^34^^,^^35^ This frequency ranged from 2.4% in asymptomatic individuals to 6.0% in resistors (Figure 2C). We also looked at individuals from non-European genetic ancestries. Similarly, we found no difference in frequency between asymptomatic and symptomatic individuals (Figure 2D and Table S11). Overall, no enrichment in the HLA-B∗15:01 allele was observed among asymptomatic individuals in our US population-based prospective cohort, or in the international CHGE cohort.

**Table 4:** Odds ratio for the association of *HLA-B∗15:01* with asymptomatic SARS-CoV-2 infection in both cohorts and in the meta-analysis

Analysis	AF asymptomatic cases^a^	AF symptomatic controls^a^	OR [95% CI]^b^	Raw *p* value
US prospective cohort	0.067	0.063	0.96 [0.58–1.6]	0.89
CHGE cohort	0.024	0.030	0.74 [0.36–1.53]	0.42
Meta-analysis M3	–	–	0.88 [0.58–1.34]	0.56

a Allele frequencies (AF) are based on the frequency of the allele in the US prospective cohort “Max 2 days” analysis and in in the CHGE European cohort “Mild + Moderate” analysis, combined in the meta-analysis M3.

b Odds ratio (OR) of being asymptomatic, i.e., OR >1 indicates that the allele is more frequent in asymptomatic individuals. CI = confidence interval.

### Symptoms and serology for participants with HLA-B∗15:01 in the SARS-CoV-2 Human Challenge Characterisation Study

**Figure 3:**
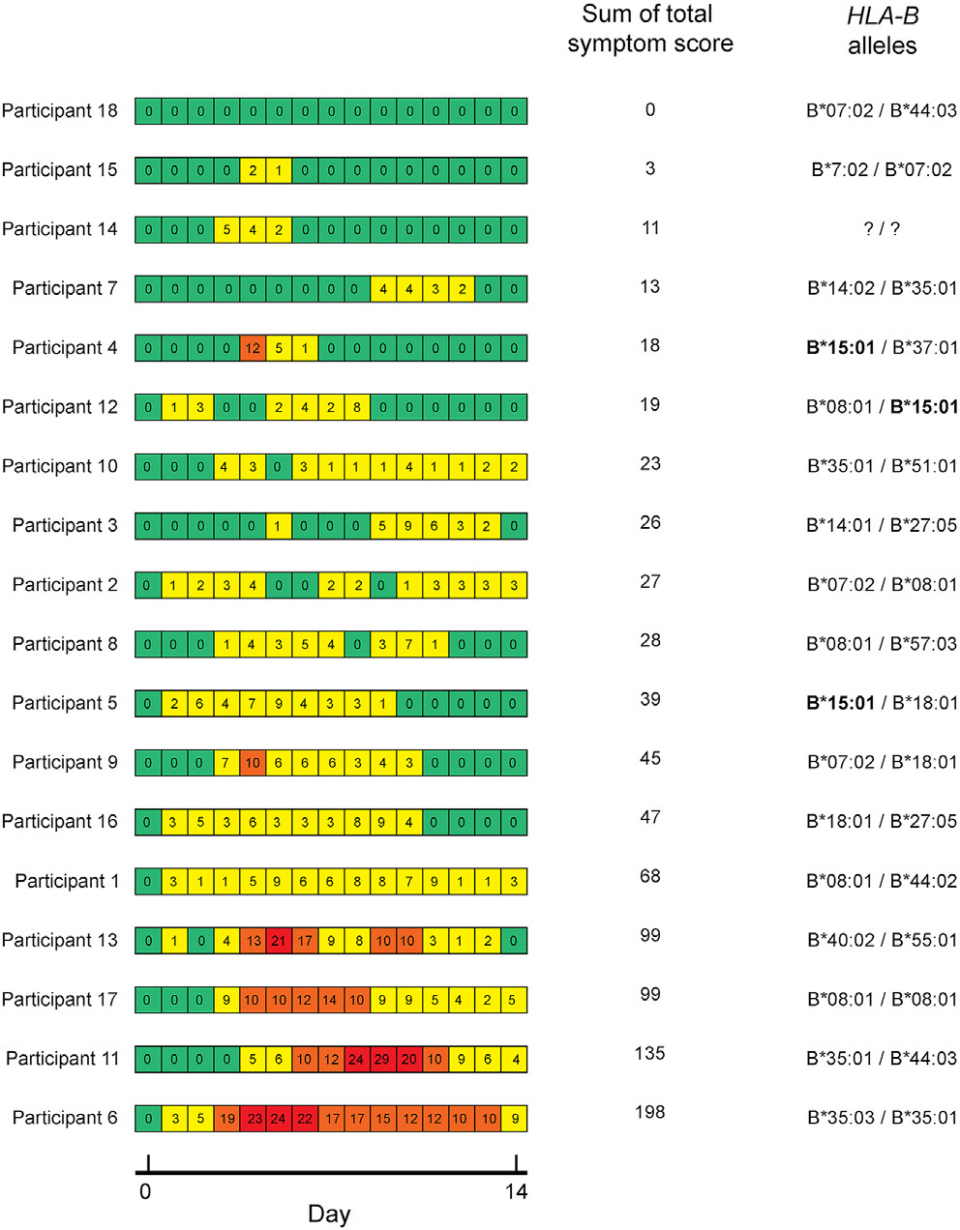
*HLA-B∗15:01* in the SARS-CoV-2 Human Challenge Characterisation Study: Symptoms and *HLA-B* genotypes for 18 infected participants Daily total symptom score was calculated using self-reported symptom diaries three times daily. Daily total symptom scores are displayed in the heatmap, ranging from green (no symptoms) to red (highest symptom score). The heatmap is derived from Figure 2 in Zhou J. et al., *Lancet Microbe* (2023).^19^

The mechanism proposed as an explanation for the association between *HLA-B∗15:01* and asymptomatic SARS-CoV-2 infection was pre-existing immunity, probably due to prior infection with OC43-CoV or HKU1-CoV.^15^ Unfortunately, no serological data were available for the HLA-B∗15:01 carriers in the US prospective and the CHGE cohorts. We tested the hypothesis that the lack of association in our study was due to an absence of prior infection with OC43-CoV or HKU1-CoV by examining the data for the SARS-CoV-2 Human Challenge Characterisation Study (ClinicalTrials.gov identifier NCT04865237; funder, UK Vaccine Taskforce), in which 34 participants seronegative to spike protein were challenged with D614G-containing pre-Alpha SARS-CoV-2, of whom 33 consented for genetic analysis.^19^ Serological data, history of prior infections with other coronaviruses, and genetic data were available, together with infection status and data concerning the recorded symptoms. HLA alleles were called with HLA∗LA from whole-genome sequences obtained from the participants. Three of the 17 infected participants (positive test result) carried an *HLA-B∗15:01* allele, as well as three of the 16 who stayed uninfected. Only one of the 17 infected participants was fully asymptomatic and this participant did not carry the *HLA-B∗15:01* allele. The three infected participants with an *HLA-B∗15:01* allele were symptomatic (Figure 3), despite evidence of prior exposure to OC43-CoV and HKU1-CoV (Figure S4). Thus, prior exposure to a coronavirus did not prevent the *HLA-B∗15:01* carriers from developing symptoms following SARS-CoV-2 infection.

## Discussion

Our analyses identified no associations between classical HLA alleles and asymptomatic SARS-CoV-2 infection. In particular, we did not replicate the previously reported association between *HLA-B∗15:01* and asymptomatic SARS-CoV-2 infection, despite being well powered (>95%) for this specific replication study. Another recent study in a Spanish cohort found no associations between classical HLA alleles and asymptomatic SARS-CoV-2 infection.^36^ One possible explanation for the difference in results regarding *HLA-B∗15:01* is that the studies analyzed different groups of individuals living in different environments. However, the US prospective cohort we analyzed has many features in common with the cohort analyzed by Augusto et al.: specifically, the participants were from the United States, with a slight bias toward women, and the phenotype was assessed on the basis of self-reported surveys at multiple time points during the pandemic before summer 2021 (before the SARS-CoV-2 Delta variant became dominant in the United States^37^). The percentage of individuals self-reporting asymptomatic infection was similar between the two, as were the rates of each symptom. Alternatively, the difference in results may reflect differences in the handling of potential population stratification. Augusto et al. did not consider population structure in their study on bone marrow donors, probably because no genetic information outside of the HLA region was available, whereas we accounted for population structure by restricting our analysis to those of European ancestry and including the first five principal components as covariates in our regression model. The highly polymorphic nature of the HLA region and the differences in allele frequencies between human sub-populations contribute to a high risk of false-positive results in association analyses. The frequency of *HLA-B∗15:01* is known to vary across continents, between continental populations within the United States (Figure S5A), and even between European countries (Figure S5B). Augusto et al. used self-identified race (White) to select their participants; however, this is not an appropriate proxy of genetic ancestry.^38^ Population stratification may, thus, have played a confounding role in their study.

Overall, the absence of an association between classical HLA alleles and asymptomatic SARS-CoV-2 infection is consistent with the modest impact of HLA variation on severe or critical COVID-19.^5^^,^^14^^,^^39^ A limitation of our study is the limited power to detect an association in the context of an HLA-wide screen, in particular due to the relatively small number of asymptomatic individuals. Using a sample size combining our two cohorts, we had an 80% power to detect an association with an allele with a minor allele frequency (MAF) of 0.05 and an OR of 2.18, or an allele with a MAF of 0.2 and an OR of 1.72 (Figure S6). With a sample size 10X bigger, we would have an 80% power to detect an association leading to an OR of 1.29 and 1.18 for an allele with a MAF of 0.05 and 0.2, respectively (Figure S6). Nonetheless, our results indicate that a potential association between an HLA allele and asymptomatic SARS-CoV-2 infection would not have a strong effect. This result is also consistent with the absence of any strong association between HLA alleles and clinical outcomes during the acute phase for the other primary viral infections studied to date.^40^^,^^41^^,^^42^ By contrast, HLA alleles have been associated with multiple clinical or laboratory outcomes during chronic infections, including viral (e.g., HIV, HBV, HCV), mycobacterial (e.g., leprosy), and protozoan infections.^42^^,^^43^^,^^44^^,^^45^ HLA alleles are also known to be associated with responses to vaccinations,^46^^,^^47^ including against COVID-19.^17^^,^^48^ While pre-existing immunity due to prior infections with common cold coronaviruses might help preventing the development of clinical manifestations following SARS-CoV-2 infection, our results suggest that such pre-existing immunity would not strongly depend on HLA alleles.

## Data and code availability

Data supporting the findings of this study are available within the manuscript and supplemental files. The WGS data of anonymized patients recruited through the National Institutes of Health (NIH) and sequenced at the National Institute of Allergy and Infectious Diseases (NIAID) through the Uniformed Services University of the Health Sciences (USUHS)/the American Genome Center (TAGC) are available under dbGaP submission phs002245.v1. Other patients were not consented to share the raw WES/WGS data files beyond the research and clinical teams.

## Web resources

HIBAG software and models: https://hibag.s3.amazonaws.com/hlares_index.html

Allele frequency Net Database: https://github.com/slowkow/allelefrequencies

HLA alleles distribution in 1000 Genomes Project: http://ftp.1000genomes.ebi.ac.uk/vol1/ftp/data_collections/HLA_types/

Genetic Association Study Power Calculator: https://csg.sph.umich.edu/abecasis/gas_power_calculator

## Supplemental information

Doucument S1. Figures S1–S6 and Supplemental methods

Consortia names affiliations

Supplemental tables S1–S11

Document S2. Article plus supplemental information
