# Supplementary material for: Lack of association between classical HLA genes and asymptomatic SARS-CoV-2 infection
Source: HGG Adv. 2024 Apr 26;5(3):100300. doi: 10.1016/j.xhgg.2024.100300 (PMC11215417; doi:10.1016/j.xhgg.2024.100300)
Supplement: Doucument S1. Figures S1–S6 and Supplemental methods [file mmc1.pdf]

HGGA, Volume *5*

Supplemental information

Lack of association between classical HLA genes and asymptomatic SARS-CoV-2 infection

Astrid Marchal, Elizabeth T. Cirulli, Iva Neveux, Evangelos Bellos, Ryan S. Thwaites, Kelly M. Schiabor Barrett, Yu Zhang, Ivana Nemes-Bokun, Mariya Kalinova, Andrew Catchpole, Stuart G. Tangye, András N. Spaan, Justin B. Lack, Jade Ghosn, Charles Burdet, Guy Gorochov, Florence Tubach, Pierre Hausfater, COVID Human Genetic Effort, COVIDeF Study Group, French COVID Cohort Study Group, CoV-Contact Cohort, COVID-STORM Clinicians, COVID Clinicians, Orchestra Working Group, Amsterdam UMC COVID-19 Biobank, NIAID-USUHS COVID Study Group, Clifton L. Dalgard, Shen-Ying Zhang, Qian Zhang, Christopher Chiu, Jacques Fellay, Joseph J. Grzymski, Vanessa Sancho-Shimizu, Laurent Abel, Jean-Laurent Casanova, Aurélie Cobat, and Alexandre Bolze

Table of content

Figure S1: COVID-19 symptoms in a US prospective cohort.

Figure S2: COVID-19 symptoms in the CHGE cohort.

Figure S3: Power curves: replication.

Figure S4: Seasonal CoV antibodies in the SARS-CoV-2 Human Challenge Characterisation Study.

Figure S5: Frequency of HLA-B*15:01 allele.

Figure S6: Power curves: HLA-WAS.

Supplemental methods: Ethics approval and consent to participate.

Table S1: Demographics of both cohorts.

Table S2: HLA-WAS US prospective cohort - asymptomatic '0 symptoms'.

Table S3: HLA-WAS US prospective cohort - asymptomatic 'Max 1 day'.

Table S4: HLA-WAS US prospective cohort - asymptomatic 'Max 2 days'.

Table S5: HLA-WAS CHGE cohort - asymptomatic vs severe and critical.

Table S6: HLA-WAS CHGE cohort - asymptomatic vs all symptomatic.

Table S7: HLA-WAS CHGE cohort - asymptomatic vs mild and moderate.

Table S8: Meta-analysis M1: US asymptomatic '0 symptoms' + CHGE severe critical.

Table S9: Meta-analysis M2: US asymptomatic 'Max 1 day' + CHGE all.

Table S10: Meta-analysis M3: US asymptomatic 'Max 2 days' + CHGE mild moderate.

Table S11: HLA-B*15:01 allele counts.

Figure S1

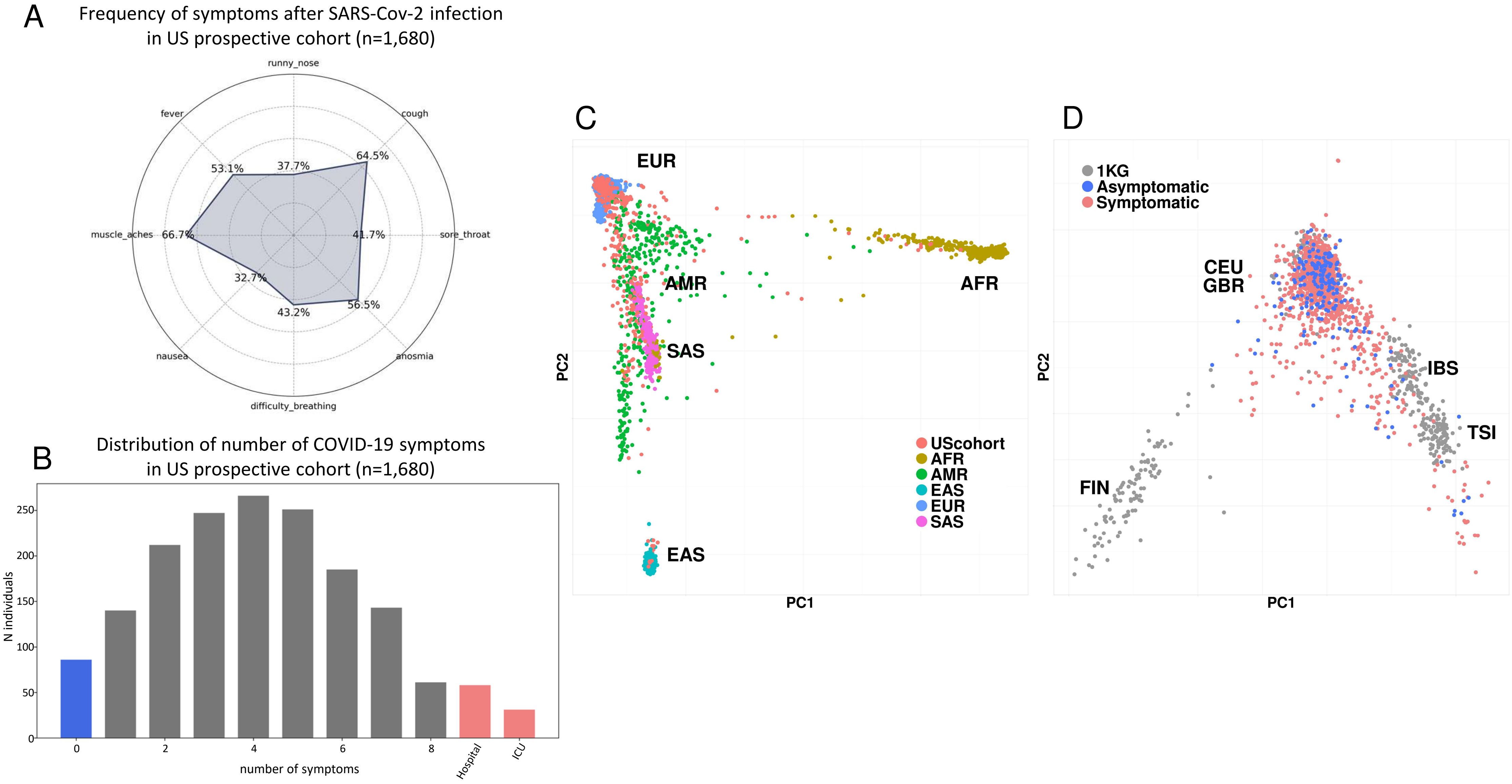

Figure S1: COVID-19 symptoms in a US prospective cohort.

A: Radar plot showing the % of infected individuals reporting each symptom.

B: Distribution of the number of symptoms per individual infected with SARS-CoV-2. Patients who had to be hospitalized or admitted to the ICU are represented separately (pink bars). Infected individuals who reporting no symptoms are represented by the blue bar.

C: PCA plot displaying the US prospective cohort (orange dots) overlapped with 1000 Genomes Project samples labeled with their known ancestry (AFR: African, AMR: American, EAS: East Asian, EUR: European, SAS: South Asian).

D: PCA plot displaying the European genetic ancestry subsets of the US prospective cohort and from 1000 Genomes Projects. Origin of 1000 Genomes Project (1KG) samples are indicated (CEU: Utah residents with Northern and Western European ancestry, FIN: Finnish in Finland, GBR: British in England and Scotland, IBS: Iberian population in Spain, TSI: Toscani in Italy).

Figure S2

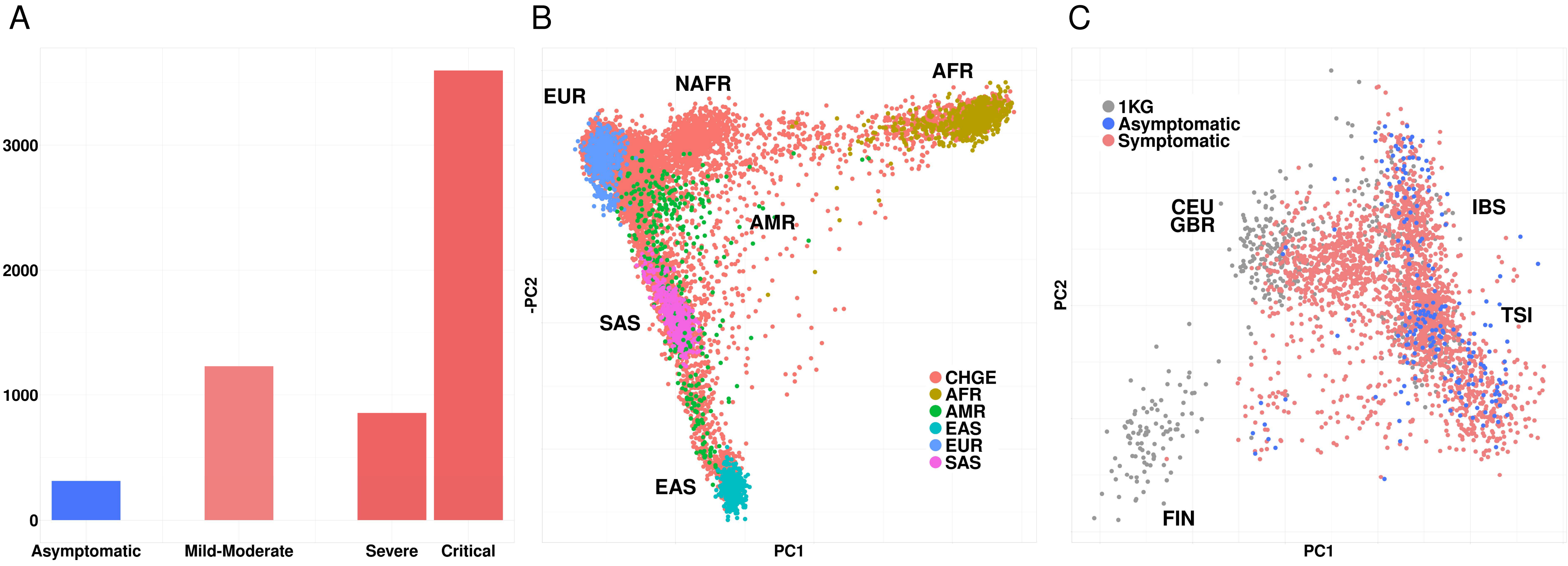

Figure S2: COVID-19 symptoms in the CHGE cohort.
A: Distribution of the number of individuals per category of severity.
B: PCA plot displaying the CHGE cohort (orange dots) overlapped with the samples from 1000 Genomes Project. Ancestry of 100 Genomes Project samples are indicated (AFR: African, AMR: American, EAS: East Asian, EUR: European, NAFR: North African, SAS: South Asian).
C: PCA plot displaying the European genetic ancestry subsets of the CHGE cohort and from 1000 Genomes Projects. Origin of 1000 Genomes Project (1KG) samples are indicated (CEU: Utah residents with Northern and Western European ancestry, FIN: Finnish in Finland, GBR: British in England and Scotland, IBS: Iberian population in Spain, TSI: Toscani in Italy).

Figure S3

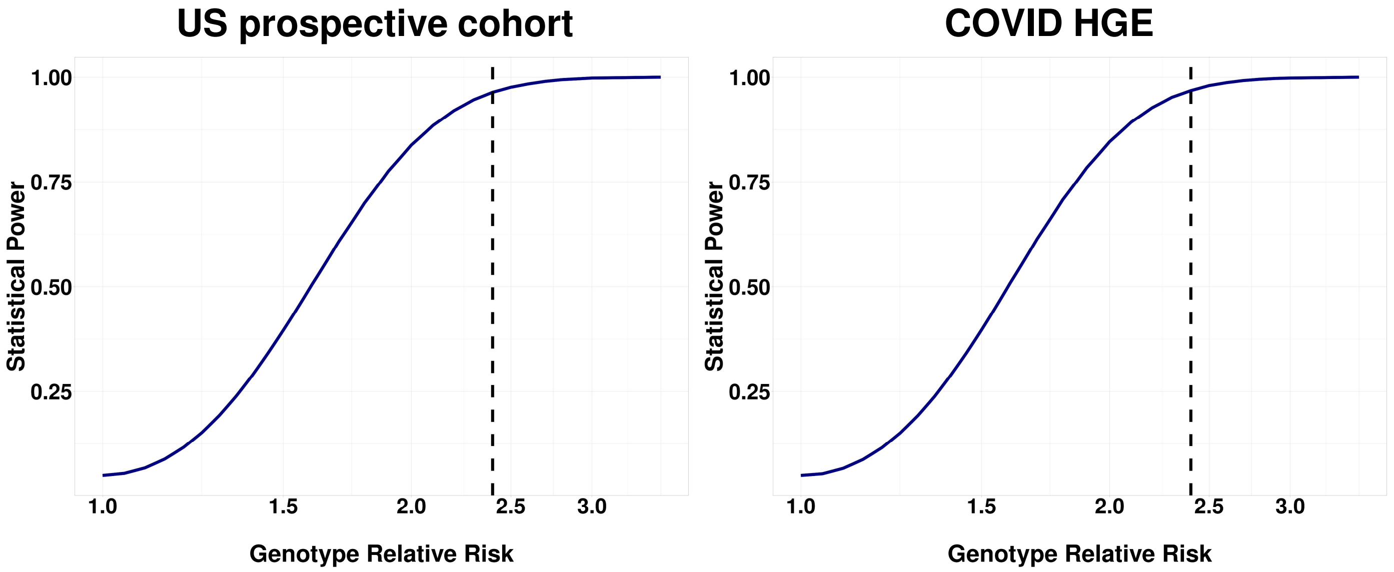

Figure S3: Power curves: replication.
Statistical power as a function of genotype relative risk, calculated under a dominant inheritance model, with a *HLA-B*15:01* frequency of 0.05, a prevalence of asymptomatic infection of 0.1, and a p-value threshold of 0.05 in a context of replication.
Curves plotted with the Genetic Association Study Power Calculator (https://csg.sph.umich.edu/abecasis/gas_power_calculator). The dotted line represent the Odds ratio obtained by Augusto, Murdolo & Chatzileontiadou et al. (OR=2.4).

Figure S4

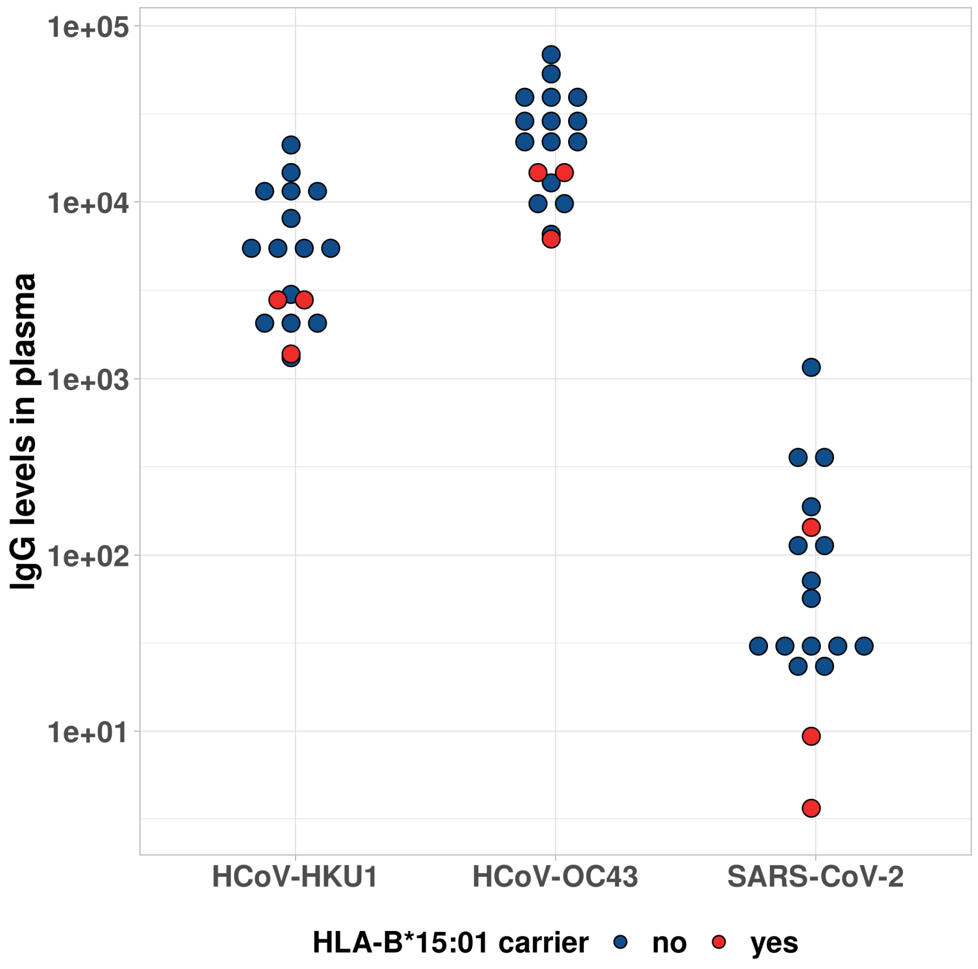

Figure S4: Seasonal CoV antibodies in the SARS-CoV-2 Human Challenge Characterisation Study.

Plasma IgG was quantified in baseline samples from the SARS-CoV-2 human challenge characterisation study participants (Infected, n=17) for HKU1-CoV Spike protein, OC43-CoV Spike protein and SARS-CoV-2 Spike protein as a negative control. Carriers of HLA-B*15:01 are indicated in red. Arbitrary units per milliliter.

Figure S5

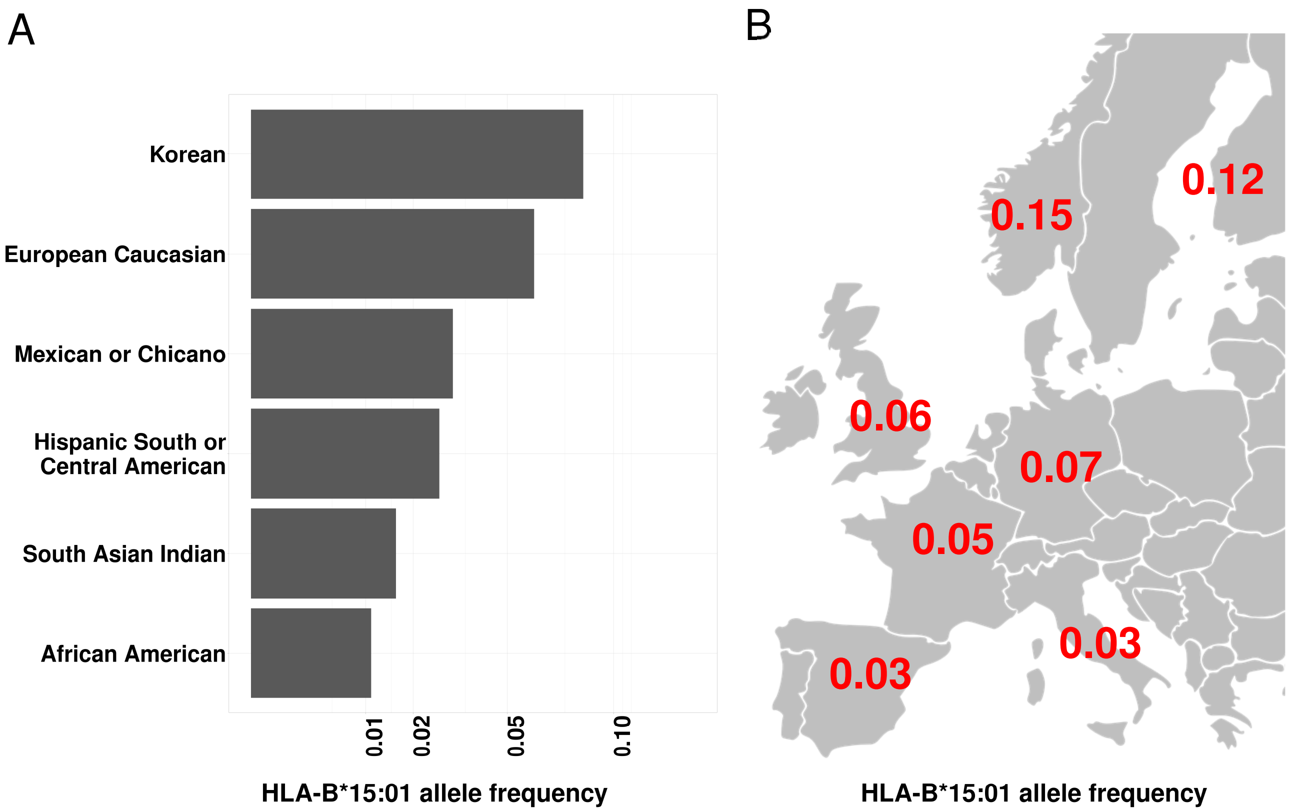

Figure S5: Frequency of *HLA-B*15:01* allele:
(A) for the various subpopulations in the US. Names are those used by the USA National Marrow Donor Program (NMDP). (B) for various countries in Europe. Data from Allele Frequency Net Database (www.allelefrequencies.net) and 1000 Genomes Project.

Figure S6

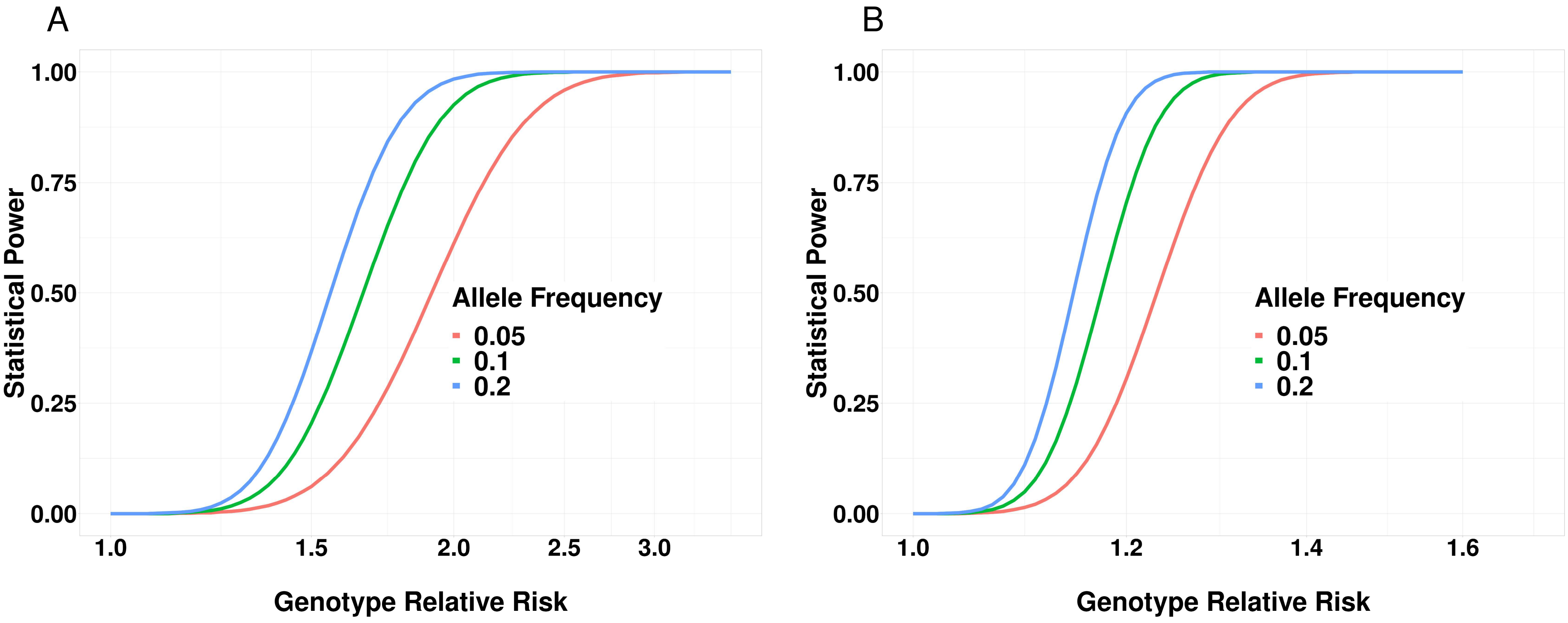

Figure S6: Power curves: HLA-WAS.
Statistical power as a function of genotype relative risk, calculated under a dominant inheritance model, with various allele frequencies (0.05, 0.1, 0.2), a prevalence of asymptomatic infection of 0.1, a p-value threshold of 0.0005. A: in a combined cohort of 400 asymptomatic cases and 3,000 symptomatic controls. B: in a hypothetical cohort of 4,000 asymptomatic cases and 30,000 symptomatic controls.
Curves plotted with the Genetic Association Study Power Calculator (https://csg.sph.umich.edu/abecasis/gas_power_calculator).

Supplemental methods

Ethics approval and consent to participate

US prospective cohort

All participants enrolled in the US prospective cohort provided written informed consent for participation and were recruited through protocols conforming to local ethics requirements. The Helix DNA Discovery Project was reviewed and approved by the Western Institutional Review Board. For the Healthy Nevada Project (HNP), the University of Nevada, Reno Institutional Review Board approved the study (project 956068-12). The procedures followed were in accordance with ethical standards, and appropriate informed consent was obtained.

CHGE cohort

All the participants enrolled in the CHGE cohort provided written informed consent for participation and were recruited through protocols conforming to local ethics requirements. For patients enrolled in the French COVID cohort (ClinicalTrials.gov NCT04262921), ethics approval was obtained from the Comité de Protection des Personnes Ile De France VI (ID RCB, 2020-A00256-33) or the Ethics Committee of Erasme Hospital (P2020/203). For participants enrolled in the COV-Contact study (ClinicalTrials.gov NCT04259892), ethics approval was obtained from the CPP IDF VI (ID RCB, 2020-A00280-39). For patients enrolled in the Italian cohort, ethics approval was obtained from the University of Milano-Bicocca School of Medicine, San Gerardo Hospital, Monza–Ethics Committee of the National Institute of Infectious Diseases Lazzaro Spallanzani (84/2020) (Italy), and the Comitato Etico Provinciale (NP 4000–Studio CORONAlab). STORM-Health care workers were enrolled in the STudio OsseRvazionale sullo screening dei lavoratori ospedalieri per COVID-19 (STORM-HCW) study, with approval from the local institutional review board (IRB) obtained on June 18, 2020. Patients and relatives from San Raffaele Hospital (Milan) were enrolled in COVID-BioB/Gene-COVID protocols and, for additional studies, TIGET-06, with the approval of the local ethics committee. Patients and relatives from Rome were enrolled in Protocol no. 50/20 (Tor Vergata University Hospital). Informed consent was obtained from each patient. For the patients enrolled in the COVIDeF Study Group (ClinicalTrials.gov NCT04352348), ethics approval was obtained from the Comité de Protection des Personnes Ile de France XI (ID RCB, 2020-A00754-35). For patients enrolled in Spain, the study was approved by the Committee for Ethical Research of the Infanta Leonor University Hospital, code 008–20; the Committee for Ethical Research of the 12 de Octubre University Hospital, code 16/368; the Bellvitge University Hospital, code PR127/20; the University Hospital of Gran Canaria Dr. Negrín, code 2020–200-1 COVID-19; and the Vall d'Hebron University Hospital, code PR(AMI)388/2016. Anonymized samples were sequenced at the National Institute of Allergy and Infectious Diseases (NIAID) through the Uniformed Services University of the Health Sciences (USUHS)/the American Genome Center (TAGC) under nonhuman subject research conditions; no additional IRB consent was required at the National Institutes of Health (NIH). For patients enrolled in the Swedish COVID cohort, ethics approval was obtained from the Swedish Ethical Review Agency (2020–01911 05).
