# Supplementary material for: Lack of association between classical HLA genes and asymptomatic SARS-CoV-2 infection
Source: HGG Adv. 2024 Apr 26;5(3):100300. doi: 10.1016/j.xhgg.2024.100300 (PMC11215417; doi:10.1016/j.xhgg.2024.100300)
Supplement: Document S2. Article plus supplemental information [file mmc4.pdf]

HGG Advances

ARTICLE

Lack of association between classical HLA genes and asymptomatic SARS-CoV-2 infection

Astrid Marchal,[1,2] Elizabeth T. Cirulli,[3] Iva Neveux,[4] Evangelos Bellos,[5] Ryan S. Thwaites,[6] Kelly M. Schiabor Barrett,[3] Yu Zhang,[7] Ivana Nemes-Bokun,[5] Mariya Kalinova,[8] Andrew Catchpole,[8] Stuart G. Tangye,[9,10] András N. Spaan,[11,12] Justin B. Lack,[13] Jade Ghosn,[14,15] Charles Burdet,[14,16,17] Guy Gorochov,[18] Florence Tubach,[19] Pierre Hausfater,[20,21] COVID Human Genetic Effort, COVIDeF Study Group, French COVID Cohort Study Group, CoV-Contact Cohort, COVID-STORM Clinicians, COVID Clinicians, Orchestra Working Group, Amsterdam UMC COVID-19 Biobank, NIAID-USUHS COVID Study Group,[30] Clifton L. Dalgard,[22] Shen-Ying Zhang,[1,2,11] Qian Zhang,[1,2,11] Christopher Chiu,[5] Jacques Fellay,[23,24,25] Joseph J. Grzymski,[4,26] Vanessa Sancho-Shimizu,[5,27] Laurent Abel,[1,2,11] Jean-Laurent Casanova,[1,2,11,28,29] Aurélie Cobat,[1,2,11,*] and Alexandre Bolze[3,31,*]

Summary

Human genetic studies of critical COVID-19 pneumonia have revealed the essential role of type I interferon-dependent innate immunity to SARS-CoV-2 infection. Conversely, an association between the *HLA-B*15:01* allele and asymptomatic SARS-CoV-2 infection in unvaccinated individuals was recently reported, suggesting a contribution of pre-existing T cell-dependent adaptive immunity. We report a lack of association of classical HLA alleles, including *HLA-B*15:01*, with pre-omicron asymptomatic SARS-CoV-2 infection in unvaccinated participants in a prospective population-based study in the United States (191 asymptomatic vs. 945 symptomatic COVID-19 cases). Moreover, we found no such association in the international COVID Human Genetic Effort cohort (206 asymptomatic vs. 574 mild or moderate COVID-19 cases and 1,625 severe or critical COVID-19 cases). Finally, in the Human Challenge Characterisation study, the three *HLA-B*15:01* individuals infected with SARS-CoV-2 developed symptoms. As with other acute primary infections studied, no classical HLA alleles favoring an asymptomatic course of SARS-CoV-2 infection were identified.

Introduction

Primary infection with SARS-CoV-2 underlies a broad spectrum of clinical manifestations in unvaccinated individuals, ranging from silent infection to lethal COVID-19 pneumonia. Rare and common human genetic variants have been associated with hypoxemic COVID-19 pneumonia.[1–6] Inborn errors of TLR3- and/or TLR7-dependent type I interferon (IFN) immunity underlie critical COVID-19 pneumonia in 1%–5% of cases.[1,7–9] Moreover, autoantibodies neutralizing type I IFN underlie at least another 15% of cases,[10–12] further highlighting the key role of type I IFNs in protective immunity to SARS-CoV-2 infection in the respiratory tract. While inborn errors are preferentially found in young patients with critical COVID-19 cases, the autoantibodies are more common in the elderly.[3] Common variants in or near genes involved in viral entry into respiratory cells or airway defense have also been associated with severe COVID-19.[5] In contrast, only a few associations between human leukocyte antigen (HLA) alleles and COVID-19 severity were consistently reported in the many studies that tested the hypothesis

[1]Laboratory of Human Genetics of Infectious Diseases, Necker Branch, INSERM U1163, Paris, France; [2]University Paris Cité, Imagine Institute, Paris, France; [3]Helix, San Mateo, CA, USA; [4]Department of Internal Medicine, University of Nevada School of Medicine, Reno, NV, USA; [5]Department of Infectious Disease, Imperial College London, London, UK; [6]National Heart and Lung Institute, Imperial College London, London, UK; [7]Laboratory of Clinical Immunology and Microbiology, Division of Intramural Research, NIAID, Bethesda, MD, USA; [8]hVIVO Services Ltd, London, UK; [9]Garvan Institute of Medical Research, Darlinghurst, NSW, Australia; [10]School of Clinical Medicine, Faculty of Medicine and Health, UNSW Sydney, New South Wales, Australia; [11]St. Giles Laboratory of Human Genetics of Infectious Diseases, Rockefeller Branch, The Rockefeller University, New York, NY, USA; [12]Department of Medical Microbiology, University Medical Center Utrecht, Utrecht University, Utrecht, the Netherlands; [13]NIAID Collaborative Bioinformatics Resource, Frederick National Laboratory for Cancer Research, Leidos Biomedical Research Inc, Frederick, MD, USA; [14]Infection, Antimicrobials, Modelling, Evolution (IAME), INSERM, UMR1137, University Paris Cité, Paris, France; [15]AP-HP, Bichat-Claude Bernard Hospital, Infectious and Tropical Diseases Department, Paris, France; [16]AP-HP, Hôpital Bichat, Centre d'Investigation Clinique, INSERM CIC 1425, Paris, France; [17]Département Epidémiologie, Biostatistiques et Recherche Clinique, Hôpital Bichat, Assistance Publique-Hôpitaux de Paris, 75018 Paris, France; [18]Sorbonne Université, INSERM Centre d'Immunologie et des Maladies Infectieuses, CIMI-Paris, Département d'immunologie Hôpital Pitié-Salpêtrière, Assistance Publique-Hôpitaux de Paris, Paris, France; [19]Sorbonne Université, INSERM, Institut Pierre Louis d'Epidémiologie et de Santé Publique, AP-HP, Hôpital Pitié-Salpêtrière, Département de Santé Publique, Unitéde Recherche Clinique PSL-CFX, CIC-1901, Paris, France; [20]Emergency Department, Hôpital Pitié-Salpêtrière, APHP-Sorbonne Université, Paris, France; [21]GRC-14 BIOSFAST Sorbonne Université, UMR INSERM 1135, CIMI, Sorbonne Université, Paris, France; [22]Department of Anatomy, Physiology & Genetics, Uniformed Services University of the Health Sciences, Bethesda, MD, USA; [23]School of Life Sciences, École Polytechnique Fédérale de Lausanne, Lausanne, Switzerland; [24]Swiss Institute of Bioinformatics, Lausanne, Switzerland; [25]Precision Medicine Unit, Lausanne University Hospital and University of Lausanne, Lausanne, Switzerland; [26]Renown Health, Reno, NV, USA; [27]Centre for Paediatrics and Child Health, Faculty of Medicine, Imperial College London, London, UK; [28]Department of Pediatrics, Necker Hospital for Sick Children, Paris, France; [29]Howard Hughes Medical Institute, New York, NY, USA
[30]Further details can be found in the supplemental information
[31]Lead contact
*Correspondence: aurelie.cobat@inserm.fr (A.C.), alexandre.bolze@gmail.com (A.B.)
https://doi.org/10.1016/j.xhgg.2024.100300.
© 2024 The Author(s). This is an open access article under the CC BY license (http://creativecommons.org/licenses/by/4.0/).

that HLA genes would be associated with COVID-19 because of their well-established role in T cell responses to viruses.[13,14] The COVID-19 Host Genetics Initiative analyzed more than 20,000 cases and 2,000,000 controls (data freeze 6) to identify their first association between an HLA allele and COVID-19 severity: *HLA-DRB1*04:01* conferred a small decrease in the risk of critical COVID-19 (odds ratio [OR] = 0.8).[2,5]

While human genetics proved fruitful to decipher some causes of severe COVID-19, very few studies investigated why a small percentage of unvaccinated adults presented with asymptomatic SARS-CoV-2 infection.[6] In this context, in July 2023, an association was reported between the *HLA-B*15:01* allele and asymptomatic SARS-CoV-2 infection in unvaccinated individuals.[15] The OR was 2.40 (95% confidence interval [CI]: 1.54–3.64) for heterozygotes, reaching 8.58 (95% CI: 1.74–34.43) in homozygotes. The authors replicated this association in a smaller independent cohort. This study further showed that T cells from HLA-B*15:01 individuals who had not been infected with SARS-CoV-2 recognized a SARS-CoV-2 T cell epitope by cross-reactivity due to prior exposure to one of two common cold coronaviruses: OC43-CoV or HKU1-CoV.[15] Moreover, more than 100 immunogenic SARS-CoV-2 peptides are highly similar to peptides from at least one human coronavirus (hCoV) presented by a wide range of classical HLA molecules.[16] We, therefore, tested the hypothesis of an association between HLA alleles and asymptomatic SARS-CoV-2 infection in two independent cohorts. We aimed (1) to test the association with *HLA-B*15:01* and (2) to identify additional HLA alleles potentially associated with asymptomatic COVID-19.

Material and methods

Cohorts and phenotype information

US prospective cohort

Participants in the US prospective cohort came from two studies: the Helix DNA Discovery Project and the Healthy Nevada Project. All enrolled participants provided written informed consent for participation and were recruited through protocols conforming to local ethics requirements. Participants were recruited before the start of the COVID-19 pandemic. We performed an online survey that we sent a few times in 2021. The survey takes about 15 min to complete and has been published in the past.[17] We received responses from 8,125 unique Helix DNA Discovery Project participants and 9,315 unique Healthy Nevada Project participants. The participants in this cohort were 18–89+ years old, 65% were female, and 85% were of European genetic similarity. The respondents indicated whether they had been infected and whether they had been vaccinated, as well as information on exposure, reasons for testing, and comorbidities. They rated the severity and duration of their symptoms and disease. They answered questions about 24 specific symptoms known to occur after SARS-CoV-2 infection.

This cohort was previously included in the large meta-analysis of the COVID-Host Genetic initiative genome-wide association study (GWAS) for susceptibility to infection. In the data freeze 7, 1,000 cases and 10,000 controls came from this cohort representing fewer than 1% of cases and 5% of total controls.[5] More recently, this cohort was used to identify the association between *HLA-A*03:01* and COVID-19 mRNA vaccine side effects.[17]

CHGE cohort

Since the beginning of the pandemic, the COVID Human Genetic Effort (CHGE) has enrolled more than 10,000 individuals with SARS-CoV-2 infection and broad clinical manifestations from all over the world. All the enrolled participants provided written informed consent for participation and were recruited through various protocols and cohorts, including the COVIDeF cohort, the French COVID cohort, the CoV-Contact Cohort, the Orchestra Working Group, the Amsterdam UMC COVID-19 Biobank, the NIAID-USUHS COVID study, and the COVID-STORM study (see supplemental methods), aiming at recruiting both hospitalized COVID-19 cases and infected controls. The physicians classified the patients according to World Health Organization (WHO) criteria,[18] as follows: (1) Critical cases were defined as hospitalized patients with pneumonia requiring high-flow oxygen (>6 L/min) and/or requiring admission to the intensive care unit (WHO score ≥6); (2) severe cases were defined as hospitalized patients with pneumonia requiring low-flow oxygen (WHO score = 5); (3) moderate cases were defined as hospitalized patients with pneumonia not requiring oxygen (WHO score = 4); (4) mild cases were defined as pauci-symptomatic ambulatory patients, with the presence of mild, self-healing symptoms such as cough, fever, body aches, or anosmia (WHO score = 2 or 3); and (5) asymptomatic cases were defined as infected individuals with no symptoms, having a score of 1 in the WHO clinical progression scale.[18] The presence of infection was assessed on the basis of a positive PCR test and/or serological test and/or the presence of typical symptoms such as anosmia or ageusia after exposure to a confirmed COVID-19 case.

Some of the samples of this cohort were previously included in studies focusing on the genetic causes of life-threatening COVID-19 pneumonia.[1,7,9] The largest overlap is with the genome-wide burden screen of rare coding variants associated with critical COVID-19 pneumonia published in 2023 in *Genome Medicine*,[7] in which most of the critical patients of the present study were included as cases, and most of the asymptomatic and mild individuals were included as controls. By contrast, most of the moderate and severe patients have not been analyzed so far. HLA association has never been previously investigated in any of the samples of the CHGE dataset.

SARS-CoV-2 Human Challenge Characterisation Study

Thirty-six participants were recruited, two became seropositive to spike protein prior to study start. The 34 seronegative participants were challenged with D614G-containing pre-Alpha SARS-CoV-2, of whom 33 consented for genetic analysis and were analyzed in the present study. Additional details on the study design and participants were previously published.[19,20] Ethics approval was obtained from the UK Health Research Authority Ad Hoc Specialist Ethics Committee (reference 20/UK/0002). Written informed consent was obtained from participants before screening and enrollment.

Sequencing

US prospective cohort

DNA samples were sequenced and analyzed at Helix with the Exome+ assay, which targets the exome and a few hundred

thousand non-exonic common SNPs, providing a backbone for imputation of the most common SNPs in the genome as previously described.[21] Genotype processing was performed in Hail.[22]

For each individual, we ran a supervised ADMIXTURE[23] algorithm with k = 5 populations using the 1000 Genomes dataset. From these admixture coefficients, we then labeled each individual with one genetic similarity using the following decision tree:

- If (ADMIX_EUR>0.85) & (ADMIX_AFR<0.1) & (ADMIX_AMR<0.1) & (ADMIX_EAS<0.1) & (ADMIX_SAS<0.1) then "Europe"
- Else If ADMIX_EAS>0.6 then "East Asia"
- Else If ADMIX_SAS>0.6 then "South Asia"
- Else If (ADMIX_AFR >0.3) & (ADMIX_EAS<0.1) & (ADMIX_SAS<0.1) & (ADMIX_AFR > ADMIX_AMR) then "Africa"
- Else If (ADMIX_AMR>0.1) & (ADMIX_EAS<0.1) & (ADMIX_SAS<0.1) then "Americas"
- Else is "Other"

Principal-component analysis (PCA) was done using a set of 184,445 coding and noncoding LD-pruned, high-quality common variants. Eigenvalues and scores were calculated using the hwe_normalized_pca function in Hail.

CHGE cohort

Whole-exome (WES) or whole-genome sequencing (WGS) was performed at several sequencing centers, including the Genomics Core Facility of the Imagine Institute (Paris, France), the Yale Center for Genome Analysis (USA), Macrogen (USA), Psomagen (USA), the New York Genome Center (NY, USA), the American Genome Center (USUHS, Bethesda, MD, USA), MNM Bioscience (Poland), Invitae (San Francisco, CA, USA), the Genomic Sequencing Platform Seqoia (France), the Centre National de Recherche en Génomique Humaine (CNRGH, Evry, France), the Genomics Division-ITER of the Canarian Health System sequencing hub (Canary Islands, Spain), and the AlJalila Genomics Center (Dubai). Libraries for WES were generated with the Twist and Twist Plus Human Core Exome Kit, the xGen Exome Research Panel from Integrated DNA Technologies (IDT; xGen V1 and V2), Agilent SureSelect (Human All Exon V6 and V7) panels, the SeqCap EZ MedExome Kit from Roche, the Nextera Flex for Enrichment-Exome kit, the Illumina TruSeq Exome panel, and WES custom target enrichment probes. Massively parallel sequencing was performed on HiSeq 4000, HiSeq 2500, NextSeq 550, or NovaSeq 6000 systems (Illumina).

For PCA, common variants from the gnomAD v2.1 Exome dataset were jointly genotyped with GATK GenotypeGVCFs. PCA was performed with PLINK v1.9 software on a pruned set of ~14,600 SNPs not in linkage disequilibrium (maximum r^2 value for linkage disequilibrium of 0.4 between pairs of SNPs), with a minor allele frequency (MAF) >1%, a call rate >99%, and *p* value for departure from Hardy-Weinberg equilibrium $>10^{-5}$, as previously described.[24] The ancestral origin of the patients was further inferred from the PCA, as previously described.[24]

SARS-CoV-2 Human Challenge Characterisation Study

WGS was performed on Illumina NovaSeq (Novogene Ltd., UK), yielding 150 bp paired-end reads. The average depth of coverage was >50x with a minimum of 31x. PCA and global ancestry inference were performed using Hail according to the protocol described by the gnomAD project.[25]

HLA calls/imputation

US prospective cohort

HLA alleles were imputed for seven genes: *HLA-A, -B, -C, -DPB1, -DQA1, -DQB1*, and *-DRB1* with HIBAG using the default recommendations.[26] Individual genotypes were imputed with the model that was the most appropriate based on the genetic similarity for each individual. Specifically, we used the African model for individuals in the Africa genetic similarity group, the Asian model for those in the East Asia or the South Asia genetic similarity groups, the European model for those in the Europe or Other genetic similarity groups, and the Hispanic model for those in the Americas genetic similarity group. Models were downloaded from https://hibag.s3.amazonaws.com/hlares_index.html. As an example, the European model was the HLA4-hg19.RData model (available: https://hibag.s3.amazonaws.com/download/hlares_param/European-HLA4.html). Probabilities greater than 0.5 were used for genotype calling. All alleles had *p* value for departure from Hardy-Weinberg equilibrium $>10^{-4}$.

CHGE cohort

Classical class I and class II HLA alleles were typed from the raw WES or WGS reads with HLA*LA software,[27] which uses a linear projection method to align reads to a population reference graph and enables high HLA typing accuracy from WES or WGS data. Alleles with *p* value for departure from Hardy-Weinberg equilibrium $<10^{-4}$ were excluded.

SARS-CoV-2 Human Challenge Characterisation Study

HLA alleles were typed from raw WGS reads with HLA*LA software at G group resolution. Only HLA calls with a posterior probability of 100% and a minimum coverage of 20x were retained in the analysis. At the B locus, all individual calls fulfilled these filtering criteria at 2-field resolution.

These tools have been validated for their accuracy to call HLA alleles at 2-field resolution, particularly in populations of European ancestry.[26,28–30] For example, the HIBAG HLA calls made at Helix for seven genes in seven European ancestry Coriell samples showed 99% concordance with the known HLA calls for these individuals. Inferred HLA alleles between HIBAG and HLA*LA were more than 96% identical at four-digit resolution in the COVID-HGI study.[2] Differences caused by HLA allele calling should mostly be limited to rare HLA types and populations with poor imputation references.

HLA-WAS

We used Regenie[31] for the genetic analysis. In brief, this method builds a whole-genome regression model based on common variants to account for the effects of relatedness and population stratification; it also accounts for situations in which there is an extreme case-control imbalance likely to lead to test statistic inflation with other analysis methods. We used the approximate Firth *p* value when the logistic regression *p* value was below 0.01. The covariates included were age group, sex, and the first five principal components, as recommended to accurately correct for population structure.[31] We used a Bonferroni correction for multiple testing.

For the US prospective cohort, a representative set of 184,445 coding and noncoding LD-pruned, high-quality common variants were identified for the construction of PCs and the whole-genome regression model, as previously described.[21] PCs were calculated within the European group. For CHGE, the set of ~14,600 SNPs used for PCA within the European group was used for the whole-genome regression model.

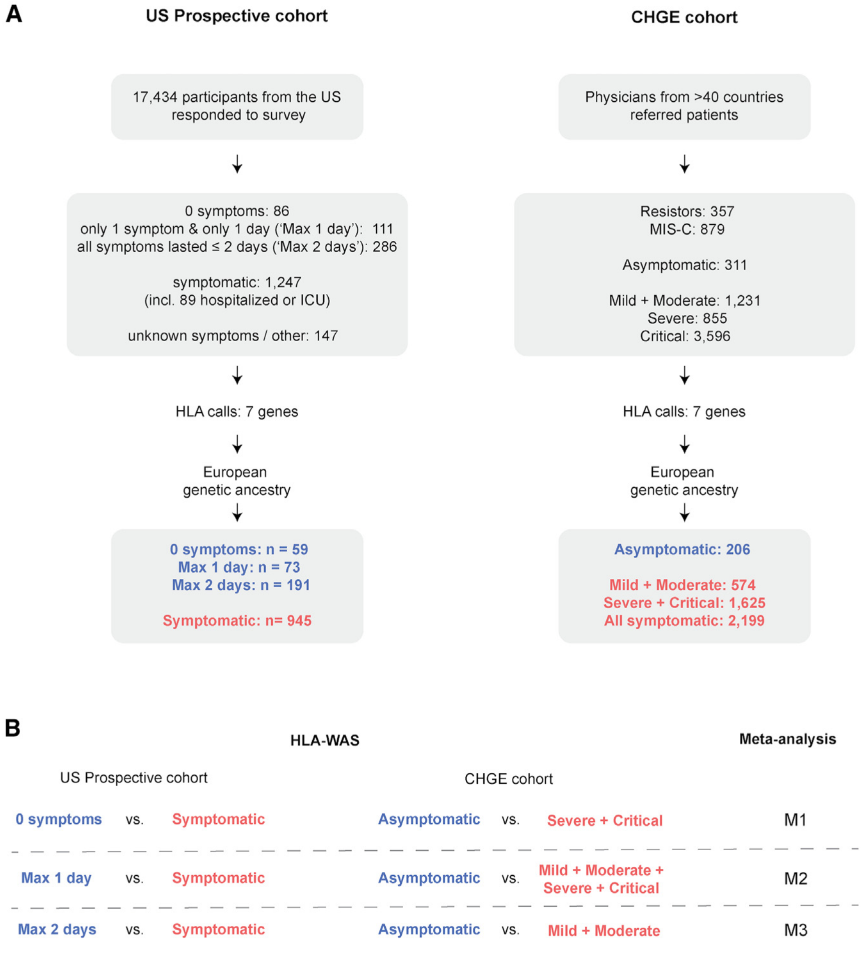

Figure 1. Study design
(A) Description of the two cohorts and definitions of asymptomatic and symptomatic cases. ICU, intensive care unit.
(B) List of HLA-wide association studies and meta-analyses performed.

Meta-analysis

Results were combined by inverse variance-weighted fixed-effects meta-analysis with METAL.[32] Effect was provided as the BETA value and the STDERR was provided as the SE.

Power calculation

We estimated the power required to detect an effect similar to that reported by Augusto et al. with the Genetic Association Study (GAS) Power Calculator, which uses a method derived from the CaTS power calculator for two-stage association studies.[33] Power was first estimated as a function of the OR for the specific replication of the *HLA-B*15:01* association using the following parameters: allele frequency of 0.05; prevalence of asymptomatic infection: 0.1; Dominant inheritance model; type I error of 0.05; numbers of cases and controls according to the third definition in both cohorts. In a second step we estimated the power to detect an association in the context of an HLA-wide screen (i.e., using a type I error of 5×10^{-4}) as a function of the OR and for various allele frequencies using a sample size either corresponding to our combined samples (400 cases and 3,000 controls) or a sample size which would be 10 times larger (4,000 cases and 30,000 controls).

Serology

Plasma immunoglobulin (Ig)G titers for the SARS-CoV-2 Human Challenge Characterisation Study participants were determined using MesoScale Discovery Coronavirus panel 2 plates on an SQ120 instrument. Binding titers given as arbitrary units per milliliter (AU/mL) based on a kit-provided human plasma standard curve.

Results

HLA-wide association in the US prospective cohort

We first conducted an HLA-wide association study (HLA-WAS) in a prospective population-based US cohort (Figure 1). The 17,434 adults who responded to at least one of the COVID-19 infection and vaccination surveys sent in 2021 included 1,680 participants reporting SARS-CoV-2 infection while unvaccinated. A continuous spectrum of symptoms, duration of illness was reported following SARS-CoV-2 infection (Figure S1). The most common symptoms were muscle and body aches, and a cough (Figure S1A). No symptoms at all were reported by 5.1% of individuals ($n = 86$), whereas 5.3% of the infected participants required hospitalization with or without oxygen therapy ($n = 58$) or were admitted to the intensive care unit ($n = 31$) (Figure S1B). We tested the hypothesis that HLA alleles play an important role in the early response to SARS-CoV-2 by considering three case definitions for the asymptomatic cases (Figure 1A): (1) “0 symptoms” was a stringent definition of asymptomatic as a total absence of symptoms ($n = 86$); (2) “Max 1 day” was a definition of asymptomatic in which the presence of one symptom for no more than 1 day was tolerated ($n = 111$). This definition was used to increase the power for detection of an association by enlarging the “asymptomatic” group while still identifying individuals who cleared the virus quickly and efficiently; (3) “Max 2 days” was a definition as close as possible to that used by Augusto et al., considering participants to be asymptomatic if none of their symptoms lasted 3 days or more, and if the reason for testing was unrelated to symptoms ($n = 286$).[15] We used only one definition for controls (individuals with symptoms lasting at least 3 days). The control group included all individuals admitted to the intensive care unit or the hospital and anyone reporting symptoms of at least 3 days' duration with some impact on their daily routine ($n = 1{,}247$). For the HLA-WAS, we restricted the analysis to individuals

Table 1. Top-ranked alleles in the HLA-WAS on the US prospective cohort

Allele	OR [95% CI][a]	Raw p value[b]	AF[c]	Asymptomatic definition[d]
DRB1*16:01	0.06 [0.002–1.5]	0.004	0.012	Max 2 days
A*32:01	2.19 [1.3–3.8]	0.008	0.035	Max 2 days
B*07:02	1.58 [1.1–2.3]	0.016	0.13	Max 2 days
C*07:02	1.53 [1.1–2.2]	0.020	0.14	Max 2 days
DQB1*06:02	1.52 [1.1–2.2]	0.025	0.14	Max 2 days
DQB1*05:02	0.29 [0.1–1.1]	0.037	0.015	Max 2 days

[a]Odds ratio (OR) of being asymptomatic, i.e., OR >1 indicates that the allele is more frequent in asymptomatic individuals. CI = confidence interval.
[b]Significance threshold after Bonferroni correction for multiple testing is 0.00048. Alleles shown are those with a raw p value <0.05.
[c]Allele frequency (AF) is based on the frequency of the allele in the US prospective cohort "Max 2 days" analysis because this analysis included the largest numbers of cases and controls.
[d]For each allele, the top-ranked result across the three asymptomatic definitions in the US prospective cohort is given.

who were genetically similar to a reference group from Europe often denoted as individuals of "European ancestry" (Figures S1C and S1D), leading to a total of 59 to 191 asymptomatic cases and of 945 symptomatic controls (Figure 1A). Age and sex distribution are shown in Table S1. The association test was performed with Regenie[31] under a dominant inheritance model, with age, sex, and the first five principal components as covariates (see supplemental methods). The risk of detecting false-positive associations was decreased by limiting the analysis to the 105 HLA alleles with an allele frequency of at least 1% in this cohort. No statistically significant associations (at a corrected threshold of $p < 0.00048$) were found with any of the three phenotype definitions (Tables 1 and S2–S4). The top-ranked HLA allele was *DRB1*16:01*, which was depleted in asymptomatic individuals, with the strongest effect being observed in the "Max 2 days" group of asymptomatic patients (OR [95% CI] = 0.06 [0–1.5], $p = 0.004$, $p_{\text{corrected}} = 0.42$, Table 1).

HLA-wide association in the CHGE cohort

We next studied patients recruited by the physicians of the international CHGE consortium. These physicians classified participants with SARS-CoV-2 infections according to acute disease severity: asymptomatic, mild, moderate, severe, or critical (Figure S2A). Whole-exome or WGS data were available for 7,229 participants and HLA alleles were typed with HLA*LA.[27] In this HLA-WAS, we compared the patients classified as "asymptomatic" by the clinicians ($n = 311$) with those in three sets of symptomatic controls: (1) the patients with the most extreme symptoms requiring hospitalization and oxygen supplementation (i.e., those with a severe or critical form of the disease, $n = 4{,}451$); (2) all symptomatic patients, whatever their acute disease severity (i.e., mild, moderate, severe or critical, $n = 5{,}682$); and (3) symptomatic patients not requiring oxygen supplementation (i.e., mild and moderate patients only, $n = 1{,}231$); this last group of symptomatic patients is the most similar to the symptomatic patients groups of the US prospective cohort and the study by Augusto et al. (Figure 1). We restricted the analysis to individuals of European genetic ancestry and the final study population comprised 206 asymptomatic cases, 1,625 patients with severe or critical disease, and 574 patients with mild or moderate disease (Figures S2B and S2C). Age and sex distribution are shown in Table S1. Analyses were also performed under a dominant inheritance model with age group, sex, and the first five principal components as covariates. This analysis was performed with Regenie and was limited to the 114 HLA alleles with an allele frequency of at least 1% in this cohort. No statistically significant association (at a corrected threshold of $p < 0.00044$) was identified in the HLA-WAS, regardless of the definition of symptomatic patients used (Tables S5–S7). The top-ranked HLA allele found to be enriched in asymptomatic individuals was *HLA-B*40:02*, for which the strongest effect was observed in comparison with the group of symptomatic patients with severe or critical disease (OR [95% CI] = 3.4 [1.5–7.7], $p = 0.005$; $p_{\text{corrected}} = 0.57$, Table 2).

HLA-wide meta-analysis

We then performed three meta-analyses, denoted M1, M2, and M3 (Figure 1B), combining the results from our two independent cohorts with METAL.[32] The first used the strictest definitions for the groups: the HLA-WAS with the "0 symptoms" group of asymptomatic patients in the US prospective cohort and the HLA-WAS limited to patients with severe and critical disease only in the CHGE cohort (Table S8). The second meta-analysis combined the HLA-WAS with the "Max 1 day" definition of asymptomatic patients for the US prospective cohort (0 symptoms or one symptom for 1 day) with the HLA-WAS with all symptomatic cases from the CHGE (Table S9). The final meta-analysis used the results for the asymptomatic and symptomatic groups most closely resembling those of the study by Augusto et al. (Table S10). The meta-analyses detected no statistically significant associations (at a corrected threshold of $p < 0.00053$, 95 alleles tested) between HLA alleles and asymptomatic SARS-CoV-2 infection (Tables 3 and S8–S10). The top-ranked HLA allele was *HLA-B*40:02* (p value = 0.0008, $p_{\text{corrected}} = 0.095$), for which enrichment was observed in asymptomatic individuals relative to symptomatic individuals in both cohorts and in the

Table 2. Top-ranked alleles in the HLA-WAS on the CHGE European cohort

Allele	OR [95% CI][a]	Raw p value[b]	AF[c]	Controls used[d]
B*40:02	3.42 [1.5–7.7]	0.005	0.016	Severe + Critical
DPB1*01:01	0.28 [0.1–0.8]	0.007	0.042	Severe + Critical
A*23:01	2.5 [1.2–5.0]	0.010	0.023	Mild + Moderate
B*49:01	2.26 [1.2–4.3]	0.014	0.031	Mild + Moderate
A*03:01	1.66 [1.1–2.5]	0.019	0.12	Severe + Critical
DQA1*01:02	1.54 [1.1–2.2]	0.022	0.18	Mild + Moderate
A*30:02	2.46 [1.1–5.6]	0.031	0.019	Mild + Moderate
B*57:01	0.47 [0.2–1.0]	0.047	0.027	Mild + Moderate
A*68:02	3.53 [1.0–12.2]	0.047	0.01	Mild + Moderate
DPB1*03:01	0.65 [0.4–1.0]	0.049	0.093	Mild + Moderate

[a]Odds ratio (OR) of being asymptomatic, i.e., OR >1 indicates that the allele is more frequent in asymptomatic individuals. CI = confidence interval.
[b]Significance threshold after Bonferroni correction for multiple testing is 0.00044. Alleles shown are those with a raw p value <0.05.
[c]Allele frequency (AF) is based on the frequency of the allele in the CHGE European cohort "All" analysis, which included the largest numbers of cases and controls.
[d]For each allele, the top-ranked result across three sets of symptomatic patients in the CHGE European sample is given.

meta-analysis based on the strictest definitions. Of note, this allele has exactly the same frequency in cases and controls in the study of Augusto et al.[15]

Lack of replication for HLA-B*15:01

An analysis focusing on *HLA-B*15:01* did not replicate the association between *HLA-B*15:01* and asymptomatic SARS-CoV-2 infection (Figures 2A and 2B; Table 4) despite being well powered (>95%) to detect an effect similar to that reported by Augusto et al. (OR of 2.40 for enrichment in asymptomatic vs. symptomatic patients, $p = 5.67 \times 10^{-5}$) (Figure S3). We further estimated the frequency of *HLA-B*15:01* in various groups of patients of the CHGE consortium, including children with SARS-CoV-2 infection complicated by multisystem inflammatory syndrome (classified as MIS-C) and individuals with high levels of exposure who never tested positive (classified as "resistors").[34,35] This frequency ranged from 2.4% in asymptomatic individuals to 6.0% in resistors (Figure 2C). We also looked at individuals from non-European genetic ancestries. Similarly, we found no difference in frequency between asymptomatic and symptomatic individuals (Figure 2D and Table S11). Overall, no enrichment in the HLA-B*15:01 allele was observed among asymptomatic individuals in our US population-based prospective cohort, or in the international CHGE cohort.

Symptoms and serology for participants with HLA-B*15:01 in the SARS-CoV-2 Human Challenge Characterisation Study

The mechanism proposed as an explanation for the association between *HLA-B*15:01* and asymptomatic SARS-CoV-2 infection was pre-existing immunity, probably due

Table 3. Top-ranked alleles in the meta-analyses and corresponding results in the US prospective and CHGE cohorts

	Meta-analysis				US prospective cohort[c]		CHGE cohort[c]	
Allele	Meta-analysis number	OR [95% CI][a]	Raw p value[b]	Corrected p value	OR [95% CI][a]	Raw p value	OR [95% CI][a]	Raw p value
B*40:02	M1	3.51 [1.7–7.3]	0.0008	0.095	4.05 [0.7–24.6]	0.128	3.42 [1.5–7.7]	0.005
DPB1*01:01	M2	0.43 [0.3–0.7]	0.0015	0.17	0.43 [0.2–1.0]	0.058	0.43 [0.2–0.8]	0.010
DQA1*01:02	M3	1.41 [1.1–1.8]	0.007	0.80	1.31 [0.9–1.8]	0.119	1.54 [1.1–2.2]	0.022
A*23:01	M2	2.22 [1.2–4.0]	0.007	0.82	2.14 [0.7–6.6]	0.186	2.25 [1.1–4.4]	0.019
DQB1*06:02	M3	1.46 [1.1–2.0]	0.013	1	1.52 [1.1–2.2]	0.025	1.33 [0.8–2.2]	0.276
C*03:03	M1	0.52 [0.3–0.9]	0.015	1	0.48 [0.2–1.2]	0.132	0.54 [0.3–1.0]	0.055
B*49:01	M3	1.96 [1.1–3.4]	0.019	1	1.29 [0.4–3.9]	0.657	2.26 [1.2–4.3]	0.014
B*07:02	M3	1.42 [1.1–1.9]	0.021	1	1.58 [1.1–2.3]	0.016	1.16 [0.7–1.9]	0.554
DRB1*15:01	M3	1.38 [1.0–1.9]	0.037	1	1.42 [1–2.1]	0.075	1.32 [0.8–2.2]	0.264

[a]Odds ratio (OR) of being asymptomatic, i.e., OR>1 indicates that the allele is more frequent in asymptomatic individuals. CI = confidence interval.
[b]Significance threshold after Bonferroni correction for multiple testing is 0.00053. Alleles shown are those with a raw p value <0.05.
[c]For each allele, ORs and p values obtained in the US prospective and CHGE cohorts with asymptomatic or controls definitions used in the corresponding meta-analysis are given.

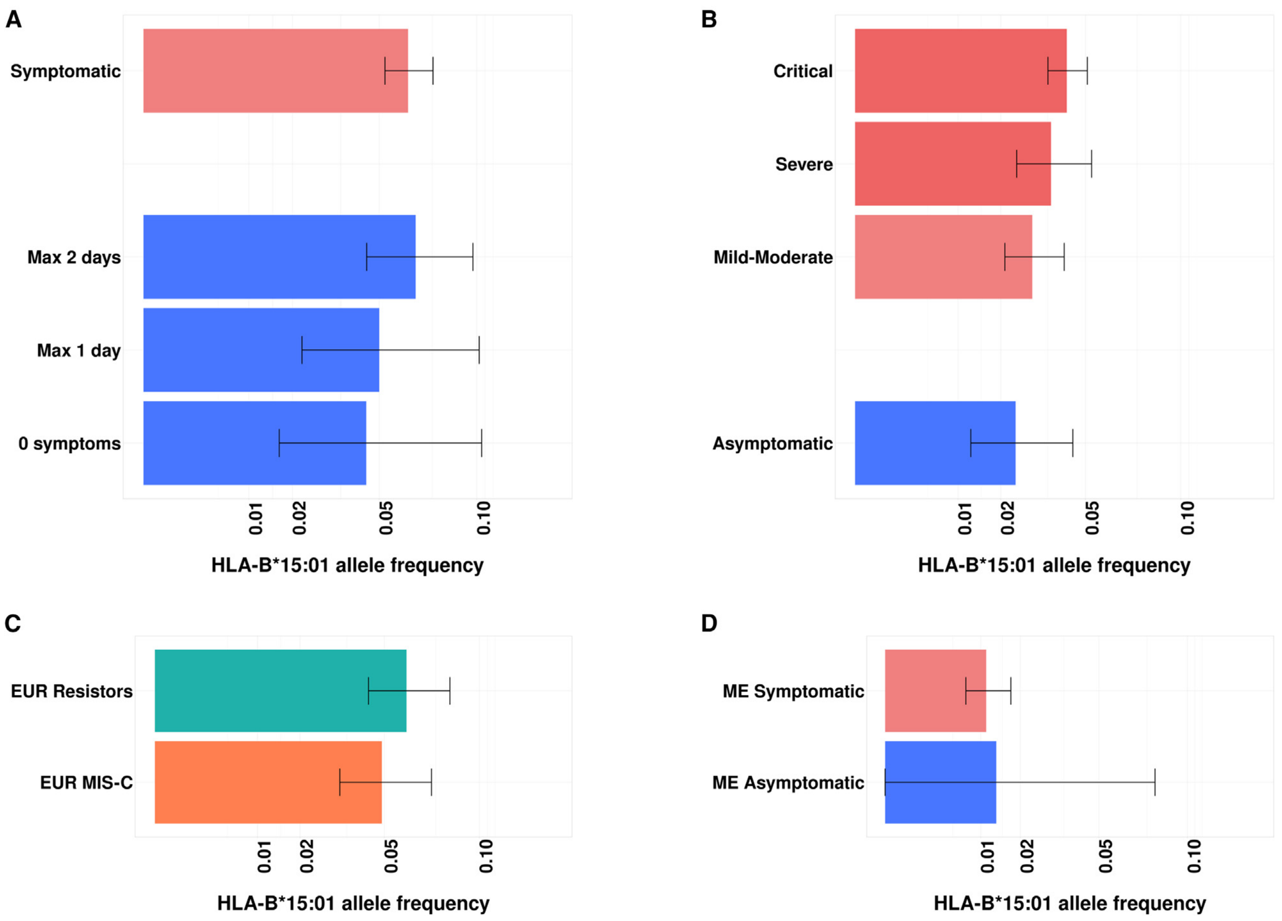

Figure 2. *HLA-B*15:01* is not enriched in asymptomatic cases
(A) Allele frequency and 95% confidence intervals in the US prospective cohort European subgroups.
(B) Allele frequency and 95% CIs in the CHGE European sample.
(C) Allele frequency and 95% CIs in individuals highly exposed to SARS-CoV-2 who never tested positive ("Resistors," $n = 291$) and in children with SARS-CoV-2 infection complicated by multisystem inflammatory syndrome ("MIS-C," $n = 235$) from the European CHGE sample.
(D) Allele frequency and 95% CIs in Middle Eastern (ME) individuals from the CHGE cohort (Symptomatic, $n = 895$; Asymptomatic, $n = 37$).

to prior infection with OC43-CoV or HKU1-CoV.[15] Unfortunately, no serological data were available for the HLA-B*15:01 carriers in the US prospective and the CHGE cohorts. We tested the hypothesis that the lack of association in our study was due to an absence of prior infection with OC43-CoV or HKU1-CoV by examining the data for the SARS-CoV-2 Human Challenge Characterisation Study (ClinicalTrials.gov identifier NCT04865237; funder, UK Vaccine Taskforce), in which 34 participants seronegative to spike protein were challenged with D614G-containing pre-Alpha SARS-CoV-2, of whom 33 consented for genetic analysis.[19] Serological data, history of prior infections with other coronaviruses, and genetic data were available, together with infection status and data concerning the recorded symptoms. HLA alleles were called with HLA*LA from whole-genome sequences obtained from the participants. Three of the 17 infected participants (positive test result) carried an *HLA-B*15:01* allele, as well as three of the 16 who stayed uninfected. Only one of the 17 infected participants was fully asymptomatic and this participant did not carry the *HLA-B*15:01* allele. The three infected participants with an *HLA-B*15:01* allele were symptomatic (Figure 3), despite evidence of prior exposure to OC43-CoV and HKU1-CoV (Figure S4). Thus, prior exposure to a coronavirus did not prevent the *HLA-B*15:01* carriers from developing symptoms following SARS-CoV-2 infection.

Discussion

Our analyses identified no associations between classical HLA alleles and asymptomatic SARS-CoV-2 infection. In particular, we did not replicate the previously reported association between *HLA-B*15:01* and asymptomatic SARS-CoV-2 infection, despite being well powered (>95%) for this specific replication study. Another recent study in a Spanish cohort found no associations between classical HLA alleles and asymptomatic SARS-CoV-2 infection.[36]

Table 4. **Odds ratio for the association of *HLA-B*15:01* with asymptomatic SARS-CoV-2 infection in both cohorts and in the meta-analysis**

Analysis	AF asymptomatic cases[a]	AF symptomatic controls[a]	OR [95% CI][b]	Raw *p* value
US prospective cohort	0.067	0.063	0.96 [0.58–1.6]	0.89
CHGE cohort	0.024	0.030	0.74 [0.36–1.53]	0.42
Meta-analysis M3	–	–	0.88 [0.58–1.34]	0.56

[a]Allele frequencies (AF) are based on the frequency of the allele in the US prospective cohort "Max 2 days" analysis and in in the CHGE European cohort "Mild + Moderate" analysis, combined in the meta-analysis M3.
[b]Odds ratio (OR) of being asymptomatic, i.e., OR >1 indicates that the allele is more frequent in asymptomatic individuals. CI = confidence interval.

One possible explanation for the difference in results regarding *HLA-B*15:01* is that the studies analyzed different groups of individuals living in different environments. However, the US prospective cohort we analyzed has many features in common with the cohort analyzed by Augusto et al.: specifically, the participants were from the United States, with a slight bias toward women, and the phenotype was assessed on the basis of self-reported surveys at multiple time points during the pandemic before summer 2021 (before the SARS-CoV-2 Delta variant became dominant in the United States[37]). The percentage of individuals self-reporting asymptomatic infection was similar between the two, as were the rates of each symptom. Alternatively, the difference in results may reflect differences in the handling of potential population stratification. Augusto et al. did not consider population structure in their study on bone marrow donors, probably because no genetic information outside of the HLA region was available, whereas we accounted for population structure by restricting our analysis to those of European ancestry and including the first five principal components as covariates in our regression model. The highly polymorphic nature of the HLA region and the differences in allele frequencies between human sub-populations contribute to a high risk of false-positive results in association analyses. The frequency of *HLA-B*15:01* is known to vary across continents, between continental populations within the United States (Figure S5A), and even between European countries (Figure S5B). Augusto et al. used self-identified race (White) to select their participants; however, this is not an appropriate proxy of genetic ancestry.[38] Population stratification may, thus, have played a confounding role in their study.

Overall, the absence of an association between classical HLA alleles and asymptomatic SARS-CoV-2 infection is consistent with the modest impact of HLA variation on severe or critical COVID-19.[5,14,39] A limitation of our study is the limited power to detect an association in the context of an HLA-wide screen, in particular due to the relatively small number of asymptomatic individuals. Using a sample size combining our two cohorts, we had an 80% power to detect an association with an allele with a minor allele frequency (MAF) of 0.05 and an OR of 2.18, or an allele with a MAF of 0.2 and an OR of 1.72 (Figure S6). With a sample size 10X bigger, we would have an 80% power to detect an association leading to an OR of 1.29 and 1.18 for an allele with a MAF of 0.05 and 0.2, respectively (Figure S6). Nonetheless, our results indicate that a potential association between an HLA allele and asymptomatic SARS-CoV-2 infection would not have a strong effect. This result is also consistent with the absence of any strong association between HLA alleles and clinical outcomes during the acute phase for the other primary viral infections studied to date.[40–42] By contrast, HLA alleles have been associated with multiple clinical or laboratory outcomes during chronic infections, including viral (e.g., HIV, HBV, HCV), mycobacterial (e.g., leprosy), and protozoan infections.[42–45] HLA alleles are also known to be associated with responses to vaccinations,[46,47] including against COVID-19.[17,48] While pre-existing immunity due to prior infections with common cold coronaviruses might help preventing the development of clinical manifestations following SARS-CoV-2 infection, our results suggest that such pre-existing immunity would not strongly depend on HLA alleles.

Data and code availability

Data supporting the findings of this study are available within the manuscript and supplemental files. The WGS data of anonymized patients recruited through the National Institutes of Health (NIH) and sequenced at the National Institute of Allergy and Infectious Diseases (NIAID) through the Uniformed Services University of the Health Sciences (USUHS)/the American Genome Center (TAGC) are available under dbGaP submission phs002245.v1. Other patients were not consented to share the raw WES/WGS data files beyond the research and clinical teams.

Supplemental information

Supplemental information can be found online at https://doi.org/10.1016/j.xhgg.2024.100300.

Acknowledgments

Funding was provided to the Desert Research Institute (DRI) by the Nevada Governor's Office of Economic Development. Funding was provided by Renown Health and the Renown Health Foundation.

The Laboratory of Human Genetics of Infectious Diseases is supported by the Howard Hughes Medical Institute, the Rockefeller University, the St. Giles Foundation, the National Institutes of Health (NIH) (R01AI63029), the National Center for Advancing

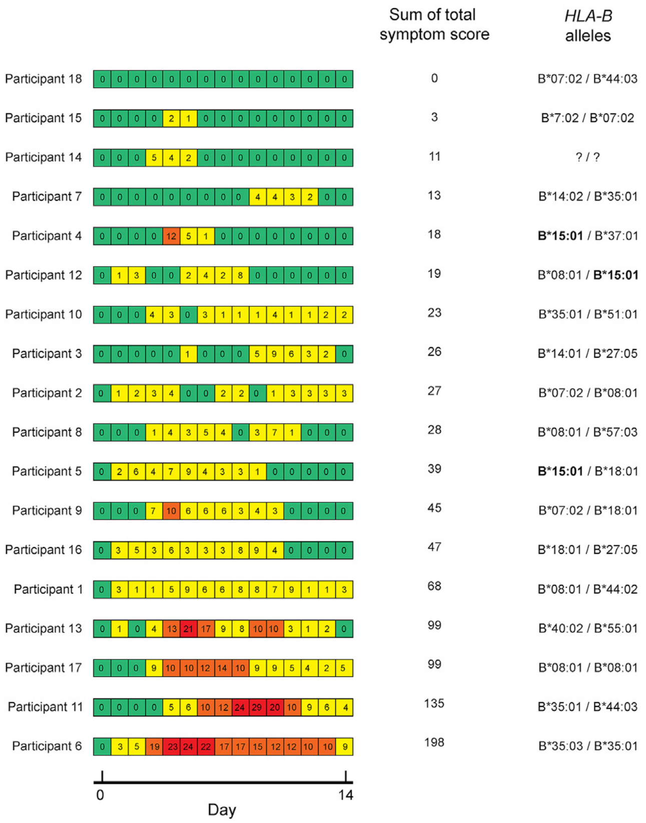

Figure 3. *HLA-B*15:01* in the SARS-CoV-2 Human Challenge Characterisation Study: Symptoms and *HLA-B* genotypes for 18 infected participants
Daily total symptom score was calculated using self-reported symptom diaries three times daily. Daily total symptom scores are displayed in the heatmap, ranging from green (no symptoms) to red (highest symptom score). The heatmap is derived from Figure 2 in Zhou J. et al., *Lancet Microbe* (2023).[19]

Translational Sciences (NCATS), NIH Clinical and Translational Science Award (CTSA) program (UL1 TR001866), the Yale Center for Mendelian Genomics and the GSP Coordinating Center funded by the National Human Genome Research Institute (NHGRI) (UM1HG006504 and U24HG008956), the Yale High Performance Computing Center (S10OD018521), the Fisher Center for Alzheimer's Research Foundation, the JPB Foundation, the Meyer Foundation, the French National Research Agency (ANR) under the "Investments for the Future" program (ANR-10-IAHU-01), the Integrative Biology of Emerging Infectious Diseases Laboratory of Excellence (ANR-10-LABX-62-IBEID), the French Foundation for Medical Research (FRM) (Equation 201903007798), the ANRS-COV05, ANR GENVIR (ANR-20-CE93-003), and ANR AI2D (ANR-22-CE15-0046) projects, the ANR-RHU program (ANR-21-RHUS-08), the European Union's Horizon 2020 research and innovation program under grant agreement No. 824110 (EASI-genomics), the HORIZON-HLTH-2021-DISEASE-04 program under grant agreement 101057100 (UNDINE), the Square Foundation, Grandir - Fonds de solidarité pour l'enfance, Fondation du Souffle, the SCOR Corporate Foundation for Science, *William E. Ford, General Atlantic's Chairman and Chief Executive Officer, Gabriel Caillaux, General Atlantic's Co-President, Managing Director and Head of business in EMEA, and the General Atlantic Foundation*, the Battersea & Bowery Advisory Group, The French Ministry of Higher Education, Research, and Innovation (MESRI-COVID-19), Institut National de la Santé et de la Recherche Médicale (INSERM), REACTing-INSERM and the University of Paris Cité. A.N.S. is supported by European Union's Horizon Health research and innovation program under grant agreement No. 101057100, project UNDINE. S.G.T. is supported by an Investigator Grant awarded by the National Health and Medical Research Council of Australia, and a University of New South Wales Sydney COVID Rapid Response Initiative grant.

The study was supported by the ORCHESTRA project, which has received funding from the European Union's Horizon 2020 research and innovation program under grant agreement No. 10101616. The French COVID Cohort study group was sponsored by Inserm and supported by the REACTing consortium and by a grant from the French Ministry of Health (Grant PHRC 20-0424). The Cov-Contact Cohort was supported by the REACTing consortium, the French Ministry of Health, European Commission (Grant RECOVER WP 6). The COVIDeF study group was supported by the French Ministry of Health and Fondation AP-HP Programme Hospitalier de Recherche Clinique (PHRC COVID-19-20-0048). Y.Z. is supported by the Intramural Research Program of the National Institute of Allergy and Infectious Diseases, NIH. This project has received funding from the Regione Lazio (Research Group Projects 2020) No. A0375-2020-36663, GecoBiomark, the European Research Council (ERC) under the European Union's Horizon 2020 research and innovation program (grant agreement no. 948959), the Swiss National Science Foundation (grant # 310030L_197721 to J.F.), and ERN-RITA. The Canarian Sequencing Hub is funded by Instituto de Salud Carlos III (COV20_01333, COV20_01334, and PI20/00876) and Spanish Ministry of Science and Innovation (RTC-2017-6471-1; AEI/FEDER, UE), co-financed by the European Regional Development Funds, "A way of making Europe" from the European Union, Cabildo Insular de Tenerife (CGIEU0000219140 and "Apuestas científicas del ITER para colaborar en la lucha contra la COVID-19"). This work was funded, at least in part, by grants AJF202019 and AJF20259 from Al Jalila Foundation, Dubai, United Arab Emirates. Sample processing at IrsiCaixa was possible thanks to the crowdfunding initiative YoMeCorono. We thank I. Erkizia, E. Grau, M. Massanella, and J. Guitart from the IrsiCaixa and Hospital Germans Trias i Pujol (Badalona, Spain) for sample collection, handling, and processing.

We thank the patients and their families for agreeing to participate in our research.

We thank Helen C. Su from the NIAID (Bethesda, MD, USA) and all members of the consortia listed in supplemental information.

Author contributions

A.M., A.Cobat, and A.B. performed computational analysis. A.M., E.T.C., I.N., E.B., R.S.T., K.M.S.B., Y.Z., I.N.B., M.K., A.Catchpole, J.B.L., C.L.D., V.S.S., A.Cobat, and A.B. performed or supervised experiments, generated and analyzed data, and contributed to the manuscript by providing figures and tables. S.G.T., A.N.S., J.G., C.B., G.G., F.T., P.H., S.Y.Z., Q.Z., C.C., J.F., J.J.G., V.S.S., and the consortium collaborators evaluated and recruited patients and/or controls. A.M., L.A., J.L.C., A.Cobat, and A.B. wrote the manuscript. C.C., J.J.G., L.A., J.L.C., A.Cobat, and A.B. supervised the project. All authors edited the manuscript. All authors read and approved the final manuscript.

Declaration of interests

E.T.C., K.M.S.B., and A.B. are employees of Helix.

Received: December 4, 2023
Accepted: April 23, 2024

Web resources

HIBAG software and models: https://hibag.s3.amazonaws.com/hlares_index.html

Allele frequency Net Database: https://github.com/slowkow/allelefrequencies

HLA alleles distribution in 1000 Genomes Project: http://ftp.1000genomes.ebi.ac.uk/vol1/ftp/data_collections/HLA_types/

Genetic Association Study Power Calculator: https://csg.sph.umich.edu/abecasis/gas_power_calculator

References

1. Asano, T., Boisson, B., Onodi, F., Matuozzo, D., Moncada-Velez, M., Maglorius Renkilaraj, M.R.L., Zhang, P., Meertens, L., Bolze, A., Materna, M., et al. (2021). X-linked recessive TLR7 deficiency in ~1% of men under 60 years old with life-threatening COVID-19. Sci. Immunol. *6*, eabl4348. https://doi.org/10.1126/sciimmunol.abl4348.
2. Kousathanas, A., Pairo-Castineira, E., Rawlik, K., Stuckey, A., Odhams, C.A., Walker, S., Russell, C.D., Malinauskas, T., Wu, Y., Millar, J., et al. (2022). Whole-genome sequencing reveals host factors underlying critical COVID-19. Nature *607*, 97–103. https://doi.org/10.1038/s41586-022-04576-6.
3. Cobat, A., Zhang, Q., COVID Human Genetic Effort, Abel, L., Casanova, J.L., and Fellay, J. (2023). Human Genomics of COVID-19 Pneumonia: Contributions of Rare and Common Variants. Annu. Rev. Biomed. Data Sci. *6*, 465–486. https://doi.org/10.1146/annurev-biodatasci-020222-021705.
4. Severe Covid-19 GWAS Group, Ellinghaus, D., Degenhardt, F., Bujanda, L., Buti, M., Albillos, A., Fernández, J., Fernández, J., Prati, D., Baselli, G., et al. (2020). Genomewide Association Study of Severe Covid-19 with Respiratory Failure. N. Engl. J. Med. *383*, 1522–1534. https://doi.org/10.1056/NEJMoa2020283.
5. Kanai, M., Andrews, S.J., Cordioli, M., Stevens, C., Neale, B.M., Daly, M., Ganna, A., Pathak, G.A., Iwasaki, A., Karjalainen, J., et al. (2023). A second update on mapping the human genetic architecture of COVID-19. Nature *621*, E7–E26. https://doi.org/10.1038/s41586-023-06355-3.
6. Roberts, G.H.L., Partha, R., Rhead, B., Knight, S.C., Park, D.S., Coignet, M.V., Zhang, M., Berkowitz, N., Turrisini, D.A., Gaddis, M., et al. (2022). Expanded COVID-19 phenotype definitions reveal distinct patterns of genetic association and protective effects. Nat. Genet. *54*, 374–381. https://doi.org/10.1038/s41588-022-01042-x.
7. Matuozzo, D., Talouarn, E., Marchal, A., Zhang, P., Manry, J., Seeleuthner, Y., Zhang, Y., Bolze, A., Chaldebas, M., Milisavljevic, B., et al. (2023). Rare predicted loss-of-function variants of type I IFN immunity genes are associated with life-threatening COVID-19. Genome Med. *15*, 22. https://doi.org/10.1186/s13073-023-01173-8.
8. Zhang, Q., Bastard, P., Liu, Z., Le Pen, J., Moncada-Velez, M., Chen, J., Ogishi, M., Sabli, I.K.D., Hodeib, S., Korol, C., et al. (2020). Inborn errors of type I IFN immunity in patients with life-threatening COVID-19. Science *370*, eabd4570. https://doi.org/10.1126/science.abd4570.
9. Zhang, Q., Matuozzo, D., Le Pen, J., Lee, D., Moens, L., Asano, T., Bohlen, J., Liu, Z., Moncada-Velez, M., Kendir-Demirkol, Y., et al. (2022). Recessive inborn errors of type I IFN immunity in children with COVID-19 pneumonia. J. Exp. Med. *219*, e20220131. https://doi.org/10.1084/jem.20220131.
10. Bastard, P., Gervais, A., Le Voyer, T., Rosain, J., Philippot, Q., Manry, J., Michailidis, E., Hoffmann, H.-H., Eto, S., Garcia-Prat, M., et al. (2021). Autoantibodies neutralizing type I IFNs are present in ~4% of uninfected individuals over 70 years old and account for ~20% of COVID-19 deaths. Sci. Immunol. *6*, eabl4340. https://doi.org/10.1126/sciimmunol.abl4340.
11. Zhang, Q., Bastard, P., COVID Human Genetic Effort, Cobat, A., and Casanova, J.L. (2022). Human genetic and immunological determinants of critical COVID-19 pneumonia. Nature *603*, 587–598. https://doi.org/10.1038/s41586-022-04447-0.
12. Bastard, P., Rosen, L.B., Zhang, Q., Michailidis, E., Hoffmann, H.-H., Zhang, Y., Dorgham, K., Philippot, Q., Rosain, J., Béziat, V., et al. (2020). Autoantibodies against type I IFNs in patients with life-threatening COVID-19. Science *370*, eabd4585. https://doi.org/10.1126/science.abd4585.
13. Weiner, J., Suwalski, P., Holtgrewe, M., Rakitko, A., Thibeault, C., Müller, M., Patriki, D., Quedenau, C., Krüger, U., Ilinsky, V., et al. (2021). Increased risk of severe clinical course of COVID-19 in carriers of HLA-C*04:01. eClinicalMedicine *40*, 101099. https://doi.org/10.1016/j.eclinm.2021.101099.
14. Fakhkhari, M., Caidi, H., and Sadki, K. (2023). HLA alleles associated with COVID-19 susceptibility and severity in different populations: a systematic review. Egypt. J. Med. Hum. Genet. *24*, 10. https://doi.org/10.1186/s43042-023-00390-5.
15. Augusto, D.G., Murdolo, L.D., Chatzileontiadou, D.S.M., Sabatino, J.J., Yusufali, T., Peyser, N.D., Butcher, X., Kizer, K., Guthrie, K., Murray, V.W., et al. (2023). A common allele of HLA is associated with asymptomatic SARS-CoV-2 infection. Nature *620*, 128–136. https://doi.org/10.1038/s41586-023-06331-x.
16. Buckley, P.R., Lee, C.H., Pereira Pinho, M., Ottakandathil Babu, R., Woo, J., Antanaviciute, A., Simmons, A., Ogg, G., and Koohy, H. (2022). HLA-dependent variation in SARS-CoV-2 CD8 + T cell cross-reactivity with human coronaviruses. Immunology *166*, 78–103. https://doi.org/10.1111/imm.13451.
17. Bolze, A., Neveux, I., Schiabor Barrett, K.M., White, S., Isaksson, M., Dabe, S., Lee, W., Grzymski, J.J., Washington, N.L., and Cirulli, E.T. (2022). HLA-A*03:01 is associated with increased risk of fever, chills, and stronger side effects from Pfizer-BioNTech COVID-19 vaccination. HGG Adv. *3*, 100084. https://doi.org/10.1016/j.xhgg.2021.100084.
18. Marshall, J.C., Murthy, S., Diaz, J., Adhikari, N.K., Angus, D.C., Arabi, Y.M., Baillie, K., Bauer, M., Berry, S., Blackwood, B., et al. (2020). A minimal common outcome measure set for COVID-19 clinical research. Lancet Infect. Dis. *20*, e192–e197. https://doi.org/10.1016/S1473-3099(20)30483-7.
19. Zhou, J., Singanayagam, A., Goonawardane, N., Moshe, M., Sweeney, F.P., Sukhova, K., Killingley, B., Kalinova, M., Mann, A.J., Catchpole, A.P., et al. (2023). Viral emissions into the air and environment after SARS-CoV-2 human challenge: a phase 1, open label, first-in-human study. Lancet. Microbe *4*, e579–e590. https://doi.org/10.1016/S2666-5247(23)00101-5.
20. Wagstaffe, H.R., Thwaites, R.S., Reynaldi, A., Sidhu, J.K., McKendry, R., Ascough, S., Papargyris, L., Collins, A.M., Xu, J., Lemm, N.-M., et al. (2024). Mucosal and systemic immune correlates of viral control after SARS-CoV-2 infection

challenge in seronegative adults. Sci. Immunol. *9*, eadj9285. https://doi.org/10.1126/sciimmunol.adj9285.
21. Cirulli, E.T., White, S., Read, R.W., Elhanan, G., Metcalf, W.J., Tanudjaja, F., Fath, D.M., Sandoval, E., Isaksson, M., Schlauch, K.A., et al. (2020). Genome-wide rare variant analysis for thousands of phenotypes in over 70,000 exomes from two cohorts. Nat. Commun. *11*, 542. https://doi.org/10.1038/s41467-020-14288-y.
22. hail: Cloud-native genomic dataframes and batch computing. Github. https://github.com/hail-is/hail.
23. Alexander, D.H., Novembre, J., and Lange, K. (2009). Fast model-based estimation of ancestry in unrelated individuals. Genome Res. *19*, 1655–1664. https://doi.org/10.1101/gr.094052.109.
24. Belkadi, A., Pedergnana, V., Cobat, A., Itan, Y., Vincent, Q.B., Abhyankar, A., Shang, L., El Baghdadi, J., Bousfiha, A., et al.; Exome/Array Consortium (2016). Whole-exome sequencing to analyze population structure, parental inbreeding, and familial linkage. Proc. Natl. Acad. Sci. USA *113*, 6713–6718. https://doi.org/10.1073/pnas.1606460113.
25. Karczewski, K.J., Francioli, L.C., Tiao, G., Cummings, B.B., Alföldi, J., Wang, Q., Collins, R.L., Laricchia, K.M., Ganna, A., Birnbaum, D.P., et al. (2020). The mutational constraint spectrum quantified from variation in 141,456 humans. Nature *581*, 434–443. https://doi.org/10.1038/s41586-020-2308-7.
26. Zheng, X., Shen, J., Cox, C., Wakefield, J.C., Ehm, M.G., Nelson, M.R., and Weir, B.S. (2014). HIBAG—HLA genotype imputation with attribute bagging. Pharmacogenomics J. *14*, 192–200. https://doi.org/10.1038/tpj.2013.18.
27. Dilthey, A.T., Mentzer, A.J., Carapito, R., Cutland, C., Cereb, N., Madhi, S.A., Rhie, A., Koren, S., Bahram, S., McVean, G., and Phillippy, A.M. (2019). HLA*LA—HLA typing from linearly projected graph alignments. Bioinformatics *35*, 4394–4396. https://doi.org/10.1093/bioinformatics/btz235.
28. Karnes, J.H., Shaffer, C.M., Bastarache, L., Gaudieri, S., Glazer, A.M., Steiner, H.E., Mosley, J.D., Mallal, S., Denny, J.C., Phillips, E.J., and Roden, D.M. (2017). Comparison of HLA allelic imputation programs. PLoS One *12*, e0172444. https://doi.org/10.1371/journal.pone.0172444.
29. Claeys, A., Merseburger, P., Staut, J., Marchal, K., and Van den Eynden, J. (2023). Benchmark of tools for in silico prediction of MHC class I and class II genotypes from NGS data. BMC Genom. *24*, 1–14. https://doi.org/10.1186/s12864-023-09351-z.
30. Valencia, A., Vergara, C., Thio, C.L., Vince, N., Douillard, V., Grifoni, A., Cox, A.L., Johnson, E.O., Kral, A.H., Goedert, J.J., et al. (2022). Trans-ancestral fine-mapping of MHC reveals key amino acids associated with spontaneous clearance of hepatitis C in HLA-DQβ1. Am. J. Hum. Genet. *109*, 299–310. https://doi.org/10.1016/j.ajhg.2022.01.001.
31. Mbatchou, J., Barnard, L., Backman, J., Marcketta, A., Kosmicki, J.A., Ziyatdinov, A., Benner, C., O'Dushlaine, C., Barber, M., Boutkov, B., et al. (2021). Computationally efficient whole-genome regression for quantitative and binary traits. Nat. Genet. *53*, 1097–1103. https://doi.org/10.1038/s41588-021-00870-7.
32. Willer, C.J., Li, Y., and Abecasis, G.R. (2010). METAL: fast and efficient meta-analysis of genomewide association scans. Bioinformatics *26*, 2190–2191. https://doi.org/10.1093/bioinformatics/btq340.
33. Skol, A.D., Scott, L.J., Abecasis, G.R., and Boehnke, M. (2006). Joint analysis is more efficient than replication-based analysis for two-stage genome-wide association studies. Nat. Genet. *38*, 209–213. https://doi.org/10.1038/ng1706.
34. Lee, D., Le Pen, J., Yatim, A., Dong, B., Aquino, Y., Ogishi, M., Pescarmona, R., Talouarn, E., Rinchai, D., Zhang, P., et al. (2023). Inborn errors of OAS–RNase L in SARS-CoV-2–related multisystem inflammatory syndrome in children. Science *379*, eabo3627. https://doi.org/10.1126/science.abo3627.
35. Andreakos, E., Abel, L., Vinh, D.C., Kaja, E., Drolet, B.A., Zhang, Q., O'Farrelly, C., Novelli, G., Rodríguez-Gallego, C., Haerynck, F., et al. (2022). A global effort to dissect the human genetic basis of resistance to SARS-CoV-2 infection. Nat. Immunol. *23*, 159–164. https://doi.org/10.1038/s41590-021-01030-z.
36. Castro-Santos, P., Rojas-Martinez, A., Riancho, J.A., Lapunzina, P., Flores, C., Carracedo, Á., Díaz-Peña, R.; and Scourge Cohort Group (2023). HLA-A*11:01 and HLA-C*04:01 are associated with severe COVID-19. HLA *102*, 731–739. https://doi.org/10.1111/tan.15160.
37. Bolze, A., Luo, S., White, S., Cirulli, E.T., Wyman, D., Dei Rossi, A., Machado, H., Cassens, T., Jacobs, S., Schiabor Barrett, K.M., et al. (2022). SARS-CoV-2 variant Delta rapidly displaced variant Alpha in the United States and led to higher viral loads. Cell Rep. Med. *3*, 100564. https://doi.org/10.1016/j.xcrm.2022.100564.
38. Feero, W.G., Steiner, R.D., Slavotinek, A., Faial, T., Bamshad, M.J., Austin, J., Korf, B.R., Flanagin, A., and Bibbins-Domingo, K. (2024). Guidance on use of race, ethnicity, and geographic origin as proxies for genetic ancestry groups in biomedical publications. HGG Adv. *5*, 100282. https://doi.org/10.1016/j.xhgg.2024.100282.
39. Niemi, M.E.K., Daly, M.J., and Ganna, A. (2022). The human genetic epidemiology of COVID-19. Nat. Rev. Genet. *23*, 533–546. https://doi.org/10.1038/s41576-022-00478-5.
40. Clohisey, S., and Baillie, J.K. (2019). Host susceptibility to severe influenza A virus infection. Crit. Care *23*, 303. https://doi.org/10.1186/s13054-019-2566-7.
41. Egeskov-Cavling, A.M., van Wijhe, M., Yakimov, V., Johannesen, C.K., Pollard, A.J., Trebbien, R., Bybjerg-Grauholm, J., Fischer, T.K.; and RESCEU investigators (2023). Genome-Wide Association study of susceptibility to respiratory syncytial virus hospitalization in young children < 5 years of age. J. Infect. Dis. jiad370. https://doi.org/10.1093/infdis/jiad370.
42. Blackwell, J.M., Jamieson, S.E., and Burgner, D. (2009). HLA and Infectious Diseases. Clin. Microbiol. Rev. *22*, 370–385. https://doi.org/10.1128/cmr.00048-08.
43. Duggal, P., Thio, C.L., Wojcik, G.L., Goedert, J.J., Mangia, A., Latanich, R., Kim, A.Y., Lauer, G.M., Chung, R.T., Peters, M.G., et al. (2013). Genome-Wide Association Study of Spontaneous Resolution of Hepatitis C Virus Infection: Data From Multiple Cohorts. Ann. Intern. Med. *158*, 235–245. https://doi.org/10.7326/0003-4819-158-4-201302190-00003.
44. Dallmann-Sauer, M., Fava, V.M., Gzara, C., Orlova, M., Van Thuc, N., Thai, V.H., Alcaïs, A., Abel, L., Cobat, A., and Schurr, E. (2020). The complex pattern of genetic associations of leprosy with HLA class I and class II alleles can be reduced to four amino acid positions. PLoS Pathog. *16*, e1008818. https://doi.org/10.1371/journal.ppat.1008818.
45. McLaren, P.J., and Fellay, J. (2021). HIV-1 and human genetic variation. Nat. Rev. Genet. *22*, 645–657. https://doi.org/10.1038/s41576-021-00378-0.
46. Fellay, J., Frahm, N., Shianna, K.V., Cirulli, E.T., Casimiro, D.R., Robertson, M.N., Haynes, B.F., Geraghty, D.E., McElrath,

M.J., Goldstein, D.B., et al. (2011). Host Genetic Determinants of T Cell Responses to the MRKAd5 HIV-1 gag/pol/nef Vaccine in the Step Trial. J. Infect. Dis. *203*, 773–779. https://doi.org/10.1093/infdis/jiq125.

47. Scepanovic, P., Alanio, C., Hammer, C., Hodel, F., Bergstedt, J., Patin, E., Thorball, C.W., Chaturvedi, N., Charbit, B., Abel, L., et al. (2018). Human genetic variants and age are the strongest predictors of humoral immune responses to common pathogens and vaccines. Genome Med. *10*, 59. https://doi.org/10.1186/s13073-018-0568-8.

48. Mentzer, A.J., O'Connor, D., Bibi, S., Chelysheva, I., Clutterbuck, E.A., Demissie, T., Dinesh, T., Edwards, N.J., Felle, S., Feng, S., et al. (2023). Human leukocyte antigen alleles associate with COVID-19 vaccine immunogenicity and risk of breakthrough infection. Nat. Med. *29*, 147–157. https://doi.org/10.1038/s41591-022-02078-6.

HGGA, Volume *5*

Supplemental information

Lack of association between classical HLA genes and asymptomatic SARS-CoV-2 infection

Astrid Marchal, Elizabeth T. Cirulli, Iva Neveux, Evangelos Bellos, Ryan S. Thwaites, Kelly M. Schiabor Barrett, Yu Zhang, Ivana Nemes-Bokun, Mariya Kalinova, Andrew Catchpole, Stuart G. Tangye, András N. Spaan, Justin B. Lack, Jade Ghosn, Charles Burdet, Guy Gorochov, Florence Tubach, Pierre Hausfater, COVID Human Genetic Effort, COVIDeF Study Group, French COVID Cohort Study Group, CoV-Contact Cohort, COVID-STORM Clinicians, COVID Clinicians, Orchestra Working Group, Amsterdam UMC COVID-19 Biobank, NIAID-USUHS COVID Study Group, Clifton L. Dalgard, Shen-Ying Zhang, Qian Zhang, Christopher Chiu, Jacques Fellay, Joseph J. Grzymski, Vanessa Sancho-Shimizu, Laurent Abel, Jean-Laurent Casanova, Aurélie Cobat, and Alexandre Bolze

Table of content

Figure S1: COVID-19 symptoms in a US prospective cohort.

Figure S2: COVID-19 symptoms in the CHGE cohort.

Figure S3: Power curves: replication.

Figure S4: Seasonal CoV antibodies in the SARS-CoV-2 Human Challenge Characterisation Study.

Figure S5: Frequency of HLA-B*15:01 allele.

Figure S6: Power curves: HLA-WAS.

Supplemental methods: Ethics approval and consent to participate.

Table S1: Demographics of both cohorts.

Table S2: HLA-WAS US prospective cohort - asymptomatic '0 symptoms'.

Table S3: HLA-WAS US prospective cohort - asymptomatic 'Max 1 day'.

Table S4: HLA-WAS US prospective cohort - asymptomatic 'Max 2 days'.

Table S5: HLA-WAS CHGE cohort - asymptomatic vs severe and critical.

Table S6: HLA-WAS CHGE cohort - asymptomatic vs all symptomatic.

Table S7: HLA-WAS CHGE cohort - asymptomatic vs mild and moderate.

Table S8: Meta-analysis M1: US asymptomatic '0 symptoms' + CHGE severe critical.

Table S9: Meta-analysis M2: US asymptomatic 'Max 1 day' + CHGE all.

Table S10: Meta-analysis M3: US asymptomatic 'Max 2 days' + CHGE mild moderate.

Table S11: HLA-B*15:01 allele counts.

Figure S1

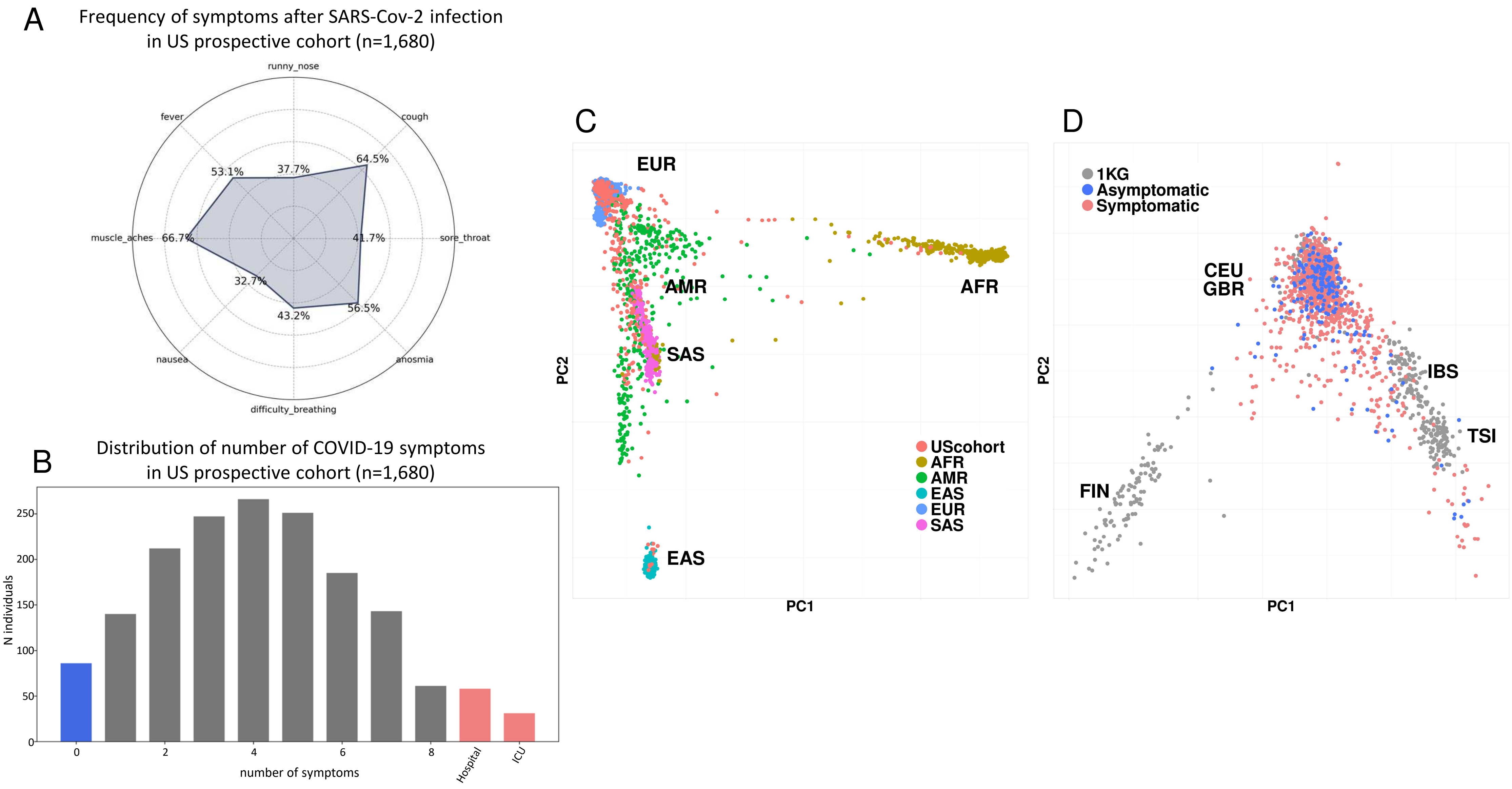

Figure S1: COVID-19 symptoms in a US prospective cohort.

A: Radar plot showing the % of infected individuals reporting each symptom.

B: Distribution of the number of symptoms per individual infected with SARS-CoV-2. Patients who had to be hospitalized or admitted to the ICU are represented separately (pink bars). Infected individuals who reporting no symptoms are represented by the blue bar.

C: PCA plot displaying the US prospective cohort (orange dots) overlapped with 1000 Genomes Project samples labeled with their known ancestry (AFR: African, AMR: American, EAS: East Asian, EUR: European, SAS: South Asian).

D: PCA plot displaying the European genetic ancestry subsets of the US prospective cohort and from 1000 Genomes Projects. Origin of 1000 Genomes Project (1KG) samples are indicated (CEU: Utah residents with Northern and Western European ancestry, FIN: Finnish in Finland, GBR: British in England and Scotland, IBS: Iberian population in Spain, TSI: Toscani in Italy).

Figure S2

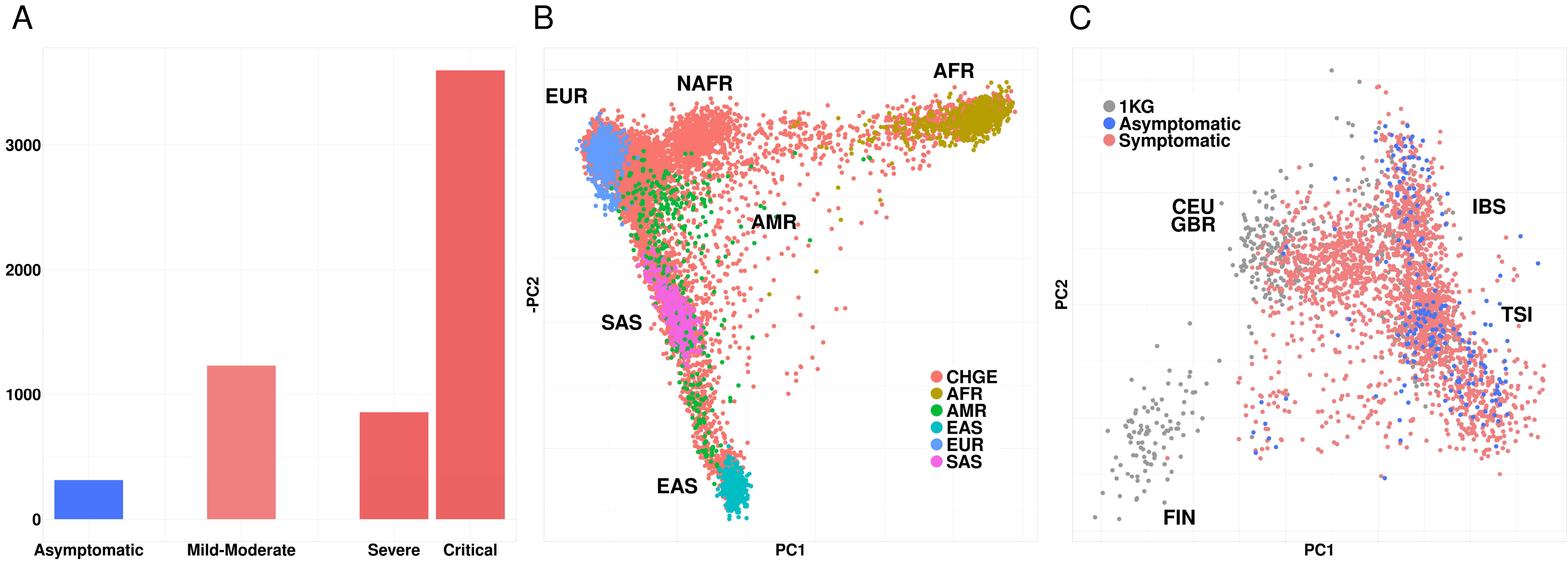

Figure S2: COVID-19 symptoms in the CHGE cohort.
A: Distribution of the number of individuals per category of severity.
B: PCA plot displaying the CHGE cohort (orange dots) overlapped with the samples from 1000 Genomes Project. Ancestry of 100 Genomes Project samples are indicated (AFR: African, AMR: American, EAS: East Asian, EUR: European, NAFR: North African, SAS: South Asian).
C: PCA plot displaying the European genetic ancestry subsets of the CHGE cohort and from 1000 Genomes Projects. Origin of 1000 Genomes Project (1KG) samples are indicated (CEU: Utah residents with Northern and Western European ancestry, FIN: Finnish in Finland, GBR: British in England and Scotland, IBS: Iberian population in Spain, TSI: Toscani in Italy).

Figure S3

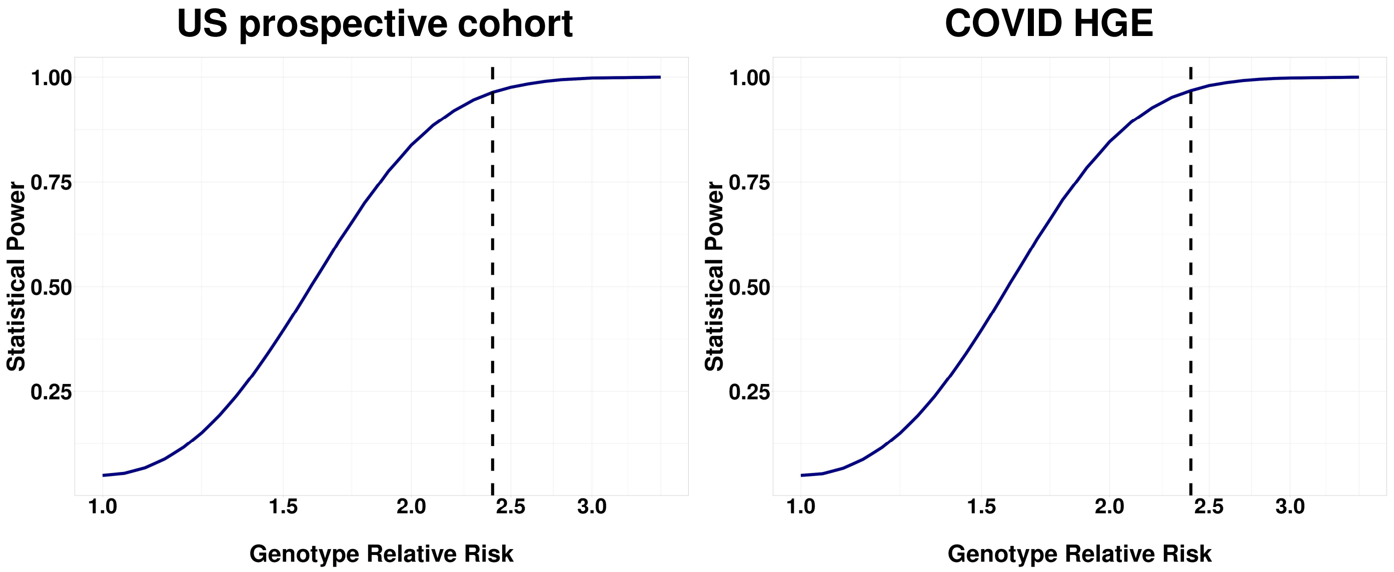

Figure S3: Power curves: replication.
Statistical power as a function of genotype relative risk, calculated under a dominant inheritance model, with a *HLA-B*15:01* frequency of 0.05, a prevalence of asymptomatic infection of 0.1, and a p-value threshold of 0.05 in a context of replication.
Curves plotted with the Genetic Association Study Power Calculator (https://csg.sph.umich.edu/abecasis/gas_power_calculator). The dotted line represent the Odds ratio obtained by Augusto, Murdolo & Chatzileontiadou et al. (OR=2.4).

Figure S4

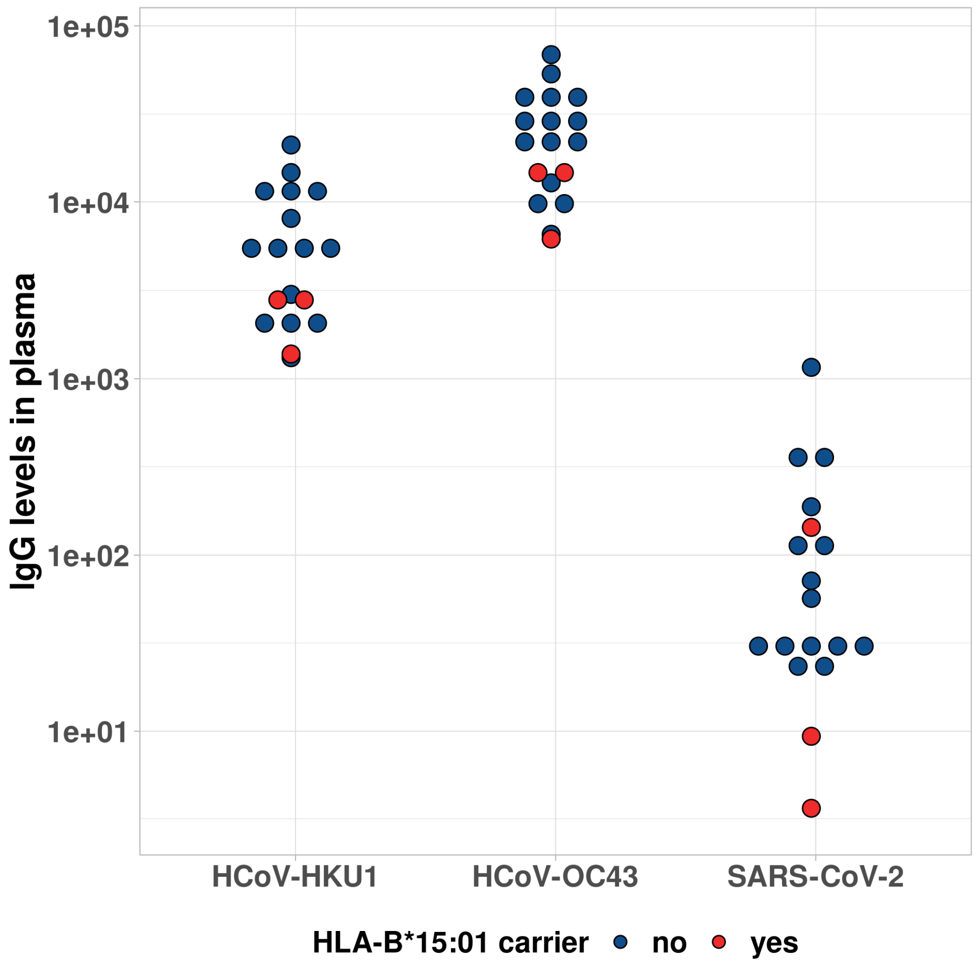

Figure S4: Seasonal CoV antibodies in the SARS-CoV-2 Human Challenge Characterisation Study.
Plasma IgG was quantified in baseline samples from the SARS-CoV-2 human challenge characterisation study participants (Infected, n=17) for HKU1-CoV Spike protein, OC43-CoV Spike protein and SARS-CoV-2 Spike protein as a negative control. Carriers of HLA-B*15:01 are indicated in red. Arbitrary units per milliliter.

Figure S5

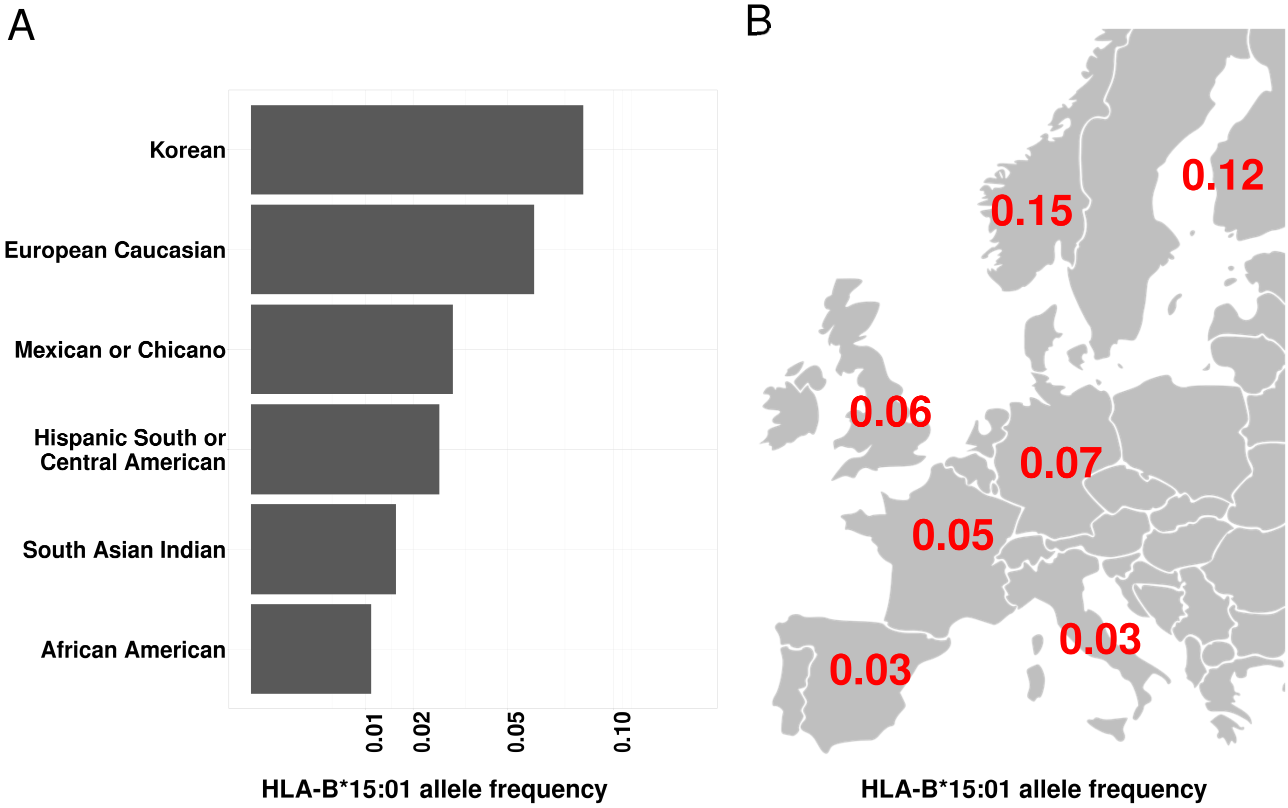

Figure S5: Frequency of *HLA-B*15:01* allele:
(A) for the various subpopulations in the US. Names are those used by the USA National Marrow Donor Program (NMDP). (B) for various countries in Europe. Data from Allele Frequency Net Database (www.allelefrequencies.net) and 1000 Genomes Project.

Figure S6

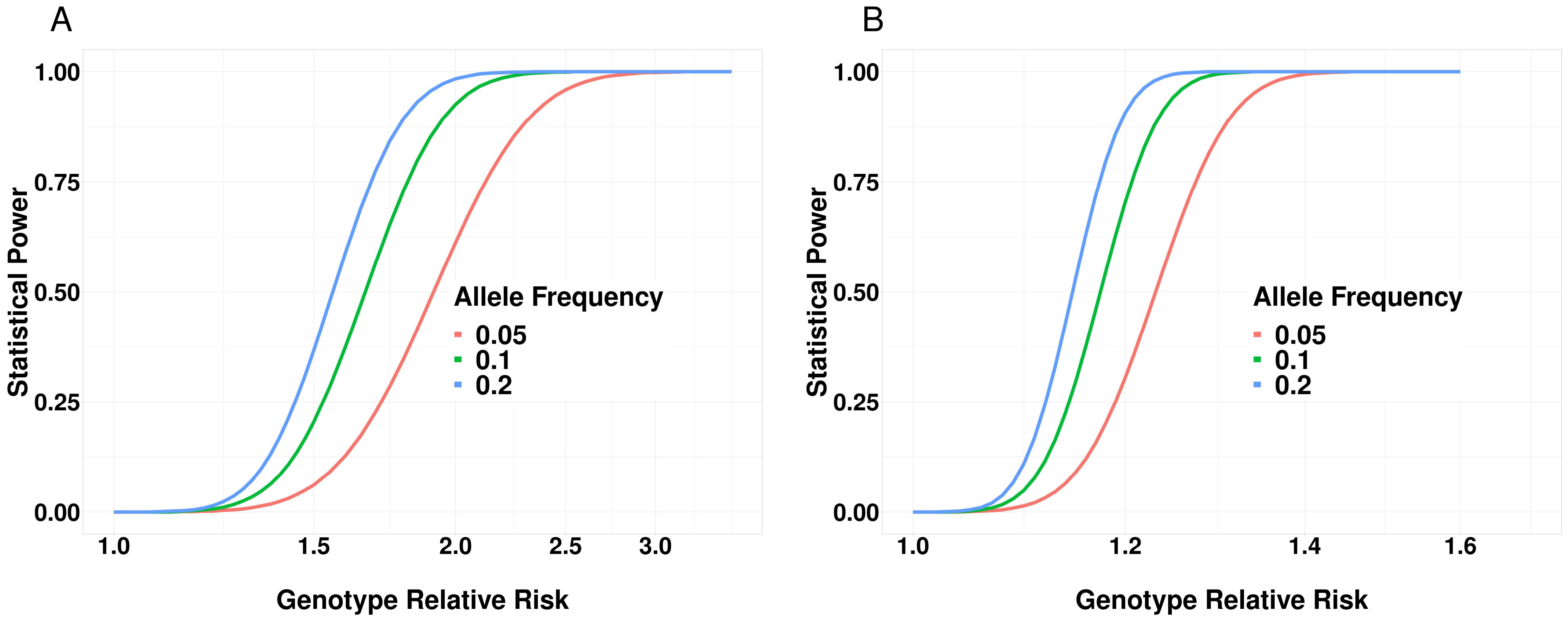

Figure S6: Power curves: HLA-WAS.
Statistical power as a function of genotype relative risk, calculated under a dominant inheritance model, with various allele frequencies (0.05, 0.1, 0.2), a prevalence of asymptomatic infection of 0.1, a p-value threshold of 0.0005. A: in a combined cohort of 400 asymptomatic cases and 3,000 symptomatic controls. B: in a hypothetical cohort of 4,000 asymptomatic cases and 30,000 symptomatic controls.
Curves plotted with the Genetic Association Study Power Calculator (https://csg.sph.umich.edu/abecasis/gas_power_calculator).

Supplemental methods

Ethics approval and consent to participate

US prospective cohort

All participants enrolled in the US prospective cohort provided written informed consent for participation and were recruited through protocols conforming to local ethics requirements. The Helix DNA Discovery Project was reviewed and approved by the Western Institutional Review Board. For the Healthy Nevada Project (HNP), the University of Nevada, Reno Institutional Review Board approved the study (project 956068-12). The procedures followed were in accordance with ethical standards, and appropriate informed consent was obtained.

CHGE cohort

All the participants enrolled in the CHGE cohort provided written informed consent for participation and were recruited through protocols conforming to local ethics requirements. For patients enrolled in the French COVID cohort (ClinicalTrials.gov NCT04262921), ethics approval was obtained from the Comité de Protection des Personnes Ile De France VI (ID RCB, 2020-A00256-33) or the Ethics Committee of Erasme Hospital (P2020/203). For participants enrolled in the COV-Contact study (ClinicalTrials.gov NCT04259892), ethics approval was obtained from the CPP IDF VI (ID RCB, 2020-A00280-39). For patients enrolled in the Italian cohort, ethics approval was obtained from the University of Milano-Bicocca School of Medicine, San Gerardo Hospital, Monza–Ethics Committee of the National Institute of Infectious Diseases Lazzaro Spallanzani (84/2020) (Italy), and the Comitato Etico Provinciale (NP 4000–Studio CORONAlab). STORM-Health care workers were enrolled in the STudio OsseRvazionale sullo screening dei lavoratori ospedalieri per COVID-19 (STORM-HCW) study, with approval from the local institutional review board (IRB) obtained on June 18, 2020. Patients and relatives from San Raffaele Hospital (Milan) were enrolled in COVID-BioB/Gene-COVID protocols and, for additional studies, TIGET-06, with the approval of the local ethics committee. Patients and relatives from Rome were enrolled in Protocol no. 50/20 (Tor Vergata University Hospital). Informed consent was obtained from each patient. For the patients enrolled in the COVIDeF Study Group (ClinicalTrials.gov NCT04352348), ethics approval was obtained from the Comité de Protection des Personnes Ile de France XI (ID RCB, 2020-A00754-35). For patients enrolled in Spain, the study was approved by the Committee for Ethical Research of the Infanta Leonor University Hospital, code 008–20; the Committee for Ethical Research of the 12 de Octubre University Hospital, code 16/368; the Bellvitge University Hospital, code PR127/20; the University Hospital of Gran Canaria Dr. Negrín, code 2020–200-1 COVID-19; and the Vall d'Hebron University Hospital, code PR(AMI)388/2016. Anonymized samples were sequenced at the National Institute of Allergy and Infectious Diseases (NIAID) through the Uniformed Services University of the Health Sciences (USUHS)/the American Genome Center (TAGC) under nonhuman subject research conditions; no additional IRB consent was required at the National Institutes of Health (NIH). For patients enrolled in the Swedish COVID cohort, ethics approval was obtained from the Swedish Ethical Review Agency (2020–01911 05).
